# A Critical Review of the Antimicrobial and Antibiofilm Activities of Green-Synthesized Plant-Based Metallic Nanoparticles

**DOI:** 10.3390/nano12111841

**Published:** 2022-05-27

**Authors:** Miryam M. Luzala, Claude K. Muanga, Joseph Kyana, Justin B. Safari, Eunice N. Zola, Grégoire V. Mbusa, Yannick B. Nuapia, Jean-Marie I. Liesse, Christian I. Nkanga, Rui W. M. Krause, Aistė Balčiūnaitienė, Patrick B. Memvanga

**Affiliations:** 1 Laboratory of Pharmaceutics and Phytopharmaceutical Drug Development, Faculty of Pharmaceutical Sciences, University of Kinshasa, Kinshasa XI B.P. 212, Democratic Republic of the Congo; miryamluzala.ml@gmail.com (M.M.L.); manahaimpharma@gmail.com (C.K.M.); eunicezola6@gmail.com (E.N.Z.); christian.nkanga@unikin.ac.cd (C.I.N.); 2 Department of Pharmacy, Faculty of Medecine and Pharmacy, University of Kisangani, Kisangani XI B.P. 2012, Democratic Republic of the Congo; josephkyana2@gmail.com; 3 Department of Pharmacy, Faculty of Pharmaceutical Sciences and Public Health, Official University of Bukavu, Bukavu B.P. 570, Democratic Republic of the Congo; safaribazibuhejustin@gmail.com; 4 Department of Chemistry, Faculty of Science, Rhodes University, P.O. Box 94, Makhana 6140, South Africa; 5 Centre Universitaire de Référence de Surveillance de la Résistance aux Antimicrobiens (CURS-RAM), Faculty of Pharmaceutical Sciences, University of Kinshasa, Kinshasa XI B.P. 212, Democratic Republic of the Congo; gregvihembo@gmail.com (G.V.M.); liesseiyamba@gmail.com (J.-M.I.L.); 6 Laboratory of Experimental and Pharmaceutical Microbiology, Faculty of Pharmaceutical Sciences, University of Kinshasa, Kinshasa XI B.P. 212, Democratic Republic of the Congo; 7 Laboratory of Toxicology, Faculty of Pharmaceutical Sciences, University of Kinshasa, Kinshasa XI B.P. 212, Democratic Republic of the Congo; yannicknuapia9@gmail.com; 8 Center for Chemico- and Bio-Medicinal Research (CCBR), Faculty of Science, Rhodes University, P.O. Box 94, Makhana 6140, South Africa; 9 Lithuanian Research Centre for Agriculture and Forestry, Institute of Horticulture, 54333 Babtai, Lithuania; aiste.balciunaitiene@lammc.lt; 10 Centre de Recherche et d’Innovation Technologique en Environnement et en Sciences de la Santé (CRITESS), University of Kinshasa, Kinshasa XI B.P. 212, Democratic Republic of the Congo

**Keywords:** plant-based synthesis, metallic nanoparticles, antimicrobial and antibiofilm activities, drug-susceptibility testing methods, influencing factors

## Abstract

Metallic nanoparticles (MNPs) produced by green synthesis using plant extracts have attracted huge interest in the scientific community due to their excellent antibacterial, antifungal and antibiofilm activities. To evaluate these pharmacological properties, several methods or protocols have been successfully developed and implemented. Although these protocols were mostly inspired by the guidelines from national and international regulatory bodies, they suffer from a glaring absence of standardization of the experimental conditions. This situation leads to a lack of reproducibility and comparability of data from different study settings. To minimize these problems, guidelines for the antimicrobial and antibiofilm evaluation of MNPs should be developed by specialists in the field. Being aware of the immensity of the workload and the efforts required to achieve this, we set out to undertake a meticulous literature review of different experimental protocols and laboratory conditions used for the antimicrobial and antibiofilm evaluation of MNPs that could be used as a basis for future guidelines. This review also brings together all the discrepancies resulting from the different experimental designs and emphasizes their impact on the biological activities as well as their interpretation. Finally, the paper proposes a general overview that requires extensive experimental investigations to set the stage for the future development of effective antimicrobial MNPs using green synthesis.

## 1. Introduction

Nanotechnology involves science, engineering and technology that considers matter at atomic, molecular or supramolecular levels to produce nanometric materials and nanosystems with improved properties, such as high surface-to-volume ratios and high dispersion in solution [1]. With sizes typically ranging between 1 and 100 nm in at least one dimension, nanomaterials and nanosystems can be synthesized by chemical, physical and/or biological methods [2,3].

In comparison to chemical and physical methods that can involve costly and toxic chemicals [2,4], the biological synthesis pathway, based on the use of biological sources (e.g., plants, bacteria, fungi and algae) represents an attractive option [5,6]. However, though these biological methods do not involve toxic chemicals in the preparation protocols, the microbial production of metal nanoparticles is highly demanding, time consuming and costly, requiring technology and practical microbiological experience to ensure cell culture and nanoparticle purification under aseptic conditions [7,8].

In contrast, the use of plants (e.g., extracts, fruit juices) for the synthesis of metal nanoparticles (MNPs) involves easy, simple, quick, environmentally friendly, sustainable and cost-effective processes, typically under moderate reaction conditions [9]. Plant-mediated synthesis of nanoparticles is also clinically adaptable and easily scalable for industrial production [10]. Interestingly, the secondary metabolites (e.g., polyphenols, flavonoids, tannins, terpenoids, alkaloids) contained in plant extracts often act as reducing and/or capping agents [11,12]. Depending on their morphological and physical characteristics (e.g., size, zeta potential), as well as their composition, MNPs from plants with medicinal value can exhibit improved antibacterial, antifungal and antibiofilm activities, thus constituting a very promising means of combating antimicrobial resistance [9,13,14,15,16]. 

Using different sources of metals (salts or oxide) and different plant extracts, the biological reduction method allows the synthesis of a large number of green MNPs, including silver (Ag), gold (Au), zinc oxide (ZnO), platinum (Pt), palladium (Pd), copper (Cu), iron oxide (Fe_2_O_3_ and Fe_3_O_4_), nickel oxide (NiO), magnesium oxide (MgO), titanium dioxide (TiO_2_) and indium oxide (In_2_O_3_) [8,14].

Investigation of plant systems as potential bio-factories for MNPs has received considerable attention, especially by researchers working in the field of phytonanotechnology, and pharmaceutical and clinical microbiology, as well as medicine [14,17]. Indeed, due to the surging popularity of green methods, more than 1000 research papers and reviews related to antibacterial, antifungal and antibiofilm properties of MNPs have been published to date (Pubmed and Google Scholar database). Most of the reviews published so far have mainly focused on predicting the antimicrobial mechanisms of MNPs and parameters that may influence their antibacterial, antifungal and activities, including: (i) the type (and origin) of plants used as bioreactor sources for biosynthesis, (ii) the reduction process of the metal salts (mainly silver, zinc and gold) used during the fabrication of nanoparticles, (iii) the particulate characteristics of MNPs (e.g., size, zeta potential and shape), as well as the characterization techniques allowing their determination, and (iv) the general protocols applied to evaluate the antimicrobial potential of metal-containing nanoparticles [10,13,14,17,18,19,20,21,22,23,24,25,26].

Unfortunately, it appears from these reviews that the methods used for assessing the antibacterial, antifungal and antibiofilm efficiency of MNPs are only partially elaborated in terms of standardization processes; therefore, it is hard to correlate or compare data from different studies to set out the product quality attributes and boost the development pipeline for high value antimicrobial nanoparticles. Hence, to provide an essential reference for readers, the present review briefly describes different in vitro and in vivo methods used for testing the antimicrobial activities of MNPs against planktonic bacteria, fungi and biofilms. The paper also presents tabular data (from 2010 to date) that summarize different in vitro experimental conditions used for assessing antimicrobial and antibiofilm activities of MNPs, including the major findings based on physicochemical characteristics (e.g., size and shape). We also discuss the discrepancies found in the obtained results. By doing so, this review guides scientists towards the most appropriate experimental settings for MNP evaluation and offers a useful resource for further complementary investigations. Moreover, it may pave the way for research and development of accessible and affordable drug formulations and wound-dressings containing green-synthesized metal and metal oxide nanoparticles. 

## 2. Microbial Origins and Antimicrobial Resistance of Bacterial and Fungal Infections

The human body is not sterile; it is colonized by many microorganisms that are part of the normal microflora and live as harmless commensals [27]. Bacteria living under normal conditions on the skin, nasopharynx and intestine play an important protective role, as they prevent the growth of pathogenic microorganisms in these places. The loss of the protective functions of this barrier for any reason is an important factor in the onset of infections. As the body’s condition changes, immune bacteria that have previously been weakened can become pathogenic and cause infections ranging from minor to life-threatening [28].

Pathogenic microorganisms that are present in almost every location and environment on earth (e.g., air, soil biomass, water, plants and animals) can be sources of infections [29,30]. Contemporary lifestyles (e.g., imported food, air-conditioned environments, travel abroad and visits to hospitals) also contribute to the spread of infections. In other words, infections result from ever-changing interactions between microorganisms, the human as their host, and the environment around them [31].

**Figure 1:**
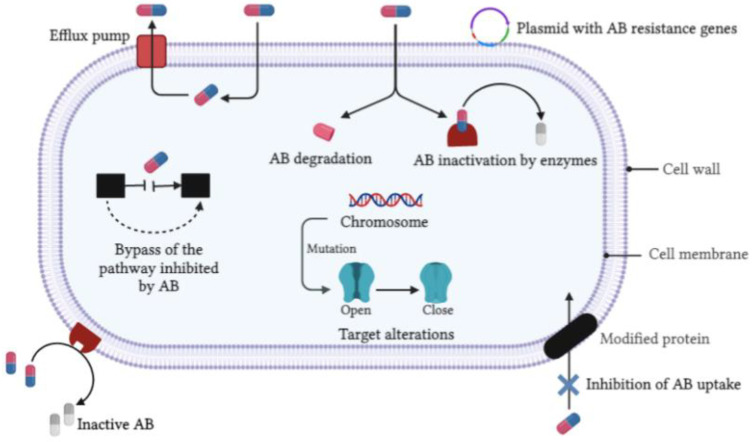
Antibiotic resistance strategies in microorganisms. Mechanisms by which bacteria (and fungi) can resist antimicrobial molecules include: (i) target alterations and modifications through genetic mutations or post-translational modifications, (ii) increased active efflux of antibiotic out of the cell through efflux pumps, a type of membrane transporter located within the microbial membrane or wall, (iii) inactivation, destruction or degradation of the antibiotic through hydrolysis or modification by different enzymes (e.g., extended-spectrum β-lactamases) that can add specific chemical moieties, such as phosphoryl groups, (iv) decreased influx of antibiotic into the bacteria, e.g., through charges in the structure of the cell wall, (v) reduced permeability of the membrane that surrounds the bacterial cell. Created with BioRender.com (accessed on 1 March 2022).

Antimicrobial agents (antibiotics and antifungals) are used to treat or prevent different types of infections caused by bacteria and fungi (as well as certain parasites). However, most antimicrobials currently face the development and spread of antimicrobial resistance [32]. The scientific literature, and recent observations, show that a growing number of bacterial infections, such as tuberculosis, salmonellosis, pneumonia and gonorrhea are becoming more difficult to treat as the antibiotics used for their treatment become ineffective [33]. These situations lead to increased treatment failures, hospitalization time, economic burden and deaths [34]. The vast majority of antimicrobial drugs currently used promote genetic instability and increased mutagenesis in bacteria and fungi [35]. The spread and accumulation of antibiotic resistance bacteria (ARB) and antibiotic resistance genes (ARGs) in the environment are one of the greatest threats to human health and represent the emergence of a difficult situation in the world [36,37]. As antibiotic resistance is occurring naturally, ARG transmission also appears to be correlated with human activities [38]. ARB and ARGs have been found in various locations, such as ground- and drinking-water [39,40,41], agricultural soil, vegetables, sewage sludge, and agricultural products, such as fish [42,43,44,45]. Therefore, tackling this scourge is becoming a global emergency with an urgent need to overcome the ability of Gram-positive/negative bacteria and fungi to resist antimicrobial drugs [46]. Figure 1 summarizes the antibiotic microbial resistance attained by different micro-organisms through various intrinsic or acquired mechanisms [47].

It should be noted that misuse of antimicrobial drugs in both human and animal health may also represent an important source of antimicrobial resistance. Antimicrobial agents are extensively used in animal husbandry and agriculture for prophylactic and therapeutic purposes [48]. In addition, antimicrobial drugs are used as growth promoters in animal feed, and to increase crop productivity. Such overuse of antimicrobial drugs outside of clinics has contributed tremendously to the rise in antimicrobial resistant strains and led to the growing need for new antimicrobial agents that can effectively treat and prevent infectious diseases worldwide. Therefore, implementing the One Health approach is proving to be a great necessity, even of the utmost urgency [49,50].

In nature, microorganisms rarely live in isolated colonies of the same species. They are characterized by “life” in biocenoses called biofilms [51]. Biofilms are communities of microbes attached to surfaces, which can be found in medical, industrial and natural settings. In fact, life in a biofilm probably represents the predominant mode of growth for microbes in most environments [52,53]. 

Mature biofilms have a number of distinct characteristics. They are surrounded by an extracellular matrix that contains polysaccharides, nucleic acids, lipids, proteins, water and ions [53,54]. This matrix provides structure and protection to the community of microorganisms. Microbes growing in a biofilm also have a characteristic architecture, generally comprised of macrocolonies (containing thousands of cells) surrounded by fluid-filled channels. Biofilm-grown microbes are notorious for their resistance to various antimicrobial agents, including clinically relevant antibiotics [55,56,57].

**Figure 2:**
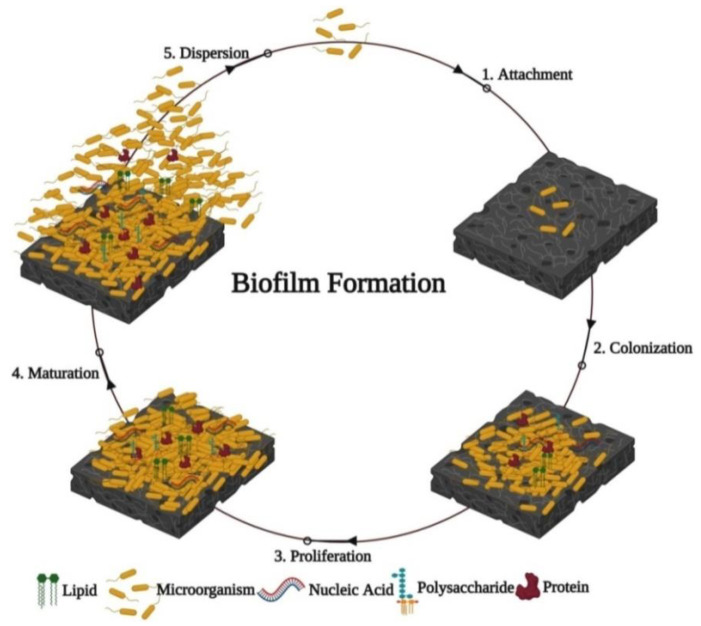
Developmental stages involved in microbial biofilm formation (From [58] with permission from Frontiers in Microbiology).

Biofilms are formed in five stages (Figure 2; [58]). The first stage (attachment) is related to the primary adhesion and adsorption of bacteria or fungi; this step is a reversible process (stage 1). In the second stage (so-called fixation or colonization), microorganisms secrete polymers that ensure strong and irreversible adhesion (stage 2). In the proliferation stage, the microorganisms attach to the epithelium, facilitate the attachment of new microbial cells, and connect the entire colony to the intercellular filler. With the accumulation of nutrients, microorganisms begin to multiply (stage 3). The following step is the stage of full maturation; the formed biofilms acquire their size and shape, and the intercellular filler protects them from external negative factors (e.g., oxygen, temperature, nutrient) (stage 4). Finally, in the last stage, bacterial spread (dispersion) occurs, during which individual bacterial cells periodically separate, creating new colonies (stage 5) [58,59,60,61]. All surfaces, including medical equipment, irrespective of their nature and the environment in which they are used, are susceptible to the colonization and infection of microorganisms and the formation of biofilms [56]. 

Overall, biofilms do not only represent a bacterial layer of mucus, but also a biological system composed of bacteria and/or fungi that are organized into a coordinated and functional biocenosis through intercellular chemical communication or quorum sensing [61]. Microorganisms in biofilms form metabolic consortia that are characterized by (i) food exchange between microbial cells (i.e., each microorganism becomes a food source for another), (ii) primitive exchange of genetic information, and (iii) resistance to phagocytosis and antibiotics. Therefore, biofilm-forming bacteria (or fungi) can be 100-fold more resistant to antimicrobial agents and disinfectants than planktonic bacteria [59]. Recurrent biofilm-induced infections are especially dangerous to people with health problems, as they can cause hospital infections. In USA, one out of thirty-one hospitalized patients have at least one healthcare-associated infection [62]. In Europe, approximately 5% of hospitalized patients suffer from hospital infections, i.e., 4.1 million people every year, including about 37 thousand deaths [63,64,65]. The prevalence of healthcare-associated infections can reach 20% in middle- and low-income countries. In addition, 4 to 56% of hospital-born babies die from health care-associated infections in the neonatal period in developing countries. This prevalence can reach 75% in south-east Asia and sub-Saharan Africa [63].

## 3. General Background on Antimicrobial and Anti-Biofilm Metallic Nanoparticles Green-Synthesized

### 3.1. Green Synthesis of MNPs

In general, there are two ways of synthesizing MNPs: the “bottom-up” and “top-down” approaches. Both methods of synthesis can be performed either in liquid (e.g., water, ethanol, hexane, toluene, ethylene glycol and others), gas, solid, or supercritical fluids, or in a vacuum [17,66,67].

**Figure 3:**
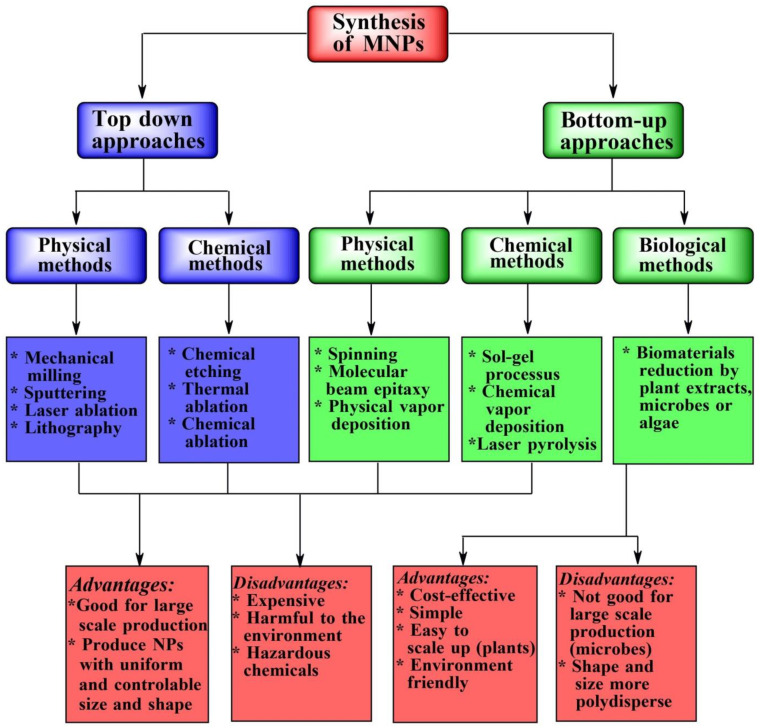
Overview of the synthesis of metal nanoparticles.

In bottom-up synthesis, atoms, molecules or clusters are grouped to form nanostructured materials [3,17,68]. A wide variety of physical and chemical methods belong to this category. The physical methods include spinning, physical vapor deposition and molecular-beam epitaxy (Figure 3) [7,69]. The chemical methods comprise sol-gel processes, laser pyrolysis, chemical vapor deposition, aerosol-based processes, atomic or molecular condensation and precipitation, plasma-spraying synthesis and supercritical fluid technology [14,66,67,70]. The green synthesis of MNPs is also a class of bottom-up methods in which reduction and oxidation are the foremost chemical reactions [2,21]. Conversely, in the top-down approach, the bulk materials are broken down gradually (or collapsed little-by-little) to yield nanoparticles. Mechanical milling, sputtering and lithography belong to physical top-down methods, while chemical top-down methods include electro-explosion and chemical etching [14,17,66,68].

In general, chemical and physical methods have the reputation of being expensive and poorly accessible to all scientists across the world, especially in developing countries [2,3]. The chemical methods are also harmful to the environment due to the use of organic solvents and highly reactive toxic chemicals and reducing agents [71]. The latter can generate unwanted by-products that can, in turn, cause potential environmental and biological risks [39,40,67,72,73]. The physical methods are limited by low production rates and high energy consumption, as well as requiring sophisticated equipment and stringent conditions [14]. 

On the other hand, the synthesis pathways using plant extracts or microorganisms are environmentally benign, non-toxic, cost-effective, simple and easily up-scalable for industrial production [23,67,74,75,76,77,78]. Using renewable materials and mild solvent media, biogenic methods of synthesis of MNPs offer additional benefits, including one-pot synthesis, no need for catalyst use, clean, yet straightforward, reaction conditions, and no toxic waste generation [14,21]. Nevertheless, synthetic microbial methods include several disadvantages, such as microorganism cultivation and the optimization of different growth parameters (e.g., nutrient medium, salt concentration, temperature, pH, incubation time, inoculum quantity, etc.) [79]. A combination of different physical factors, such as light, ultrasonic waves, microwaves, heating, etc., is also required to produce MNPs by synthetic microbial methods [36,42,80,81]. As a result, microbe-mediated synthesis of MNPs requires expertise and is also time-consuming [17]. Moreover, the intrinsic ability of microorganisms to act as reducing and capping agents and contribute to the amalgamation of metal ions into MNPs is lower than that of plant metabolites [82].

MNPs synthesized by chemical (or physical) synthesis can exhibit antibacterial activity, as described previously [83,84,85,86]. However, many researchers have reported that green-produced MNPs showed higher activity than those produced chemically [4,87]. This is in most cases the result of a kind of synergy between the intrinsic activity of the metals and of the plant metabolites themselves.

#### 3.1.1. Plant-Mediated Synthesis of Silver, Gold and Zinc Oxide Nanoparticles

The green synthesis of silver nanoparticles (AgNPs) is typically achieved by combining plant extracts and silver halides, such as silver bromide (AgBr), silver chloride (AgCl), and silver iodide (AgI) [88,89]. 

During the biosynthesis of green ZnO nanoparticles, zinc nitrate hexahydrate (Zn(NO_3_)_2_·6H_2_O) or zinc acetate (Zn(OOCCH_3_)_2_) are typically used as precursors while different plant extracts act as bio-reductants [90,91]. 

To synthesize gold nanoparticles (AuNPs) using green sources, plant extracts are simply mixed with solutions of gold salts, such as auric chloride (AuCl_3_) and chloroauric acid (HAuCl_4_) [92,93].

#### 3.1.2. Synthesis of Platinum and Palladium Nanoparticles Using Plant Extracts

Bio-fabricated PtNPs can be obtained using plant extracts as eco-friendly reducing reagents, along with sodium tetrachloroplatinate (II) (Na_2_PtCl_4_) and chloroplatinic acid hexahydrate (H_2_PtCl_6_·6H_2_O) as precursor salts [90,91]. In the process of the bio-fabrication of PdNPs, plant extracts are mixed with palladium chloride (PdCl_2_) or palladium acetate (Pd(OAc)_2_). Depending on their intrinsic properties, the phytocomponents from the plant extracts reduce the Pd ions into atoms and then produce metallic Pd nanoparticles [90,91,94].

#### 3.1.3. Biosynthesis of Other Green Metallic Nanoparticles

Nanosized CuO particles can be obtained by reducing cupric salts (e.g., cupric chloride (CuCl_2_), cupric sulfate (CuSO_4_) and cupric nitrate (Cu(NO_3_)_2_) using phytoconstituents from plant extracts [90,91]. 

The precursor salts for the green synthesis of Fe_2_O_3_ (hematite) and Fe_3_O_4_ (magnetite) nanoparticles include ferric chloride hexahydrate (FeCl_3_·6H_2_O), ferric nitrate nonahydrate (Fe(NO_3_)_3_·9H_2_O), ferric acetylacetonate (Fe(C_5_H_8_O_2_)_3_), and ferrous sulfate (FeSO_4_) [95,96]. Nickel nitrate hexahydrate (Ni(NO_3_)_2_·6H_2_O) and magnesium nitrate hexahydrate (Mg(NO_3_)_2_·6H_2_O) can be used as precursors for the synthesis of NiO and MgO nanoparticles by green process, respectively [90,91,97]. Additionally, nickel chloride (NiCl_2_) and indium nitrate (In(NO_3_)_3_·H_2_O) can be reduced to form nickel oxide nanoparticles and indium oxide (In_2_O_3_), respectively, by plant extracts [98,99]. Lead nanoparticles can be synthesized by green methods using lead oxalate (Pb(COOH)_2_) or lead nitrate (Pb(NO_3_)_2_) as metal precursors [100,101].

Concerning TiO_2_ NPs, their green synthesis can be successfully achieved by mixing plant extracts as reducing/capping materials with metatitanic acid (TiO(OH)_2_) or titanium tetraisopropoxide (Ti[OCH(CH_3_)_2_]_4_) as precursors [90,91]. Additionally, numerous bimetallic and trimetallic nanoparticles are currently synthesized by green methods. This is the case for Ag/Pt NPs, Pd/Fe_3_O_4_ NPs, Fe_3_O_4_/MgO NPs, ZnO/CoO NPs, ZnO/MnO NPs, Au/Pt/Ag NPs and Cu/Cr/Ni NPs obtained by mixture of the corresponding precursors and plant extracts [102,103,104,105].

Over the last decade, other antimicrobial hybrid derivatives, such as nanocellulose/metal and nanocellulose/oxide metal, have attracted the attention of several researchers and industries due to their environmentally friendly status and low cost compared to synthetic polymer- (e.g., polylactic-*co*-glycolic acid (PLGA), polyvinylalcohol (PVA), poly(ethylene glycol) methyl ether-*block*-poly(lactide-*co*-glycolide) (mPEG-PLGA), chitosan, gelatin) encapsulated MNPs [106,107,108,109,110,111,112,113]. Cellulose is a ubiquitous natural polymer which can be produced from a broad range of biomass. It is the key component in natural fibers and an excellent candidate for synthesis of bio-based materials due to its various physicochemical properties, including biodegradability, biocompatibility, environmental friendliness, renewability, affordability and colloidal stability [114,115]. 

Nanocellulose is defined as cellulose material that has been broken down into particles of less than 100 nm [116]. It is essentially produced through chemical or mechanical action on the plant cellulose or bacterial cellulose. Nanocellulose is classified into cellulose nanocrystals, cellulose nanofibers and bacterial nanocellulose [108,114,115]. Apart from plant materials (e.g., wood, oil palm biomass, bamboo, rice husk, coconut husk), cellulose nanocrystals and cellulose nanofibrils can also be extracted from tunicate, a type of marine invertebrate. In contrast, bacterial nanocellulose is a growing nanoproduct that can be obtained through several kinds of mutual fermentation bacteria, such as *Gluconacetobacter xylinus* [115,117].

**Figure 4:**
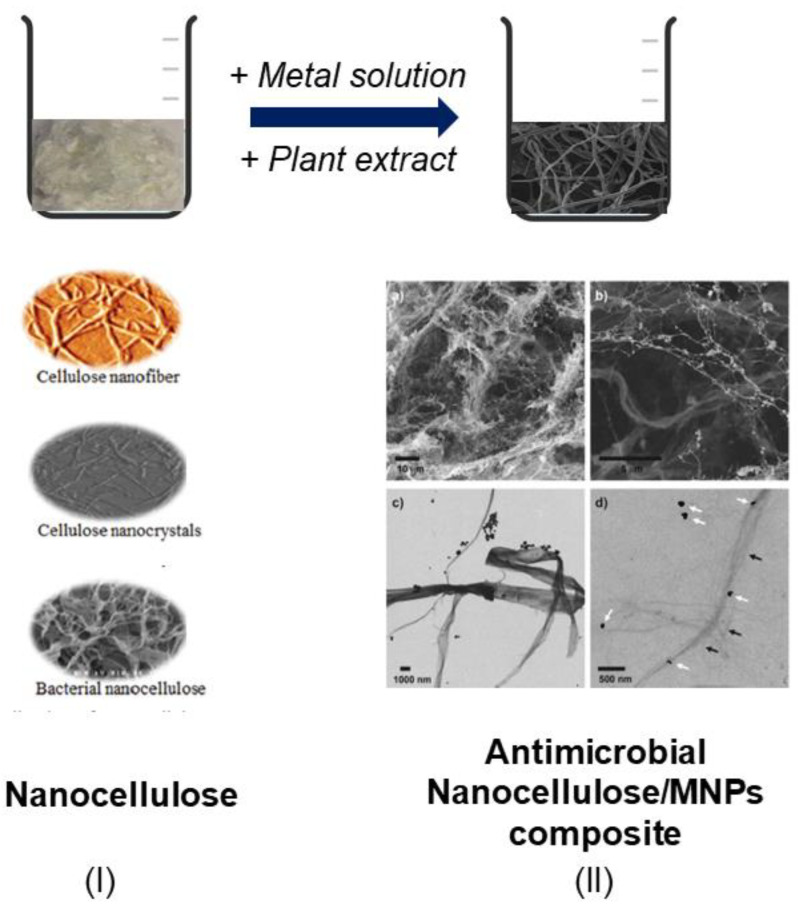
Illustrative presentation of green synthesis of nanocellulose/metal or metal oxide hybrid nanocomposites. (**I**) Different types of nanocellulose in dispersion; (**II**) Electron microscope images of MNPs in cellulose (**a** and **b** for SEM images, and **c** and **d** for TEM images, respectively): white and black arrows point to MNPs and defibrillated cellulose, respectively (Adapted from [107,118] with permission from The Royal Society of Chemistry and Scientific Reports).

Nanocellulose does not inherently elicit any antimicrobial activity. However, by functionalization with green metal/metal oxide nanoparticles (e.g., Au, Ag, Cu, CuO, MgO, ZnO, Fe_3_O_4_ and TiO_2_), it is possible to endow nanocellulose composites with antimicrobial properties (Figure 4) [118,119]. These biosynthesized metal-based antimicrobial agents may also exhibit good efficacy and resilience towards microbial resistance [114,117]. In this context, Mocanu et al. [120] recently demonstrated the impact of functionalization of bacterial nanocellulose with ZnO NPs, green-synthesized using propolis extract, on antimicrobial activity against *Bacillus subtilis* and *Candida albicans*, in comparison to ZnO NPs and the extract alone. Additionally, Razavi et al. [121] prepared antimicrobial bacterial nanocellulose film decorated with silver, copper and palladium MNPs biosynthesized using mulberry fruit (*Morus alba* L.) extract. Due to their significant activity against *Escherichia coli* and *Listeria monocytogenes*, the authors suggested that the fabricated nanocomposite film might be useful as a novel biomedical treatment to combat pathogens on food commodities.

Green-synthesized plant-based MNPs can be characterized using a wide range of physicochemical tools, such as UV-visible absorption spectroscopy (for metal surface plasmon resonance), zeta potential (for surface-charge determination), transmission electron microscopy (for size and shape analysis), X-ray diffraction (for crystallinity assessment), energy-dispersive X-ray spectroscopy (EDX) (for the determination of elemental composition on the surface), Fourier transform infrared spectroscopy (FTIR) (for the detection of organic functional groups of phytoconstituents), thermogravimetric analysis (for thermal stability), and Raman spectroscopy (for surface-capping tracking) [9,68,122,123,124].

### 3.2. Antimicrobial Activities of Green-Synthesized MNPs

Plant-mediated synthesis imparts several advantages to MNP technology for the development of alternative products against infectious diseases. Indeed, most of the green MNPs from plant-derived materials are highly effective and non-specific antimicrobial agents, showing remarkable activity against the growth of a broad spectrum of bacterial and fungal species, in both planktonic and biofilm forms, including nosocomial and multi-drug-resistant strains (Tables 1–8 and S1–S7) [11,13,14,16,125].

Given their nano-particulate features, biosynthesized MNPs provide a large surface area that increases their interactions with microorganisms, thereby resulting in strong antimicrobial activity. The antimicrobial properties of green MNPs also depend on their particle shape. Moreover, the variety of green reagents (plant extracts or phytoconstituents), metal precursors and synthetic conditions (e.g., physicochemical parameters) used have a significant effect on the antimicrobial activity of MNPs [12,13,126,127]. 

**Figure 5:**
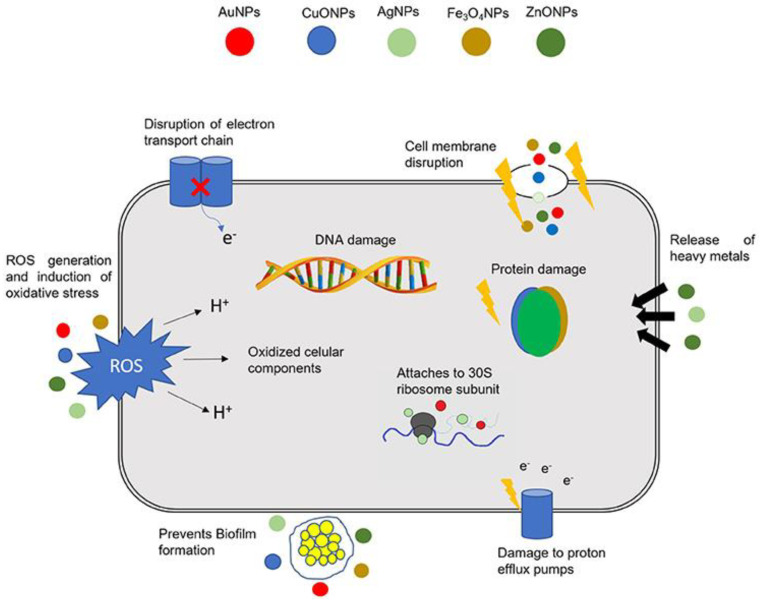
Different mechanisms of action of MNPs in microbial cells. The combination in a single nanomaterial of a multitude of cellular effects may have a tremendous impact in fighting multi-drug-resistant microorganisms (From [129] with permission from Frontiers in Microbiology).

Biofabricated MNPs may act in different ways, including: (i) destruction of the microbial cell wall, (ii) damage to efflux pump mechanisms, (iii) inhibition of deoxyribonucleic acid (DNA) replication and enzyme functions, (iv) ribosome disintegration, (v) generation of reactive oxygen species (ROS) and induction of oxidative stress; (vi) triggering of both innate and adaptive host immune responses, and (vii) inhibition of biofilm formation (Figure 5) [47,128,129]. The mechanisms of action of MNPs depend on their origin as well as their biological, physical and chemical properties [130].

The antibacterial, antifungal and antibiofilm activities of the most biosynthesized MNPs are briefly summarized in the following paragraphs. In addition, the unique physicochemical characteristics of these MNPs are briefly described since their antimicrobial activities are also attributed to their size, high surface area, zeta potential and shape. For more details, the reader can refer to more specific reviews [10,13,14,17,23,24,25,26,128,131,132,133,134].

#### 3.2.1. Silver Nanoparticles

Elemental silver has been widely used as an antimicrobial agent since ancient times [89]. To improve their antibacterial activity and reduce their toxicity, silver ions can be transformed into metallic silver nanoparticles through biological and biomimetic methods of synthesis [9]. 

Details of the antimicrobial and anti-biofouling activities of these bioactive nanoparticles are given in Table 1 and Table 2. It is notable that green AgNPs have demonstrated the ability to reduce microbial infections in skin and burn wounds and to prevent bacterial colonization on the surface of various medical devices, such as catheters and prostheses. Acting as capping agents, different multi-functional phytochemicals contribute efficiently to these antimicrobial activities [74,78,82,128]. Moreover, AgNPs can operate synergistically with standard antibiotics, such as gentamycin and streptomycin [129,135,136,137]. Hence, these combinations can be used effectively against antibiotic-resistant pathogens. Additionally, the antifungal activity of AgNPs has been extensively studied and demonstrated in the literature [138,139].

In the context of the fight against antibiotic resistance, green-synthesized AgNPs may be used as vehicles to transport oligonucleotide-based antimicrobials [140,141,142]. AgNPs can also be incorporated in hydrogel beds, cyclodextrins, and lipid-based formulations (e.g., liposomes), creating the potential for controlled release and targeted delivery [143,144,145]. Interestingly, AgNPs are found in a number of commercially available products, including medical devices for healthcare settings, dietary and health supplements, potential additives to animal feed, food packing materials and kitchen appliances [146,147,148].

**Table 1:** Green silver nanoparticles exhibiting antimicrobial activity.

Plant Type	Part Used	Operative Conditions for Synthesis	NP Characteristics (Shape and Size)	Microbiological Analyzes (Operative Conditions)			Refs.
				Methods, Incubation Temperature, Incubation Time, pH, Inoculum Density, Positive Control	Tested Bacteria and Fungi	MIC, ZOI or PI *	
*Lysiloma acapulcensis*	Roots	Silver nitrate 1 mM/plant extract 2% (1:1 *v*/*v*) Room temperature 2 min pH NM	Spherical 1.2–62 nm	Diffusion 37 °C 24 h pH NM Inoculum NM No control	*E. coli* ATCC 25922 *P. aeruginosa* ATCC 27853 *S. aureus* ATCC 49476	18 15 16 mm	[87]
*Perilla frutescens*	Leaves	Silver nitrate 2 mM/plant extract 10% (9:1 *v*/*v*) 50 °C 2 h pH NM	Spherical, rhombic, triangle, and rod 25.7 nm	Diffusion 37 °C 24 h pH NM Inoculum NM Streptomycin **	*E. coli* *B. substilis* *S. aureus*	14 12 10 mm	[149]
*Ocimum canum*	Leaves	Silver nitrate 1 mM/plant extract 10% (9:1 *v*/*v*) 80 °C 15 min pH NM	Spherical 6.1–32.1 nm	Diffusion 28 °C 24 h pH NM Inoculum NM No control	*E. coli*	25 mm	[150]
*Piper longum*	Catkin	Silver nitrate 1 mM/plant extract 10% (5:1 *v*/*v*) Room temperature 2 h pH NM	Spherical 10–42 nm	Diffusion 37 °C 24 h pH NM Inoculum NM No control	*B. cereus* MTCC 1272 *E. coli* MTCC 1687 *K. pneumoniae* MTCC 530 *Proteus mirabilis* MTCC 425 *P. aeruginosa* MTCC 1688 *S. typhi* MTCC 531 *S. aureus* MTCC 96	12 13 14 15 11 12 11 mm	[139]
*Diospyros malabarica*	Fruits	Silver nitrate 1 mM/plant extract 20% (9:1 *v*/*v*) 25 °C 1 h pH NM	Spherical 17.4 nm	Diffusion 37 °C 24 h pH NM Inoculum NM Streptomycin 10 µg Tetracycline 30 µg Chloramphenicol 30 µg	*E. coli* *S. aureus*	13 12 mm	[151]
*Pyrenacantha grandiflora*	Tuber	Silver nitrate 1 mM/plant extract 0.1% (1:1 *v*/*v*) Room temperature Incubation time NM pH NM	Spherical 3–25 nm	Dilution 37 °C 24 h pH NM Inoculum NM No control	*E. coli* *K. pneumoniae* *S. aureus*	0.8 0.8 0.8 µg/mL	[152]
*Carissa carandas*	Leaves	Silver nitrate 1 mM/plant extract 10% (9:1 *v*/*v*) 60 °C 1 h pH 7.2	Spherical 30 nm	Diffusion 37 °C 24 h pH NM 1 × 10^8^ CFU/mL No control	*S. typhi* *Enterococcus faecalis* *Shigella flexneri* *Citrobacter* spp. *Gonococci* spp.	12 16 24 14 21 mm	[153]
*Solanum tricobatum*	Leaves	Silver nitrate 1 mM/plant extract 1.5% (1:10 *v*/*v*) 37 °C 24–48 h pH NM	Irregular 26.5 nm	Diffusion 35 °C 18 h pH NM Inoculum NM No control	*S. aureus* *P. aeruginosa* *E. coli* *K. pneumoniae*	30 12 14 18 mm	[154]
*Melissa officinalis*	Leaves	Silver nitrate 5 mM/plant extract 25% (1:2 *v*/*v*) 25 °C 1 h pH NM	Spherical 12 nm	Diffusion 36 °C 24 h pH NM Inoculum NM No control	*E. coli* *S. aureus*	12 13 mm	[155]
*Piper betle*	Leaves	Silver nitrate 1 mM/plant extract 10% (10:1 *v*/*v*) Room temperature 24 h pH NM	Spherical Size NM	Diffusion 30 °C 24 h pH NM Inoculum NM No control	*B. subtilis* *Klebsiella planticola*	14 13 mm	[156]
*Rosa canina*	Fruits	Silver nitrate 1 mM/plant extract% NM (5:1 *v*/*v*) 85 °C Incubation time NM pH NM	Spherical 13–21 nm	Dilution 37 °C 24 h pH NM 2.4 × 10^7^ CFU/mL No control	*Bacillus cereus* *E. coli* ATCC 10536 *S. aureus* ATCC 6538 *P. aeruginosa* ATCC 9027 *Enterococcus hirae* ATCC 10541 *Legionella pneumophila* ATCC 33152	32 256 256 128 256 16 µg/mL	[157]
				Dilution 25 °C 48 h pH NM 2.4 × 10^7^ CFU/mL No control	*C. albicans*	128 μg/mL	
*Fagonia indica*	Callus	Silver nitrate 4 mM/plant extract 2% (1:1 *v*/*v*) 20 °C 3 h pH NM	Cubic Size NM	Diffusion 37 °C 24 h pH NM 1 × 10^8^ CFU/mL Ciprofloxacin **	*E. coli* ATCC 23716 *S. typhi* ATCC 35664 *Shigella sonnei* ATCC 29930 *Citrobacteramalonaticus* ATCC 25405	12 13 13 12 mm	[158]
*Barleria longiflora*	Leaves	Silver nitrate 1 mM/plant extract 20% (9:1 *v*/*v*) Temperature NM Incubation time NM pH NM	Spherical 2.4 ± 0.5 nm	Diffusion 37 °C 24 h pH NM Inoculum NM Chloramphenicol **	*Enterococcus* spp. *Streptococcus* spp. *Bacillus megaterium* *Pseudomonas putida* *P. aeruginosa* *S. aureus*	18 16 15 17 18 14.5 mm	[159]
*Ipomoea batatas*	Outer peels	Silver nitrate 1 mM/plant extract 40% (10:1 *v*/*v*) 55 °C 24 h pH NM	Shape NM Size NM	Diffusion Temperature NM Incubation time NM pH NM Inoculum NM No control	*Enterococcus feacium* DB 01 *S. enteritica* KCCM 11806 *Listeria monocytogenes* ATCC 19111 *B. cereus* KCTC 3624 *S. aureus* ATCC 13565	10 11 11 11 0 mm	[160]
*Oedera genistifolia*	Leaves	Silver nitrate 0.1 mM/plant extract 20% (9:1 *v*/*v*) Room temperature 1 h pH NM	Spherical 10–60 nm	Dilution 37 °C 24 h pH NM 1 × 10^8^ CFU/mL Ciprofloxacin **	*Enterobacter cloacae* ATCC 13047 *Listeria ivanovic* ATCC 19119 *Streptococcus uberis* ATCC 700407 *S. aureus* ATCC 29213 *Vibrio* spp. *Mycobacterium smergatis* ATCC 19420	0.5 1 0.5 0.5 0.25 0.25 mg/mL	[161]
*Derris trifoliate*	Seeds	Silver nitrate 1 mM/plant extract 20% (20:1 *v*/*v*) Temperature NM Incubation time NM pH NM	Spherical 16 ± 5 nm	Diffusion NM 24 h pH NM Inoculum NM No control	*E. coli* MTCC 723 *K. pneumoniae* MTCC 109 *P. aeruginosa* MTCC 424 *S. aureus* MTCC 96	19.5 20 36 0 mm	[162]
*Ficus krishnae*	Stem bark	Silver nitrate 1 mM/plant extract 5% (1:1 *v*/*v*) 37 °C 24 h pH NM	Spherical 160–260 nm	Diffusion 37 °C 24 h pH NM Inoculum NM No control	*E. coli* MTCC 45 *S. typhimurium* MTCC 98 *S. aureus* ATCC 29122	18 13 12 mm	[163]
*Psidium guajava*	Leaves	Silver nitrate 10 mM/plant extract 2% (10:1 *v*/*v*) 70 °C 1 h pH NM	Spherical 96 ± 4 nm	Diffusion 37 ± 2 °C 48 h pH NM 1–2 × 10^5^ CFU/mL No control	*C. albicans* ATCC 10231	14.2 mm	[164]
*Citrus limon*	Leaves	Silver nitrate 2 mM/plant extract 20% (9:1 *v*/*v*) 25 °C 1 h pH NM	Spherical 8–15 nm	Diffusion Temperature NM 18–24 h pH NM Inoculum size NM No control	*Fusarium oxysporium* *Alternaria brassicicola*	15 10 mm	[165]
*Chaenomeles sinensis*	Fruits	Silver nitrate 1 mM/plant extract 10% (ratio NM) 80 °C 65 min pH NM	Spherical 5–20 nm	Diffusion 37 °C 24 h pH NM Inoculum NM Neomycin **	*E. coli* *S. aureus*	14 10 mm	[166]
*Persicaria odorata*	Leaves	Silver nitrate 1 mM/plant extract 2% (10:1 *v*/*v*) 25 °C 24 h pH NM	Spherical 11 ± 3 nm	Dilution 37 °C 18 h pH NM 1 × 10^6^ CFU/mL No control	*S. epidermidis* ATCC 12228 MRSA ATCC 43300	3-LR *** 6-LR	[167]
*Citrus reticulata*	Peels	Silver nitrate 1 mM/plant extract 21.8% (1:1 *v*/*v*) Temperature NM Incubation time NM pH NM	Spherical 45 nm	Dilution 37 °C 48 h pH NM 1 × 10^5^ CFU/mL No control	*Desulfovibrio* spp.	3-LR	[168]
*Cuccuma longa*	Rhizome	Silver nitrate 1 mM/plant extract 6.8% (4:1 *v*/*v*) Temperature NM 24 h pH NM	Spherical 18 nm	Dilution 37 °C 24 h pH NM 1 × 10^8^–10^9^ CFU/mL No control	*E. coli* *Listeria monocytogenes*	4-LR 4-LR	[169]

* MIC = minimal inhibition concentration; ZOI = zone of inhibition; PI = percentage of inhibition. ** The quantity or concentration is not mentioned. *** LR = log reduction. A 1-log, 2-log, 3-log, 4-log, 5-log and 6-log reduction in living microorganisms or CFUs by MNPs corresponds to their inactivation or inhibition of 90, 99, 99.9, 99.99, 99.999 and 99.9999%, respectively. NM = not mentioned.

**Table 2:** Green silver nanoparticles with antibiofilm activity.

Plant Type	Part Used	Operative Conditions for Synthesis	NP Characteristics (Shape and Size)	Microbiological Analyzes (Operative Conditions)			Refs.
				Methods, Incubation Temperature, Incubation Time, pH, Inoculum Density, Positive Control	Tested Bacteria and Fungi	MIC, ZOI or PI *	
*Punica granatum*	Peel	Silver nitrate */plant extract 5% (ratioNM) Temperature NM Incubation time NM pH NM	Spherical 32–85 nm	Microtiter plate 37 °C 24 h pH NM 1.5 × 10^8^ CFU/mL No control	*P. aeruginosa* ATCC 10662	89.6%	[170]
*Artemisia scoporia*	NM	Silver nitrate 1000 mM/plant extract 10% (20:1 *v*/*v*) Temperature NM 24 h pH NM	Spherical 10–80 nm	Microtiter plate 37 °C 24 h pH NM 1.5 × 10^8^ CFU/mL No control	*S. aureus*	6.25 µg/mL	[171]
*Prosopis juliflora*	Leaves	Silver nitrate 1 mM/plant extract 10% (9.5:0.5 *v*/*v*) 25 °C 40 min pH NM	Spherical 10–20 nm	Congo red agar plate 37 °C 24–48 h pH NM Inoculum size NM No control	*B. substilis* *P. aeruginosa*	NM NM	[172]
*Malva sylvestris*	Leaves	Silver nitrate 1 mM/plant extract 20% (10:0.4 *v*/*v*) Temperature NM Incubation time NM pH NM	Spherical 10–50 nm	Dilution 37 °C 40 h pH NM 1 × 10^8^ CFU/mL No control	*P. aeruginosa* 48 *P. aeruginosa* B 52	62.5 62.5 μg/mL	[173]
*Cannabis sativa*	Stem	Silver nitrate 1 mM/plant extract 10% (1:1 *v*/*v*) Temperature NM Incubation time NM pH NM	Spherical 20–40 nm	Microtriter plate 37 °C 24 h pH NM 2–5 × 10^6^ CFU/mL No control	*P. aeruginosa* PA01 *E. coli* UTI89 *S. epidermidis*	6.25 12.5 50 µg/mL	[174]
*Rhodiola rosea*	Rhizome	Silver nitrate 5 mM/plant extract 10% (2:8 *v*/*v*) 90 °C 10 min pH NM	Spherical 15–30 nm	Dilution 37 °C 24 h pH NM 1–2 × 10^6^ CFU/mLNo control	*P. aeruginosa* *E. coli*	50 100 µg/mL	[131]
*Flacourtia indica*	Leaves	Silver nitrate 1 mM/plant extract 10% (1:1 *v*/*v*) 70 °C Incubation time NM pH NM	Spherical 45.9–64.9 nm	Congo red 37 °C 24 h pH NM Inoculum size NM No control	*Acinetobacter baumannii* SAB5 *P. aeruginosa* ETPS11 *K. pneumoniae* SKP7 *P. mirabilis* PPM8 *E. coli* ETEC12	80 80 80 80 80 μg/mL	[116]
*Dodonaea viscosa*	Leaves	Silver nitrate 1 mM/plant extract 10% (ratio NM) Temperature NM 18 h pH NM	Spherical 40–55 nm	Crystal violet assay 37 °C 24 h pH NM 1 × 10^7^ CFU/mL No control	*C. albicans* *Candida tropicalis* *Candida glabrata*	80 80 80%	[175]
*Piper betle*	Leaves	Silver nitrate 1 mM/plant extract 5% (19:1 *v*/*v*) 37 °C 6 h pH NM	Spherical 156.4 nm	Microtiter plate 18 °C Temperature NM pH NM Inoculum size NM No control	*Serratia marcescens* *Proteus mirabilis*	71 69%	[176]
*Pedalium murex*	Seed	Silver nitrate 1 mM/plant extract 5% (49:1 *v*/*v*) Temperature NM 20 min pH NM	Hexagonal 20–30 nm	Microtiter plate 37 °C 24 h pH NM Inoculum size NM No control	*Enterococcus faecalis* *S. aureus* *Shigella sonnei* *P. aeruginosa*	64 62 50 54%	[177]
*Solanum nigrum*	Fruit	Silver nitrate 1 mM/plant extract 10% (50:1 *v*/*v*) Temperature NM10 min pH NM	Spherical 10–20 nm	Microtiter plate 37 °C 24 h pH 7.2 1 × 10^8^ CFU/mL No control	*Bacillus pumulis* *Enterococcus faecalis* *Proteus vulgaris* *Vibrio parahaemolyticus*	92 84 74 62%	[178]
*Eucalyptus globulus*	Leaves	Silver nitrate 1 mM/plant extract 20% (4:1 *v*/*v*) 60 °C 30 min pH 8	Spherical 18 nm	Microtitre plate 37 °C 24 h pH NM 1 × 10^7^ CFU/mL No control	*P. aeruginosa* *S. aureus*	95 90%	[179]
*Allophylus cobbe*	Leaves	Silver nitrate 5 mM/plant extract 20% (10:1 *v*/*v*) 60 °C 6 h pH 8	Spherical 2–10 nm	Microtitre plate 37 °C 4 h pH NM 1 × 10^6^ CFU/mL No control	*P. aeruginosa* *Shigella flexneri* *S. aureus* *Streptococcus pneumoniae*	90 90 60 75%	[180]
*Cinnamomum aromaticum*	NM	NM NM NM NM	Spherical 15–50 nm	Dilution Temperature NM 24 h pH NM 1 × 10^6^ CFU/mL No control	*Streptococcus agalactiae* ATCC 27956	4 μg/mL	[181]
*Prunica granatum*	Leaves	Silver nitrate 1.5 mM/plant extract 5% (1:1 *v*/*v*) 31.4 °C 20 min pH NM	Spherical 37.5 nm	Congo red agar 37 °C 24 h pH NM Inoculum size NM No control	*P. aeruginosa* *S. aureus*	45 28%	[182]
*Terminalia catappa*	Leaves	Silver nitrate 10 mM/plant extract 5% (1:1 *v*/*v*) 30 °C 20 min pH NM	Spherical 3.5–10.1 nm	Microtiter plate 37 °C 24 h pH NM 1 × 10^7^ CFU/mL No control	*P. aeruginosa* *S. aureus*	73.7 69.6%	[183]
				Microtiter plate 37 °C 24 h pH NM 5 × 10^6^ CFU/mL No control	*C. albicans*	63.6%	

* MIC = minimal inhibition concentration; ZOI = zone of inhibition; PI = percentage of inhibition. The quantity or concentration is not mentioned. NM = not mentioned.

#### 3.2.2. Gold Nanoparticles

Due to their outstanding antimicrobial and antibiofilm properties, biosynthesized AuNPs are considered one of the most attractive MNPs. Indeed, as shown in Table 3, green AuNPs significantly inhibit the growth of medically important pathogenic bacteria and fungi.

The antimicrobial and antibiofilm properties of AuNPs extend their application to the cosmetic and agricultural fields [184,185]. The applications of antibacterial AuNPs are increasing day-by-day in environmental scenarios, as well as in the impregnation of filters [186,187]. Additionally, AuNPs can bind covalently and non-covalently with secondary coating molecules (e.g., PEG), or other materials, through surface modification. This is to minimize non-specific targeting on other tissues and for the purpose of imaging.

It is of note that previous studies have demonstrated that AuNPs obtained by chemical synthesis are generally not bactericidal, or only weakly so at high concentrations [188,189,190,191]. The reason why these AuNPs may appear to be bactericidal may, inter alia, be due to the bactericidal activity of co-existing organic complexes of Au (I and III) ions that are in the surrounding environment of the AuNPs, and which are not completely removed during centrifugation. The bactericidal properties of AuNPs can be tailored during green synthesis by considering the exposure time required for reduction of Au (III), as well as the speed and number of rounds of centrifugation needed to remove gold ions in excess. This may avoid the observed discrepancies in the antibacterial effects of green AuNPs.

**Table 3:** Green gold nanoparticles exhibiting antibacterial and antifungal activities.

Plant Type	Part Used	Operative Conditions for Synthesis	NP Characteristics (Shape and Size)	Microbiological Analyzes (Operative Conditions)			Refs.
				Methods, Incubation Temperature, Incubation Time, pH, Inoculum Density, Positive Control	Tested Bacteria and Fungi	MIC, DOI or PI *	
*Piper betle*	Leaves	Gold (III) chloride 1 mM/plant extract 1% (10:1 *v*/*v*) 30 °C 24 h pH NM	Spherical Size NM	Diffusion 30 °C 24 h pH NM Inoculum size NM No control	*B. subtilis* *Klebsiella planticola*	13 14 mm	[156]
*Musa acuminata*	Flowers	Chloroauric acid 1 mM/plant extract 25% (9:1 *v*/*v*) Room temperature 30 min pH NM	Spherical 10–16 nm	Diffusion Temperature NM 24 h pH NMInoculum size NM Streptomycin 10 μg	*S. aureus* *Enterococcus faecalis* *E. coli* *S. typhi* *P. aeruginosa* *Proteus mirabilis*	0 11 7 9 9 8 mm	[192]
*Zingiber officinale*	Roots	Chloroauric acid 1 mM/plant extract 1% (2:1 *v*/*v*) 50 °C 24 h pH NM	Hexagonal 10–20 nm	Diffusion 37 °C 24 h pH NM 1.5 × 10^8^ CFU/mL No control	*S. aureus* *E. coli* *K. pneumoniae*	14 11 17 mm	[193]
*Areca catechu*	Nut	Chloroauric acid 1 mM/plant extract 5% (10:1 *v*/*v*) 80 °C 1 h pH NM	Spherical 14 nm	Diffusion 37 °C 24 h pH NM Inoculum size NM No control	*S. aureus* *E. coli*	12 14 mm	[194]
*Momordica cochinchinensis*	Rhizome	Chloroauric acid 0.01 mM/plant extract 10% (2:1 *v*/*v*) Room temperature 24 h pH NM	Spherical 16 ± 2 nm	Diffusion 37 ± 1 °C 24 h pH NM 1 × 10^8^ CFU/mL Streptomycin 100 µg/mL	*S. aureus* *E. coli* *B. subtilis* *P. aeruginosa*	19 22 19 24 mm	[195]
*Plumeria alba*	Flowers	Chloroauric acid 1 mM/plant extract 5% (5:2 *v*/*v*) Room temperature 4 h pH NM	Spherical 15 nm	Diffusion 37 °C 24 h pH NM Inoculum size NM No control	*E. coli*	16 mm	[196]
*Coleus forskohlii*	Root	Chloroauric acid 0.1 mM/plant extract 8% (1:1 *v*/*v*) Room temperature 2 h pH 13	Spherical 5 nm	Diffusion 37 °C 24–48 h pH NM Inoculum size NM Tetracyclin 30 μg/mL	*Proteus vulgaris* *Micrococcus luteus*	18 14 mm	[197]
*Euphorbia wallichii*	Leaves	Chloroauric acid 1 mM/plant extract 5% (1:10 *v*/*v*) 30 °C 24 h pH NM	Hexagonal 8 nm	Dilution 34 °C 24 h pH NM Inoculum size NM Streptomycin **	*E. coli* *S. aureus* *Bacillus pumilus* *P. aeruginosa* *K. pneumonia*	21 15 21 17 17 mm	[198]
*Coleus aromaticus*	Leaves	Chloroauric acid 1 mM/plant extract 30% (1:1 *v*/*v*) 100 °C 30 min pH NM	Triangular 20 nm	Diffusion 37 °C 24 h pH NM 1 × 10^8^ CFU/mL No control	*S. epidermidis* *E. coli*	22 27 mm	[199]
*Origanum vulgare*	Leaves	Chloroauric acid 1 mM/plant extract 10% (10:1 *v*/*v*) 85 °C 1 min pH NM	Spherical 52 nm	Diffusion 37 °C 24 h pH NM 1 × 10^8^ CFU/mL No control	*Salmonella enteritidis* ATCC 13076 *E. coli* ATCC 25922 *Listeria monocytogenes* ATCC 13932 *S. aureus* ATCC 6538 *C. albicans* ATCC 10231	10 8 10 21 28 mm	[200]
*Perilla frutescens*	Leaves	Chloroauric acid 1 mM/plant extract 10% (1:10 *v*/*v*) 30 °C 10 min pH NM	Triangular 50 nm	Diffusion 37 °C 24 h pH NM Inoculum size NM No control	*E. coli* *B. subtilis* *S. aureus*	14 10 10 mm	[201]
*Parkia roxburghii*	Leaves	Chloroauric acid 1 mM/plant extract 1% (1:1 *v*/*v*) 30 °C 12 h pH NM	Quasi-spherical 5–25 nm	Diffusion 37 °C 24 h pH NM Inoculum size NM No control	*S. aureus* *E. coli*	NM NM	[202]
*Cibotium barometz*	Roots	Chloroauric acid 1 mM/plant extract 5% (20:1 *v*/*v*) 80 °C Incubation time NM pH NM	Spherical 23 nm	Diffusion 37 °C 24 h pH NM Inoculum size NM Neomycin 30 µg	*E. coli* ATCC 10798 *S. aureus* ATCC 6538 *Salmonella enterica* ATCC 13076 *P. aeruginosa* ATCC 10145	16 17 13 12 mm	[203]
*Mangifera indica*	Seed	Chloroauric acid 1 mM/plant extract 10% (6:4 *v*/*v*) 80 °C 1 h pH NM	Spherical 50 nm	Diffusion 37 °C 24–48 h pH NM 1 × 10^8^ CFU/mL No control	*E. coli* *S. aureus*	25 25 μg/mL	[204]
*Rhodiola rosea*	Rhizome	Chloroauric acid 1 mM/plant extract 10% (10:1 *v*/*v*) 80 °C 30 min pH NM	Spherical 13–17 nm	Diffusion 37 °C 24 h pH NM Inoculum size NM No control	*S. aureus* ATCC 29213 *E. coli* ATCC 25922	15 12 mm	[131]
*Amomum villosum*	Fruit	Chloroauric acid 1 mM/plant extract 10% (10:1 *v*/*v*) 100 °C 60 min pH NM	Spherical 5–10 nm	Diffusion 37 °C 24 h pH NM Inoculum size NM Neomycin **	*S. aureus* *E. coli*	15 15 mm	[205]
*Syzygium cumini*	Seed	Chloroauric acid 1 mM/plant extract 2% (1:2 *v*/*v*) 90 °C 1 h pH NM	Spherical 13–30 nm	Diffusion 32 °C 24 h pH NM1 × 10^4^ CFU/mL Gentamicin **	*E. coli* *B. subtilis* *S. aureus*	30 33 29 mm	[206]
*Hovenia dulcis*	Fruit	Chloroauric acid 1 mM/plant extract 2.5% (5:1 *v*/*v*) 80 °C 10 min pH NM	Spherical and hexagonal 20 nm	Diffusion 37 °C 24 h pH NM Inoculum size NM Ciprofloxacin 100 µg	*E. coli* *S. aureus*	18 19 mm	[207]
*Inonotus obliquus*	Leaves	Chloroauric acid 1 mM/plant extract 5% (19:1 *v*/*v*) Room temperature 30 min pH NM	Spherical 23 nm	Diffusion 37 °C 24 h pH NM Inoculum size NM No control	*B. subtilis* *S. aureus* *E. coli*	12 16 14 mm	[208]
*Gloriosa superba*	Leaves	Chloroauric acid 1 mM/plant extract 10% (20:1 *v*/*v*) 50–60 °C 10 min pH 5.2	Triangular and spherical 20 nm	Diffusion 37 °C 24 h pH NM 1.5 × 10^8^ CFU/mL Ampicillin 30 µg	*B. subtilis* ATCC 6633 *E. coli* MTCC 40	6.3 5.3 mm	[209]

* MIC = minimal inhibition concentration; ZOI = zone of inhibition; PI = percentage of inhibition. ** The quantity or concentration is not mentioned; NM = not mentioned.

#### 3.2.3. Zinc Oxide Nanoparticles

Zinc is an essential trace element known to have antimicrobial properties against a broad spectrum of microorganisms [210]. The metal zinc is widely present in nature as zinc oxide (ZnO), which is largely used as an antibacterial, antifungal and antibiofilm agent in drug and cosmetic products (e.g., antiseptic powders, shampoos and ointments) [210,211]. To reduce the toxicity of ZnO produced synthetically, a green and eco-friendly synthetic route has been developed to prepare biocompatible ZnO nanoparticles. Table 4 summarizes some green sources alongside the physicochemical characteristics (e.g., size, shape), and the antimicrobial activity of the developed ZnO nanoparticles.

#### 3.2.4. Platinum Nanoparticles

Platinum is an inert, biocompatible, nonporous and hypoallergenic metal that is used as an antimicrobial agent for catheters, hip and knee replacement implants, surgical and cardiac stents, implantable cardiovascular defibrillators, etc. [221,222,223]. This metallic chemical element does not corrode into harmful or potentially allergenic substances when kept with soft tissue or bone. Additionally, platinum is not prone to bacterial adhesion and infection since it forms a uniformly smooth surface when plated onto another material, thereby significantly benefiting biomedical applications [221,222,223]. According to the literature, green-synthesized platinum nanoparticles (PtNPs) are found to improve the antibacterial, antifungal and antibiofilm activity of Pt ions (see Table 5).

#### 3.2.5. Palladium Nanoparticles

Palladium (Pd) is a noble metal widely used as a platinum substitute due to its similar attributes and functionalities [203]. Indeed, Pd’s inherent biocompatibility, hypoallergenicity, chemical inertness, non-porosity and antimicrobial potential make it valuable for the manufacture of medical devices, thereby preventing corrosion and disease infections. Biogenic PdNPs also show outstanding antimicrobial properties (see Table 6); for this reason, they are widely applied in dental and surgical implants as well as prostheses [221]. 

#### 3.2.6. Other Green Metallic Nanoparticles

Green CuO, Fe_2_O_3_, Fe_3_O_4_, NiO, MgO, MnO, Mn_5_O_8_ and TiO_2_ nanoparticles are also found to be effective against bacteria and fungi (see Table 7 and Table 8) [25,250]. The inorganic nanoparticles conjugated with nanocellulose-based antimicrobial materials may have huge potential applications in the area of drug delivery, (bio)pharmaceuticals, (dermo)cosmetology, wound dressing, tissue engineering, food packaging, water treatment, air filtration, coating, mask cartridges, etc. [107,115,119]. Nevertheless, microbiological studies remain scarce for this type of nanoparticle, and further investigations are needed. 

**Figure 6:**
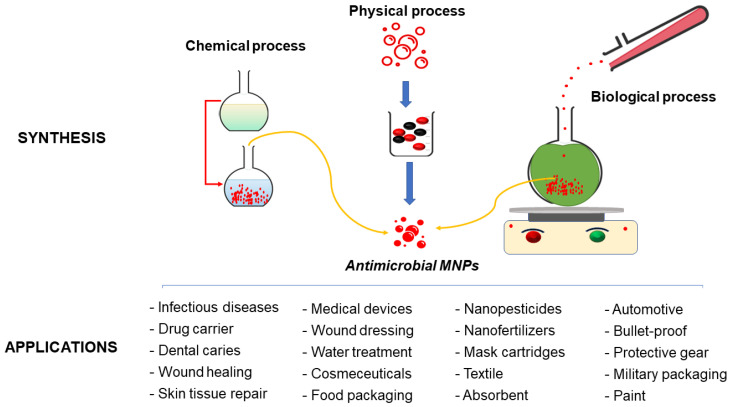
Overview of the different applications of antimicrobial MNPs.

Considering the above, it is clear that green MNPs are promising as antimicrobials. They can solve many problems in medicine, human health, nanomedicine and other fields (Figure 6).

## 4. Methods for Testing Antibacterial and Antifungal Activities of Green MNPs

Planktonic bacterial and fungal cells are free-living or free-floating bacteria and fungi that are responsible for several infectious diseases. The biological properties of green-synthesized MNPs against bacteria and fungi are well-documented in the literature [285,286,287,288,289,290,291,292]. To assess these antimicrobial potentials, different physical and analytical characterization techniques can be used [293,294]. Physical characterization techniques (e.g., atomic force microscopy, fluorescence spectroscopy, ultra-microtome-based transmission electron microscopy, inductively coupled plasma mass spectroscopy) have been developed to ascertain different information related to the interactions between MNPs and microorganisms [295]. They also provide information about the microbial killing mechanisms of green MNPs [296,297]. However, these physical techniques are beyond the scope of the present review.

Analytical techniques (e.g., colony-forming-unit (CFU) assay, live-dead staining assay, disk diffusion assay, minimum inhibitory concentration assay, capillary electrophoresis, enzyme-linked immunoassay, and polymerase chain reaction) constitute the most common methods used for testing the antimicrobial activity of MNPs [298]. As shown in Table 1, Table 2, Table 3, Table 4, Table 5, Table 6, Table 7 and Table 8 and S1–S7, diffusion and dilution susceptibility testing represent the main analytical techniques for the assessment of the antimicrobial activity of MNPs [199,247,299,300,301,302]. Both these analytical techniques are described below.

### 4.1. Analytical Techniques: Diffusion and Dilution Susceptibility Testing Methods

In diffusion susceptibility testing methods (also known as disk diffusion assay), green-synthesized MNPs impregnated in discs diffuse in an agar medium (which contains the tested bacterium) and surround the discs. This diffusion (or spread) phenomenon leads to inhibition of the growth of bacteria and fungi in an area around the discs [303]. The diameter of this inhibition zone is determined by the distance that the inhibitory concentration of the green-synthesized MNPs may travel before a certain microbial density [304]. Antibiograms and antifungiograms obtained by this method are widely used because of their simplicity, low-cost, ability to test a large number of microorganisms and antimicrobial agents, and readily interpretable results [305]. However, this method is not suitable for determining the minimum inhibitory concentration (MIC), i.e., the minimum concentration of biofabricated MNPs that inhibit the visible growth of microorganisms after a given time (18–24 h) of incubation at a predefined temperature [306]. To determine the MIC by diffusion methods, some researchers impregnate sterile paper discs with a volume of MNP solutions, while others cut wells of a certain diameter (generally 6 mm) in fungi- or bacteria-containing plates using a sterile corkborer, and then add a volume of nanoparticle solutions into each well [307,308,309].

In dilution susceptibility testing methods (so-called dilution methods), the evaluation of antimicrobial activity of eco-friendly MNPs can be performed using either agar culture medium (e.g., Mueller–Hinton agar (MHA)) (agar dilution method) or liquid culture medium (e.g., Mueller–Hinton broth (MHB)) (broth or liquid dilution method) [310,311,312,313]. Dilution methods are more suitable for determining the MIC than diffusion methods, as they provide a better quantitative estimate of the antibacterial and antifungal activity of MNPs [314,315]. The agar dilution method requires mixing of different concentrations of nanoparticle solutions in the molten MHA medium. After pouring and solidifying the obtained mixture into Petri dishes on a level surface to a specific agar depth, a quantity of standardized bacterial or fungal suspension is inoculated on the agar surface by multiple streaks [316]. The agar plates are then incubated according to validated procedures and/or practice guidelines of accredited organisms, such as the European Committee on Antimicrobial Susceptibility Testing (EUCAST) and the Clinical and Laboratory Standards Institute (CLSI) [317,318].

On the other hand, the MHB dilution method can be performed in tubes (macromethod or macrodilution method) or in microtiter plates (micromethod or microdilution method) [319]. The broth microdilution method is preferred over the broth macrodilution method for the development of antibiograms because it is easy to handle, cost-effective and can be automated for the preparation of nanoparticle dilutions and the reading of the MIC [303,316,320,321].

Although all the results presented in Table 1, Table 2, Table 3, Table 4, Table 5, Table 6, Table 7 and Table 8 and S1–S7 confirm the reliability of the diffusion and dilution methods for the MNP susceptibility testing of different microbes, only a very limited number of studies have compared the MIC values resulting from the dilution method with the MIC values (or DOIs) obtained by the diffusion method, or other methods, such as the E-test. The objective would be to identify the most feasible, quantitative and cost-effective methods for high-throughput MNP susceptibility testing that can be used in any laboratory setting and for any clinical bacteria and fungi. With respect to future studies, the comparison of data from dilution versus diffusion methods will likely guide the reader in choosing which is most appropriate for particular microorganisms, experimental conditions or biomedical applications.

### 4.2. Factors Influencing the Evaluation of Antimicrobial Activities of Metallic Nanoparticles

Factors that can influence either the diameter of the inhibition zone or the MIC, and modify the antibacterial and antifungal activities of MNPs include: (i) the types and species of bacteria and fungi, (ii) the diversity of strains within bacterial and fungal species, (iii) the inoculum density, (iv) the composition and pH of the culture medium, (v) the disc application timing, (vi) the temperature and time of incubation, (vii) the depth of the agar medium, (viii) the spacing of the antibiotic discs, (ix) the nature and concentration of the capping and stabilizing agents, (x) the size, shape and zeta potential of nanoparticles, (xi) the type, content and quality control of antibiotic discs, (xii) the impregnation of nanoparticles in sterile blank discs, and (xiii) the additive and synergistic effects of antimicrobial nanoparticles in combination with conventional or non-traditional antibiotics [303,321,322,323]. The impact of some of these parameters on the antimicrobial assessment of MNPs is discussed in the following paragraphs.

#### 4.2.1. Types of Bacterial and Fungal Species and Strains

The human body hosts a whole community of commensal, saprophytic and pathogenic microorganisms grouped under the term “microbiota” [324,325].

The skin or flora microbiota can be resident or transient. The resident bacteria are dominated by commensal bacteria (e.g., *Staphylococcus epidermidis*, *Staphylococcus warneri*) and coryneform bacteria (e.g., *Brevibacterium epidermidis*, *Arthrobacter globiformis*, *Corynebacterium* spp.). In contrast, the transient microbiota are composed of saprophytic microorganisms (e.g., *Propionibacterium acnes*) and opportunistic pathogenic bacteria (e.g., *Staphylococcus aureus*, *Pseudomonas aeruginosa*, *Bacillus* spp., *Treponema pallidum*, *Acinetobacter johnsonii* and *Acinetobacter baumannii*) [326,327]. Primary cutaneous mold infections are predominantly caused by *Aspergillus flavus*, *Aspergillus niger*, *Fusarium* spp., *Mucor* spp., *Rhizopus stolonifer*, *Malassezia globosa*, *Trichosporon cutaneum*, *Alternaria alternata*, *Candida albicans*, *Torulopsis* spp., *Trichosporon cutaneum*, *Penicillium* spp., etc. [326,327,328,329].

The oral flora is largely dominated by anaerobic bacterial genera, such as *Actinomyces*, *Bacteroides*, *Lactobacillus*, *Leptotrichia*, *Treponema*, *Streptococcus* and *Peptococcus*. Several kinds of microfungi, such as *Aspergillus*, *Candida*, *Cladosporium*, *Cryptococcus*, *Fusarium* and *Penicillium* are also found in the mouth [330].

In the small intestine and the colon, the intestinal microbiota is mostly represented by *Escherichia coli*, *Helicobacter pylori*, *Pseudomonas aeruginosa* (bacteria), *Candida albicans* and *Saccharomyces boulardii* (fungi) [331].

**Figure 7:**
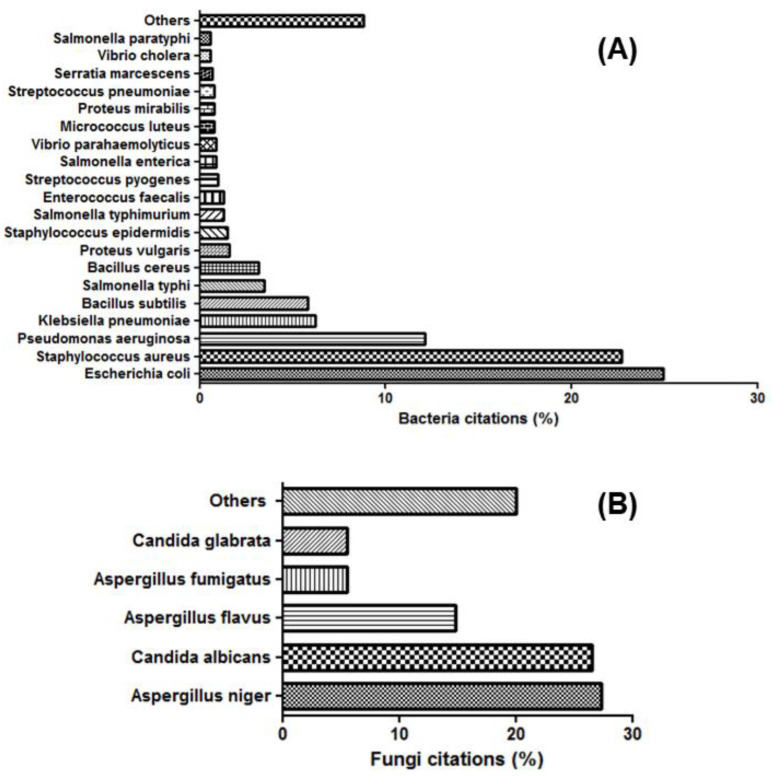
(**A**) Percentage of bacteria citations (*n* = 2065). Others (8.8%) include ca. 70 bacterial species, such as *Acinetobacter baumannii*, *Bacillus megaterium*, *Enterobacter cloacae*, *Klebsiella planticola*, *Pseudomonas putida*, *Staphylococcus saprophyticus* and *Shigella dysenteriae*. (**B**) Percentage of fungi citations (*n* = 326). Others (20.0%) include ca. 15 fungal species, such as *Aspergillus terreus*, *Candida krusei*, *Candida freundii*, *Fusarium oxysporum*, *Penicillium italicum*, *Penicillium notatum* and *Phanerochaete sordida*.

Despite the high number of (opportunistic) pathogenic microorganisms, most research papers are limited to assessing the antimicrobial susceptibility of three bacterial species (*Staphylococcus aureus*, *Escherichia coli* and *Pseudomonas aeruginosa*) and three fungal isolates (*Aspergillus flavus*, *Aspergillus niger* and *Candida albicans*) (Table 1, Table 2, Table 3, Table 4, Table 5, Table 6, Table 7 and Table 8 and S1–S7; Figure 7) [9,192,332,333,334,335,336,337]. However, testing the antibacterial and antifungal susceptibility of a large number of pathogenic microorganisms from cutaneous, oral and intestinal microbiota would help to expand the spectrum of activity of biosynthesized MNPs. Additionally, although most studies attest that Gram-negative bacteria are more sensitive to antimicrobial nanoparticles than Gram-positive bacteria [338,339,340,341,342], it is important to always confirm this rule with a broader range of green MNPs, as well as bacterial and fungal species and strains. By doing so, the chances of detecting certain exceptions increases, as previously reported [9].

**Figure 8:**
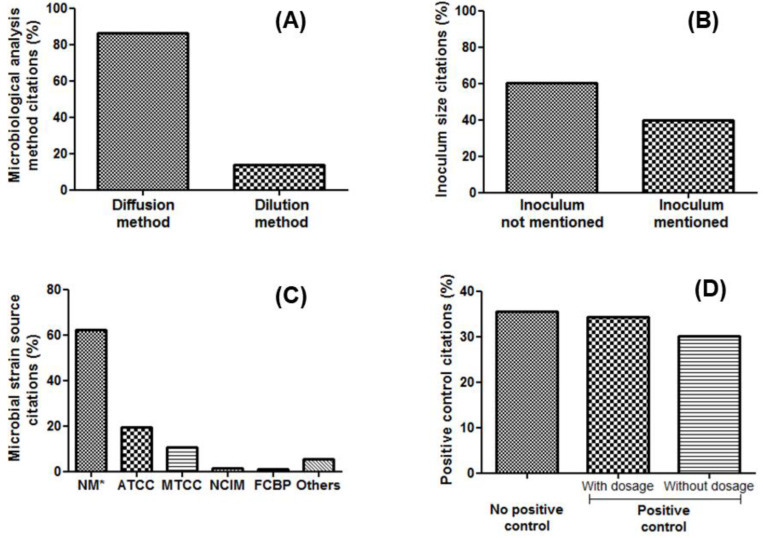
(**A**) Percentage of microbiological analysis citations (*n* = 635). (**B**) Percentage of inoculum size citations (*n* = 635). (**C**) Percentage of microbial strain source citations (*n* = 2391). Others (5.4%) include CMCC (China Medical Culture Collection), KACC (Korean Agricultural Culture Collection), KCCM (Korean Culture Center of Microorganisms) and PTCC (Persian Type Culture Collection) (NM* = not mentioned). (**D**) Percentage of positive control citations (*n* = 752).

Most of the microbiological strains that have been tested for their susceptibility against green-synthesized metallic nanoparticles were procured from the American Type Culture Collection (ATCC) [121,343,344,345], the Microbial Type Culture Collection (MTCC) [274,346,347,348], or the National Collection of Industrial Microorganisms (NCIB) [274,334,348] (Table 1, Table 2, Table 3, Table 4, Table 5, Table 6, Table 7 and Table 8 and S1–S7, Figure 8). However, laboratory strains of the same bacterial and fungal species may show differences in sensitivity against MNPs [349]. This is particularly the case for β-lactamase-negative and β-lactamase-positive laboratory strains (e.g., *E. coli* ATCC 25922 vs. 35218, and *S. aureus* ATCC 25923 vs. 38591). Therefore, it is recommended that the ATCC, MTCC or NCIB strain numbers of all microorganisms are recorded. Moreover, it would be desirable to assess and compare the antimicrobial activities of green MNPs on these different strains of bacterial and fungal species.

It is worth noting that microbial strains from laboratory and environmental sources generally have lower virulence than their corresponding clinical strains [350,351,352,353]. Consequently, discrepancies may be observed in the antimicrobial activity of MNPs against both clinical and laboratory strains. Hence, to better fight nosocomial infections and prevent the emergence of newly emerging clinical strains and multi-drug-resistant bacteria (e.g., methicillin-resistant *S. aureus*, multi-drug-resistant *P. aeruginosa*, etc.), pharmaceutical strategies based on the development of MNPs, and the evaluation of their antimicrobial activity, should include an even greater number of bacterial and fungal pathogens. 

#### 4.2.2. Inoculum Density

CLSI and EUCAST consider inoculum density to be an important variable in susceptibility testing [354,355]. Indeed, if the inoculum is too large, the chances of reducing the diameter of inhibition zones (or enhancing the MIC) of the studied microorganisms by MNPs increases [354,355,356,357,358,359]. The consequence is that sensitive strains can be considered to be relatively resistant when they are not. Conversely, if the inoculum is too small, the zone of inhibition can be enhanced and the MIC reduced, thereby relatively resistant strains can be considered to be sensitive or susceptible [360]. 

According to American, European and Japanese pharmacopeias [361,362,363], pleasing results can be obtained with an inoculum size of ca. 10^8^ CFU/mL. In contrast, the CLSI states that inoculum size ranging from 2 × 10^5^ to 8 × 10^5^ CFU per mL (or 2 × 10^3^ to 10 × 10^3^ viable cells per spot of 10 µL) can produce confluent growth, thereby leading to optimal results (Table 9) [364,365]. However, referring to most published data related to the microbiological analysis of green MNPs, the initial inoculum sizes of tested microorganisms varies from 10^4^ to 1.5 × 10^8^ CFU/mL [195,235,301,366,367]. Regrettably, we note that some research groups do not mention this density at all (Table 1, Table 2, Table 3, Table 4, Table 5, Table 6, Table 7 and Table 8 and S1–S7 and Figure 8).

Hence, to avoid false-positive or false-negative test results (also known as the inoculum effect) [364], the inoculum density must be standardized. To carefully determine the size of the inoculum and to perform colony counts, McFarland standards or calibrators should be used; 0.5 McFarland standards correspond to approximately 1–2 × 10^8^ CFU/mL [358]. The obtained inoculums need to be used within 15 min of preparation in order to avoid the premature growth of microorganisms [358,365].

#### 4.2.3. Agar Depth and Spacing of Impregnated Discs

Among the parameters that can affect the antimicrobial activity of MNPs, the depth of the agar medium and the spacing of discs impregnated with nanoparticles can also be cited. Indeed, a reduced inhibition zone may be obtained on very thick media, while the reverse is true for media that are too thin [373]. To counteract these phenomena, the depth of the agar medium should be between 4 and 10 mm [374,375]. However, it is difficult to know if this recommendation is always applied or not, especially since the agar depth is not mentioned in most of the studies pertaining to MNPs with antimicrobial properties. To ensure a better comparison of data from one study to another, subsequent research should respect recommendations based on the depth of the agar medium.

It is suggested to place a maximum of seven discs (of 10 mm) on 90–100 mm diameter plates to avoid the overlap of different inhibition zones and/or their deformation near the edge of the plates [358,376,377,378]. This reduces artifacts and the difficulty of comparing microbiological data from one study to another. Once again, very little information on agar depth and the spacing of discs can be obtained from the literature related to MNPs with antimicrobial properties. Nevertheless, based on some data available in the literature, it appears that this provision is not respected in many studies.

#### 4.2.4. Timing of Disc Application

The timing for application of discs impregnated with MNPs on the agar medium (diffusion method) and the dilution of nanoparticle solutions in the microorganism suspensions in glass test tube dilution (macrodilution method), or microtiter plastic plates containing 96 wells (microdilution method), are also considered as major variables that can influence the antibacterial and antifungal activities of MNPs [358]. 

Based on CLSI and EUCAST guidelines, disc application and dilution operations should be performed within 15 min following inoculum preparation [359]. Indeed, if the plates, tubes and microplates seeded with the tested microorganisms are left at room temperature for periods longer than the standard time, microorganisms can start growing before the application of discs or dilutions of nanoparticle solutions. By reducing the diameter of the inhibition zone and/or increasing the MIC values, these phenomena may result in a susceptible strain being wrongly reported as resistant [360].

#### 4.2.5. Temperature and Time of Incubation

As recommended by CLSI and EUCAST, antimicrobial susceptibility testing of MNPs should be performed from 16 to 20 h at 35 ± 2 °C against bacteria, and from 48 to 72 h at 25–30 °C against fungi (Table 9) [379,380]. However, most studies related to the evaluation of antimicrobial activity of MNPs have adopted an incubation period of 12–24 h at 30–37 °C for bacteria, and of 48–72 h at 37 °C for fungi (Table 1, Table 2, Table 3, Table 4, Table 5, Table 6, Table 7 and Table 8 and S1–S7). If the temperature is lowered, the time required for achieving the growth of microbes can be extended, resulting in a “false” larger diameter of inhibition (or lower MIC) [379,380]. On the other hand, high temperatures can negate the results by inhibiting the antimicrobial properties of the biofabricated MNPs [360].

With the aim of determining the bactericidal and fungicidal effects of nanoparticles as a function of incubation time, a time-kill test can be performed by incorporating a bacterial or fungal suspension and different concentrations of MNPs (e.g., 0.25 MIC, 0.5 MIC, 1 MIC, etc.) in different tubes or microplates containing the broth culture medium [319,381]. The percentage of dead cells at various time intervals (0.5 h, 1 h, 6 h, 12 h, etc.) can then be calculated relative to the growth control by determining the number of living cells present in each tube or microplate according to validated count methods [318,381]. Generally, the bactericidal effect is obtained with a lethality percentage of 90% for six hours, which is equivalent to 99.9% of lethality for 24 h. Additionally, survivor counts from collecting sequential samples are generally plotted to obtain a “time-kill curve” after an incubation period at a predetermined temperature [382,383]. Therefore, standardization of temperature and time of incubation is required to ensure the comparison of results from one study to another.

#### 4.2.6. Size and Shape of Nanoparticles

The antibacterial and antifungal properties of MNPs depend strongly on their sizes and shapes. Small nanoparticles (diameter sizes ˂ 30 nm) show better antimicrobial activity than large nanoparticles (diameter sizes ˃ 30 nm) [384,385,386], since smaller particles offer a greater surface area for contact, favoring MNP-microbial cell interactions, as well as, more importantly, physical damage to microbial membranes. A number of nanoparticles per bacterium is required to induce cell death [387,388]. Additionally, the ability of small nanoparticles to freely permeate inside the microbial membrane is higher than that of large nanoparticles [387,388].

It has been observed that needle-shaped metal oxide nanoparticles generally exhibit significantly greater antimicrobial activity than those with a cube shape [389]. Additionally, truncated triangular silver nanoparticles were found to be more effective against *E. coli* than rod-shaped silver nanoparticles, while rod-shaped particles were much more effective than spherical nanoparticles [390,391]. 

Therefore, the size and shape of antimicrobial MNPs must be considered when comparing their MIC values and the diameter sizes of inhibition. However, a small number of authors omit to provide information relating to these physicochemical characteristics (see Table 1, Table 2, Table 3, Table 4, Table 5, Table 6, Table 7 and Table 8 and S1–S7).

The ratio between the metal salts and plant extracts used during biosynthesis must also be taken into account, since the amount of capping agents (phytoconstituents) on the surface of MNPs, as well as the size, shape and antimicrobial activities of the nanoparticles, depends on it [392,393]. The seasonal and geographical variability of plant compositions should also be considered. Hence, the collection periods and sites should be referred to in all studies, although this has not always been the case to date. 

#### 4.2.7. Zeta Potential of Nanoparticles

The zeta potential (or charge surface) is one of the critical properties of nanoparticles that can affect their stability and cell adhesion [18,394]. Balanced by oppositely charged counter ions present in the surrounding media, the surface charge of bacterial and fungal membranes is generally negative [395]. Hence, upon interaction with these membranes, green MNPs with positive surface charges may impede the growth of bacteria and fungi by preventing their attachment [385,388,396,397,398]. Moreover, the capacity of cationic nanoparticles to improve the production of reactive oxygen species, and the exertion of mechanical stress on the microbial membrane, appear to be greater than that of anionic and neutral nanoparticles [280,281]. 

The presence, or addition, of certain cationic agents (e.g., polymyxin, cetyltrimethyl ammonium bromide (CTAB), benzalkonium chloride and chlorhexidine digluconate) to the culture media can modify the zeta potential of bacterial and fungal cell membranes through electrostatic interactions. This may subsequently alter cell surface permeability, leading to the death of bacteria and fungi [399]. In addition, aminoglycoside antibiotics (e.g., gentamicin, neomycin, paromomycin) that are positively charged at physiological pH can synergize with all kinds of MNPs [400,401]. On the other hand, depending on their zeta potential, phytocomponents that act as stabilizing and capping agents can reduce the propensity of biosynthesized nanoparticles to aggregate, which influences their size, shape, stability and biological activity [392,402].

It should be remembered that MHB is a culture medium recommended by the US Food and Drug Administration, World Health Organization, EUCAST and CLSI for testing the susceptibility of aerobic and facultative anaerobic bacteria to antimicrobial agents [356,403]. In addition, cation-adjusted MHB 2 (CAMHB) broth, which corresponds to MHB in which divalent ions (e.g., calcium and magnesium) have been adjusted, can greatly reduce the MIC of several antibiotics (e.g., daptomycin, quinolones, aminoglycosides, and tetracycline) by affecting their mode of action and stability [356]. It is not ruled out that this phenomenon may also be observed with greenly biosynthesized MNPs, although, to the best of our knowledge, there is no study of this to date.

It is advisable to measure and indicate the zeta potential of dispersions containing MNPs and other components. As well as the effects of the interfacial potential on the antimicrobial propensity of MNPs, all the phenomena that affect the zeta potential should be studied more extensively, particularly when evaluating the efficacy of MNPs in combination with cationic antimicrobial agents.

#### 4.2.8. pH of Culture Media

As mentioned earlier, phytoconstituents (e.g., flavonoids, tannins, alkaloids, etc.) from plant extracts used in green synthesis can cap the surface of biogenic MNPs through highly complex mechanisms of reduction of metal ions and nucleation/stabilization of reduced metal atoms [393]. All these phytochemical metabolites contribute to the antibacterial and antifungal activity of MNPs, and reduce toxicity by passivation of the surface [393,404].

Notably, if the pH of the culture medium is too low, some phytoconstituents (e.g., amino acids, proteins), and antimicrobial agents used as positive controls (e.g., quinolones, macrolides, aminoglycosides), may lose their potency, while other antibacterial drugs, such as tetracyclines, may show enhanced efficacy [405,406,407]. On the other hand, certain phytocomponents, such as caffeic, chlorogenic and gallic acids, as well as antimicrobial proteins, become unstable at high pH values, thereby losing their biological and pharmacological activity [408]. Additionally, the pH of the culture media can impinge on or modify the surface charge density and distribution of both metals and phytoconstituents capped on the surface of MNPs. This phenomenon could explain why green-synthesized silver nanoparticles and zinc nanoparticles become less active in acidic and basic media, respectively [409,410,411,412,413,414]. 

Therefore, the pH (and ionic strength) of culture media should be standardized to maintain the physical and biological properties of the metallic and phytochemical components of biogenic MNPs. The pH values of the culture media should range between 6.8 and 7.4, irrespective of the temperature [359].

#### 4.2.9. Antibiotic and Antifungal Reference Standards

To assess the antimicrobial activity of MNPs, a good and common practice consists of testing them side-by-side with well-known antibiotic and antifungal references, and comparing the resultant antimicrobial activity [301,415,416]. Biosynthesized MNPs would be considered more active than a standard antimicrobial agent if the latter has a higher MIC (or lower diameter of inhibition zone) than the nanoparticles.

Depending on the MICs and the diameters of the inhibition zone, microorganisms can be considered “sensitive” or “resistant”. These values are generally compared with those mentioned in local microbiology guidelines and/or the “antibiotic and antifungal disc interpretative criteria and quality control table” proposed by EUCAST and CLSI [368,379,417,418]. The susceptibility of microorganisms to MNPs improves with decreasing MICs (or increasing diameters of inhibition) [419]. 

To select the most suitable antimicrobial reference test and compare its in vitro activity to MNPs, investigators can use breakpoint tables or performance standards for antimicrobial susceptibility testing [358,418,420]. Nevertheless, drug purity and disc potency (i.e., amount of drug per disc) should be considered for a more in-depth comparison of activities. Indeed, these two parameters can be reduced owing to deterioration during storage, thereby decreasing the antimicrobial efficacy of the standard test [418].

It would be interesting to simultaneously evaluate MNPs, antimicrobial standards, and test microorganisms alongside a standard microorganism having known MIC or inhibition diameter values. This strategy would enable confirmation of the reliability of the experimental conditions and the obtained results [360].

Representative (nano)antimicrobial agents can be dissolved in a suitable solvent (dilution method) or impregnated in discs (diffusion method) [303,309]. However, homogenous and reproducible disc impregnation of MNPs that are manufactured in solid (powder) or semi-solid (cream or ointment) form constitutes a bottleneck that hampers the reliable correlation of the diameter of inhibition with antimicrobial activity. To remedy this problem, the agar dilution method can be used as an alternative [307,308]. For this purpose, a volume or weight of varying concentrations of metallic nanoparticle preparations can be incorporated in different wells. Nevertheless, to obtain more comparable results, it would be necessary to standardize the volume of the wells and the quantity of the tested samples.

#### 4.2.10. Synergistic Activity of Nanoparticles with Antimicrobial Substances

In exploring alternative approaches to improving the therapeutic management of infectious diseases, the combination of MNPs with antimicrobials (e.g., amoxicillin, azithromycin, cefotaxime, cefuroxime, chloramphenicol, clindamycin, erythromycin, fosfomycin, penicillin G, vancomycin, etc.) can be effective [400,421,422,423,424]. This strategy exhibits enhanced antibacterial and antifungal effects against different types of microbes in comparison with MNPs and antimicrobial agents alone [400,421,422,423,424].

To better evaluate this approach and ensure appropriate comparison of results from one study to another, several parameters must be considered and standardized. This particularly concerns the composition of the culture media, in terms, for example, of cationic substances (e.g., CAMHB), the spectrum of activity, and the efficacy of the selected antimicrobials, as well as the concentration ratio of the latter with MNPs.

## 5. Methods for Testing Anti-Biofilm Activities of Green Metallic Nanoparticles

To quantify cells in biofilm-grown microbes, many direct and indirect counting methods have been developed [425]. Direct measurement methods include viable cell enumeration using plate counts, microscopic cell counts, Coulter cell counting, flow-based cell counting and fluorescence microscopy. In addition, indirect counting methods comprise microplate assays, dry mass determination, total organic carbon, total protein, ATP bioluminescence and quartz crystal microbalance, Calgary biofilm device, biofilm ring test, etc. Of all these methods, microplate plate models are the most commonly used for the evaluation and measurement of the antibiofilm activity of green MNPs [57,425,426,427,428].

Quantitative methods of biofilm characterization are often accompanied and assisted by qualitative methods (e.g., scanning electron microscopy, scanning electrochemical microscopy) for imaging the surface roughness, morphology and spatial organization of biofilms, as well as their interaction with the environment [425,427]. 

### 5.1. Microplate Assays

As stated above, microplate plate assays remain among the most frequently used methods for evaluating the antibiofilm activities of green MNPs. Microtiter plate methods are relatively inexpensive, easy to perform, rapid and reproducible. Additionally, these methods have great flexibility due to their many variations or modifications, such as the tetrazolium salt assay and the crystal violet assay [426].

Among the most widely used tools in biology for real-time evaluation of cellular viability and metabolism in vitro, tetrazolium salt assays allow direct and indirect measures of biofilm growth via UV-visible and fluorescence spectroscopy [426]. The different tetrazolium salts are metabolically converted to formazan derivative crystals which are solubilized in dimethyl sulfoxide (DMSO) before being quantified by spectrophotometry [426]. In the crystal violet assay, biofilm cells are stained with crystal violet dyes and then infused with decoloring solution (e.g., pure ethanol, acetic acid, methanol, etc.). The resulting solution is generally transferred to clean 96-well plates to assess the optical density (OD) or absorbance at 530–600 nm using a microtiter plate reader. Homogenous resolubilization of crystals and dyes according to recommended protocols enables precise measurement of biofilm production since microplate readers measure the optical density only at one point in the middle of the well [57,429,430,431,432,433,434,435,436,437,438].

### 5.2. Factors Influencing the Evaluation of Antibiofilm Activities of Metallic Nanoparticles

Various static and batch-growth conditions may influence biofilm formation by different species and strains of bacteria and fungi in microtiter plates. These testing conditions include the storage of microorganisms, inoculum density, culture medium, microtiter plate, cultivation of biofilm, washing, fixation and staining. All these factors are discussed below.

#### 5.2.1. Storage Conditions

When stored by freezing at −70 °C or by lyophilization, microorganisms generally maintain their virulence properties once thawed from storage. However, this is not the case with some fastidious bacteria and fungi (e.g., *Staphylococcus* spp.) which can produce mixtures of phenotypes differing in their ability to form biofilms [426]. The influence of storage conditions should be considered during the evaluation of antibiofilm activities. However, most studies relating to the evaluation of the activity of MNPs against staphylococcal-based biofilms do not mention it.

#### 5.2.2. Type of Microorganisms

Biofilm formation depends on the type of microorganisms selected for the experiment [439]. According to the literature, MNPs are mainly evaluated against *Escherichia coli*, *Staphylococcus aureus*, *Pseudomonas aeruginosa* and *Candida albicans*. Other microorganisms include *Staphylococcus epidermidis*, *Enterococcus faecium*, *Enterococcus faecalis*, and *Klebsiella pneumoniae*. For all these microorganisms, the strain numbers assigned in international culture collections should be reported (e.g., *Staphylococcus epidermidis* ATCC 35984 (high slime producer) vs. *Staphylococcus epidermidis* ATCC 1228 (non-slime producer), or *Staphylococcus aureus* ATCC 29213 (methicillin-sensible) vs. *Staphylococcus aureus* ATCC 43300 (methicillin-resistant)). For clinical or environmental isolates, all available and relevant background and ethical information should be reported [426,440].

#### 5.2.3. Inoculum Density

Biofilm density increases with increasing initial inoculum. The inoculum size can vary between 10^3^ and 10^8^ CFU/mL [57,429,430,431,432,433,434,435,436,437,438]. Nevertheless, several authors consider that a microbial cell size of 1 × 10^8^ CFU/mL is suitable for studies dealing with the evaluation of antibiofilm activities of MNPs [51,429]. As a result, the inoculum density should be carefully standardized when evaluating antibiofilm properties, as when determining the antibacterial and antifungal activities of MNPs. However, we regret to note that the inoculum density is not given in some studies, and that it is not determined with precision in other studies.

The inoculation preparation (e.g., culturing methods) can affect the ability of microorganisms to attach to a surface [441,442]. However, information concerning inoculation preparation (e.g., concentration, temperature, time, temperature, growth phase, shaking conditions, growth media, humidity, CO_2_) is often not reported in published papers related to MNPs.

Cell density can also affect the spreading and clustering of certain microorganisms [443]. The percentages of clusters and of total clustered cells increase linearly with the density of inoculum (but are not time-dependent). Indeed, in the presence of biofilm-associated clusters, preexisting cell clusters that can form in cell suspension may lead to false-positive results [429]. Hence, to avoid inoculation of these preexisting clusters, cell suspensions should be broken up with a syringe fitted with a 23-gauge needle and then with a vortex [443]. These issues are of great importance and must be taken into account by researchers who are interested in evaluating the antibiofilm activities of MNPs. 

#### 5.2.4. Culture Medium

The ability of bacteria and fungi to produce biofilms under in vitro conditions depends strongly on the composition of the medium, which itself varies from one supplier to another [426]. The agar media closely resemble the surfaces (catheters, prostheses, etc.) found in in vivo situations. Hence, during biofilm formation, surface-associated bacteria and fungi can adhere better to solid growth media than to liquid culture media [444,445]. Nevertheless, in the absence of a clear-cut recommendation on the medium to be used for testing bacteria-based biofilm formation, some authors use Trytic soy broth (TSB) and brain heart infusion (BHI) broth, supplemented with glucose, sodium chloride or ethanol [429,430,431,437,446,447,448,449,450]. 

For fungi, yeast nitrogen base (YNB), yeast peptone dextrose (YPD), Roswell Park Memorial Institute–1640 (RPMI-1640) medium, artificial saliva medium (ASM), Sabauraund dextrose broth (SDB) or phosphate-buffered saline (PBS) can also be used, with or without supplement [429,436,438,450,451,452,453,454,455,456].

Therefore, the choice of medium for biofilm cultivation is an essential element in the process aimed at standardizing the analytical methods of antibiofilm activities of MNPs. Many efforts must be made in this direction. 

#### 5.2.5. Type of Microplates

To mimic the surfaces to which microorganisms can adhere to form biofilms, both tissue and non-tissue culture microtiter plates can be used [429]. However, cell attachment and proliferation were better on surface-treated tissue culture plates than on non-tissue culture plates [457,458]. Nevertheless, tissue-culture-treated plates from different manufacturers may provide different conditions for the cultivation and proliferation of biofilms. On the other hand, flat-bottomed microtiter plates allow better biofilm quantification than U-shaped and V-shaped microtiter plates [426,457,459]. Hence, for more transposable and comparable results, all these differences should be noted and considered by research groups. All the microplate characteristics (e.g., color of plate, number of wells, material, pre-coating conditions, etc.) should also be taken into account as part of an overall standardization process for microplate assays [426].

#### 5.2.6. Time and Temperature of Incubation

The duration and temperature of incubation are important parameters for the cultivation of biofilms; indeed, the density of biofilms is dependent on these two parameters [443,460]. Covered with a lid, the inoculated plate should be incubated aerobically for 24 ± 0.5 h at 36 ± 1 °C under static conditions [426,429]. However, some authors incubate microtiter plates for 18 h or 20–24 h, while others incubate overnight [461,462,463]. In addition, literature reports suggest that some investigators prolong their incubation time for 48 h [426,464]. 

All this indicates a lack of standardization in the operating conditions of incubation. This situation can generate both false-positive and false-negative results. Even more so, it can negatively impact on the possibility of comparing the results from one study to another.

#### 5.2.7. Washing, Fixation and Staining Steps

The washing steps aim to remove non-adherent cells and unbound dye on biofilm-containing microplates after the formation of biofilms. However, excessive washing may lead to false-negative results. In contrast, false-positive results can be obtained from insufficient washing. To avoid this, three- and four-step washing protocols are advisable [429]. 

A variety of methodologies and techniques can be applied during the washing step. However, some can lead to false-positive results (e.g., washing robots), while others can cause disruption of biofilm layers (e.g., mechanical plate washers). As a result, washing using micropipettes followed by emptying by flicking is considered to be a simple and effective method which is applied by several investigators [429].

Colorimetric methods have also been used to quantify metabolic activity in biofilms. For this purpose, tetrazolium salts and resazurin can be used. Compared to tetrazolium salt assay, resazurin-based quantification is inexpensive and less time-consuming. Moreover, it offers a good correlation with CFU counts. Nevertheless, one of the drawbacks of the colorimetric method using resazurin is the high lower limit of quantification (˃10^6^–10^7^ CFU/biofilm). An alternative approach for this method is currently available, decreasing the lower limit of quantification to 10^3^ CFU/biofilm [429].

Extensive detachment and removal of sessile bacterial cells can result from the stringency of washing [426,429]. To prevent this generating artifacts or false results, washing must be followed by a fixation step with absolute ethanol, methanol or heat fixation at 60 °C for 1 h. After this last step, the adherent biofilm layers present in microtiter plates must be stained with dyes, such as crystal violet and alcian blue. Alcian blue stains both live and dead cells, as well as other components found in the matrix of the biofilm matrix, and is, therefore, well suited to the quantification of total biofilm biomass. However, unlike alcian blue, the crystal violet dye does not color slimy material. Other limitations of crystal violet include a lack of reproducibility, its non-specific nature in poly-microbial communities, and the absence of standardized protocols. Nevertheless, crystal violet remains the most frequently used strain for biofilm quantification [425,426,427,429].

## 6. Conclusions and Perspective

When searching the literature on green MNPs from 2010 to date, it appears that most of the published data are related to MNP synthesis (e.g., methods, mechanisms, influence of parameters), physicochemical characterization (e.g., size, zeta potential), and the evaluation of antimicrobial and antibiofilm properties and mechanisms of action. Of the 600 articles dedicated to antimicrobial metal nanoparticles that we have presented in this review, very few were focused on factors that affect the pharmacological properties of these MNPs and the outcomes of their in vitro evaluation. 

Based on this, we set out to critically examine the experimental conditions and essential factors that can affect the antimicrobial performance of MNPs, enabling pinpointing of important MNP quality attributes for the effective development of design rules in the field of biogenic nanotechnology for tackling infectious diseases. 

The present review provides, firstly, an understanding of what is commonly reported in scientific articles related to antimicrobial and antibiofilm MNPs. Secondly, this review highlights the critical procedures and parameters that influence the antimicrobial and antibiofilm evaluation of MNPs.

In addition to the lack of detailed information regarding the chemical and biological materials, as well as the laboratory equipment used, insufficient knowledge and the non-standardization of experimental protocols and laboratory conditions make it difficult to produce reproducible and reliable data or to gain detailed insights into comparative studies. This situation has been made worse by the progressive limitation in the number of words in several journals. 

To address the issues arising from these multifaceted problems, the elaboration and use of minimal information guidelines or reporting standards related to the evaluation of antimicrobial and antibiofilm activities would constitute an effective strategy and approach. Minimal information guidelines should provide a guide for researchers on the necessary information that a manuscript should include for specific experiments. However, the elaboration of these guidelines requires a variety of studies, a dialog among experts and a consensus by the research community. In the interim, we have presented this draft which includes the minimum operating conditions required which would facilitate reproducibility and the reliable comparison of research data. By applying a standardized approach to experimental conditions for the evaluation of antimicrobial and antibiofilm activity, researchers will push the field of biogenic nanotechnology to new frontiers towards the development of high-value antimicrobial MNPs that can attack both existing and emerging antimicrobial resistant strains.

## Figures and Tables

**Table 4:** Antimicrobial green-synthesized zinc oxide nanoparticles.

Plant Type	Part Used	Operative Conditions for Synthesis	NP Characteristics (Shape and Size)	Microbiological Analyzes (Operative Conditions)			Refs.
				Methods, Incubation Temperature, Incubation Time, pH, Inoculum Density, Positive Control	Tested Bacteria and Fungi	MIC, ZOI or PI *	
*Cassia alata*	Leaves	Zinc acetate 10 mM/plant extract 10% (1:1 *v*/*v*) 80 °C 2 h pH 12	Spherical 60–80 nm	Dilution 37 °C 24 h pH NM 1 × 10^5^ CFU/mL No control	*E. coli*	20 μg/mL	[212]
*Trifolium pratense*	Flowers	Zinc oxide 500 mM/plant extract 2.25% (1:1 *v*/*v*) 90 °C 24 h pH 6	Hexagonal 60–70 nm	Diffusion 35 ± 1 °C 18 h pH NM 5 × 10^5^ CFU/mL No control	*S. aureus* ATCC 4163 *E. coli* ATCC 25922 *P. aeruginosa* ATCC 6749 *S. aureus* (clinical strain) *P. aeruginosa* (clinical strain)	31 31 28 31 29 mm	[213]
*Pongamia pinnata*	Seed	Zinc acetate 20 mM/plant extract 20% (4:50 *v*/*v*) 60 °C 2 h pH 12	Spherical 30–40 nm	Diffusion 37 °C 24 h pH NM Inoculum size NM Ciprofloxacin 5 μg	*P. aeruginosa* HQ 693274.1 *Bacillus licheniformis* M235407.1 *Vibrio parahaemolyticus* HQ 693275.1	14 17 12 mm	[214]
*Plectranthus amboinicus*	Leaves	Zinc nitrate 0.05 mM/plant extract 12% (1:5 *v*/*v*) 150 °C 6 h pH NM	Hexagonal 20–50 nm	Diffusion 37 °C 24 h pH 7.4 1 × 10^5^ CFU/mL No control	*S. aureus* ATCC 33591	13 mm	[215]
*Stevia rebaudiana*	Leaves	Zinc acetate 100 mM/plant extract 14% (1:1 *v*/*v*) 70–80 °C 2 h pH NM	Rectangular 10–90 nm	Dilution 37 °C 24 h pH NM 1.5 × 10^8^ CFU/mL No control	*S. aureus* *E. coli*	2 2 µg/mL	[216]
*Silybum marianum*	Seed	Zinc sulfate 1 mM/plant extract 6% (1:50 *v*/*v*) 37 °C 24 h pH 12	Flowers 60 nm	Diffusion 37 °C 24 h pH NM 5 × 10^6^ CFU/mL Cefixime ** Roxithromycin **	*S. aureus* ATCC 6538 *K. pneumoniae* ATCC 1705 *B. subtilis* ATCC 6633 *E. coli* ATCC 25922 *P. aeruginosa* ATCC 15442	20 17 9 10 17 mm	[217]
*Linum usitatissimum*	Root	Zinc nitrate 0.1 mM/plant extract 10% (1:10 *w*/*v*) 60 °C 3 h pH NM	Hexagonal 35 nm	Diffusion 37 °C 24 h pH NM Inoculum size NM Amoxicillin 10 µg/mL	*S. aureus* ATCC 6538 *E. coli* ATCC 15224 *K. pneumoniae* ATCC 4619	14 14 12 mm	[218]
*Anchusa italic*	Flowers	Zinc acetate 100 mM/plant extract 25% (1:10 *v*/*v*) 70 °C 6 h pH NM	Hexagonal 7.6 ± 2.0 nm	Diffusion 37 °C 24 h pH NM 1 × 10^8^ CFU/mL No control	*Bacillus megaterium* *S. aureus* *E. coli* *Salmonella typhimurium*	13.6 14.6 13 14.4 mm	[219]
*Conyza canadensis*	Leaves	Zinc nitrate 150 mM/plant extract 6% (1:2 *v*/*v*) 80 °C 20 min pH NM	Spherical 10–50 nm	Diffusion 37 °C 24 h pH NM Inoculum size NM Ciprofloxacin 0.5 mg	*E. coli* *S. aureus*	16 14 mm	[220]

* MIC = minimal inhibition concentration; ZOI = zone of inhibition; PI = percentage of inhibition. ** The quantity or concentration is not mentioned. NM = not mentioned.

**Table 5:** Green platinum nanoparticles exhibiting antimicrobial activity.

Plant Type	Part Used	Operative Conditions for Synthesis	NP Characteristics (Shape and Size)	Microbiological Analyzes (Operative Conditions)			Refs.
				Methods, Incubation Temperature, Incubation Time, pH, Inoculum Density, Positive Control	Tested Bacteria and Fungi	MIC, DOI or PI *	
*Garcinia mangostana*	Fruit	Hexachloroplatinic acid 1 mM/plant extract 3% (1:1 *v*/*v*) 50–70 °C 15 min pH NM	Spherical 20–25 nm	Diffusion 35 °C 24–48 h pH NM 1 × 10^5^ CFU/mL Penicillin G 2 μg Methicillin 5 μg Vancomycin 30 μg Gentamicin 50 μg Streptomycin 10 μg Ciprofloxacin 5 μg Azithromycin 30 μg Clotrimoxazol 25 μg	*Staphylococcus* spp. *Bacillus* spp. *Pseudomonas* spp. *Klebsiella* spp.	10 0 12 11 mm	[224]
*Citrus sinensis*	Peel	Hexachloroplatinic acid 10 mM/plant extract 10% (9:1 *v*/*v*) 80 °C 24 h pH NM	Spherical 50 nm	Diffusion 30 ± 1 °C 24 h pH 4 Inoculum size NM No control	*Aeromonas hydrophila*	4 mm	[225]
*Sechium edule*	Fruit	Platinum (II) chloride 1 mM/plant extract 12.5% (1:1 *v*/*v*) 100 ± 5 °C 12 h pH 9	Spherical 28 nm	Diffusion 37 °C 24 h pH NM 5 × 10^5^ CFU/mL Ciprofloxacin 30 μg Cefprozil 30 μg	*B. subtilis* *E. coli*	25 24 mm	[226]
*Spinacia oleracea*	Leaves	Hexachloroplatinic acid 20 mM/plant extract 75% (2:1 *v*/*v*) 100 °C 24 h pH NM	Rod 154 nm	Diffusion 37 °C24 h pH NM 1 × 10^5^ CFU/mL No control	*S. typhi* MTCC 098	13 mm	[227]
*Taraxacum laevigatum*	Powder	Hexachloroplatinic acid 10 mM/plant extract 5% (5:1 *v*/*v*) 90 °C 10 min pH NM	Spherical 2–7 nm	Diffusion 37 °C 24 h pH NM 5 × 10^5^ CFU/mL Streptomycin **	*B. subtilis* *P. aeruginosa*	18 15 mm	[228]
*Cerbera manghas*	Leaves	Hexachloroplatinic acid 1 mM/plant extract 2% (19:1 *v*/*v*) 25 °C 2 h pH NM	Spherical 9–12 nm	Diffusion 37 °C 24 h pH NM 1 × 10^8^ CFU/mL Streptomycin 0.25 mg/mL	*Vibrio cholerae* *S. aureus* *Streptococcus pyogenes* *S. typhi* *E. coli*	20 19 13 12 11 mm	[229]
*Prunus yedoensis*	Gum	Hexachloroplatinic acid 100 mM/plant extract 25% (5:1 *v*/*v*) 80 °C 5 h pH NM	Spherical and oval 10–50 nm	Diffusion 37 °C 48 h pH NM Inoculum size NM Nystatin **	*Phytophthora capsici* *Phytophthora drechsleri* *Didymella bryoniae* *Colletotrichum acutatum* *Cladosporium fulvum*	0 0 0 15 18 mm	[230]
*Curcuma longa*	Seed	Hexachloroplatinic acid 1 mM/plant extract 1% (1:1 *v*/*v*) 80 °C 2 h pH 10	Spherical 9 nm	Dilution 37 °C 3 h pH NM 1 × 10^8^ CFU/mL No control	*E. coli* CD-496 *E. coli* CD-2 *E. coli* CD-3 *E. coli* CD-19 *E. coli* CD-549 *S. aureus* CD-1578 MRSA CD 489	16 16 16 16 16 64 32 nM	[231]

* MIC = minimal inhibition concentration; ZOI = zone of inhibition; PI = percentage of inhibition. ** The quantity or concentration is not mentioned. NM = not mentioned; MRSA = methicillin-resistant *S. aureus.*

**Table 6:** Green palladium nanoparticles with antimicrobial activity.

Plant Type	Part Used	Operative Conditions for Synthesis	Np Characteristics (Shape and Size)	Microbiological Analyzes (Operative Conditions)			Refs.
				Methods, Incubation Temperature, Incubation Time, pH, Inoculum Density, Positive Control	Tested Bacteria and Fungi	MIC, DOI or PI *	
*Moringa oleifera*	Peel	Palladium acetate 10 mM/plant extract 10% (4:1 *v*/*v*) 80 °C 5 min pH NM	Spherical 27 nm	Diffusion 37 °C 24 h pH NM Inoculum size NM Amoxicillin **	*S. aureus* *E. coli*	1 1 mm	[232]
*Prunus yedoensis*	Leaves	Palladium chloride 1 mM/plant extract 25% (9:1 *v*/*v*) 80 °C 30 min pH NM	Spherical 50–150 nm	Diffusion 37 °C 24 h pH NM Inoculum size NM Amoxicillin **	*B. subtilis* *P. aeruginosa*	6 5 mm	[230]
*Cissus quadrangularis*	Stem	Palladium chloride 0.05 mM/plant extract 10% (1:5 *v*/*v*) 37 °C 10 min pH NM	Spherical 12–26 nm	Diffusion 37 °C 24 h pH NM Inoculum size NM No control	*E. coli*	17 mm	[233]
*Camellia sinensis*	Leaves	Palladium chloride 1 mM/plant extract 1% (1:1 *v*/*v*) 40 °C 30 min pH NM	Spherical 6–18 nm	Diffusion 37 °C 24 h pH NM 1 × 10^8^ CFU/mL Streptomycin **	*S. epidermidis* S273 *E. coli* E266	17 14 mm	[234]
*Garcinia pedunculata*	Leaves	Palladium acetate 1 mM/plant extract 20% (2:1 *v*/*v*) 121 °C 15 min pH NM	Spherical 2–4 nm	Diffusion 37 °C 24 h pH NM Inoculum size NM No control	*Cronobacter sakazakii* AMD04	0.3 mm	[235]
*Phoenix dactylifera*	Leaves	Palladium chloride 3 mM/plant extract 10% (5:1 *v*/*v*) 37 °C 10 min pH NM	Spherical 2–5 nm	Diffusion 37 °C 24 h pH NM Inoculum size NM No control	*P. aeruginosa*	26 mm	[236]
*Arabidopsis thaliana*	Leaves	Palladium chloride 5 mM/plant extract 1% (10:1 *v*/*v*) 80 °C 24 h pH NM	Spherical 20–40 nm	Diffusion 37 °C 24 h pH NM Inoculum size NM No control	*S. aureus*	29 mm	[237]
*Acacia senegalensis*	Gum	Tetrachloropalladic acid 1 mM/plant extract 0.2% (1:1 *v*/*v*) 100 °C 6 h pH NM	Spherical 10 nm	Diffusion 37 °C 24 h pH NM 0.5 × 10^5^ CFU/mL No control	*Bacillus cereus* *S. aureus* *Streptococcus agalatiae*	18 16 17 mm	[238]
*Bauhinia variegate*	Bark	Palladium chloride 1 mM/plant extract 10% (4:1 *v*/*v*) 60 °C 30 min pH NM	Irregular 2–9 nm	Diffusion 37 °C 24 h pH NM Inoculum size NM No control	*B. subtilis* MTCC 441 *S. aureus* MTCC 737 *E. coli* MTCC 1687 *C. albicans* MTCC 183	16 6 1 7 mm	[239]
*Allium cepa*	Bulb	Palladium chloride 10 mM/plant extract 10% (1:5 *v*/*v*) 100 °C 2 h pH NM	Spherical 19 nm	Diffusion 37 °C 24 h pH NM 1 × 10^6^ CFU/mL No control	*Bacillus cereus* *S. aureus* *Micrococcus* spp. *E. coli* *Klebsiella* spp. *Proteus* spp.	36 27 40 22 18 17 mm	[240]
*Filicium decipiens*	Leaves	Palladium chloride 1 mM/plant extract 10% (9:1 *v*/*v*) 37 °C 96 h pH NM	Spherical 2–22 nm	Diffusion 37 °C 24 h pH NM 1 × 10^5^ CFU/mL Levofloxacin **	*B. subtilis* *S. aureus* *E. coli* *P. aeruginosa*	12 12 27 24 mm	[241]
*Phyllanthus emblica*	Seed	Palladium acetate 870 mM/plant extract 10% (4:1 *v*/*v*) 60 °C 3 h pH NM	Spherical 28 nm	Diffusion 37 °C 24 h pH NM Inoculum size NM Streptomycin 50 µg/mL	*B. subtilis* *S. aureus* *P. aeruginosa* *Proteus mirabilis*	8.9 8.2 7.6 4.3 mm	[242]
*Eucommia ulmoides*	Bark	Palladium chloride 10 mM/plant extract 20% (5:1 *v*/*v*) 80 °C 30 min pH 6	Spherical 2 nm	Diffusion 37 °C 24 h pH NM Inoculum size NM No control	*S. aureus* *E. coli*	2529 mm	[243]
*Delonix regia*	Leaves	Palladium chloride 0.5 mM/plant extract 25% (9:1 *v*/*v*) 28 °C 3 h pH NM	Spherical 2–4 nm	Diffusion 37 °C 24 h pH NM Inoculum size NM No control	*Streptococcus mitis*	12 mm	[244]
*Coriandrum sativum*	Seed	Palladium chloride 10 mM/plant extract 5% (1:5 *v*/*v*) 60 °C 3 h pH NM	Spherical 113 nm	Diffusion 37 °C 18 h pH NM 5 × 10^8^ CFU/mL Streptomycin 50 µg/mL	*S. aureus* *E. coli* *Salmonella enterica*	13 8.5 10 mm	[245]
*Piper betle*	Leaves	Palladium chloride 1 mM/plant extract 20% (1:10 *v*/*v*) 30 °C 1 h pH NM	Spherical 4 nm	Diffusion 30 °C 48 h pH NM Inoculum size NM Clotrimazol 1 mg/mL	*A. niger*	34 mm	[246]
*Rosmarinus officinalis*	Leaves	Palladium acetate 100 mM/plant extract 10% (2:1 *v*/*v*) 37 °C 24 h pH NM	Spherical 15 nm	Diffusion 37 °C 20 h pH NM 1 × 10^6^ CFU/mL Ciprofloxacin **	*S. aureus* *E. coli* *S. epidermidis* *Micrococcus luteus*	24 25 21 20 mm	[247]
				Difusion 32 °C 1 week pH NM 1 × 10^6^ CFU/mL Nystatin **	*C. albicans* *Candida parapsilolis* *Candida glabrata* *Candida krusei*	0 0 0 0 mm	
*Boswellia serrata*	Gum	Palladium chloride 1 mM/plant extract 0.5% (1:1 *v*/*v*) 121 °C 30 min pH NM	Spherical 2–9 nm	Diffusion 37 °C 24 h pH NM 1 × 10^6^ CFU/mL Gentamicin 10 µg	*S. aureus* ATCC 25923 *P. aeruginosa* ATCC 27853	2628 mm	[248]
*Coffea arabica*	Powder	Palladium chloride 100 mM/plant extract 8% (1:5 *v*/*v*) 60 °C 3 h pH NM	Spherical 20–60 nm	Diffusion 37 °C 24 h pH NM 2 × 10^8^ CFU/mL Ampicillin **	*Enterococcus faecalis* *S. typhi* *S. epidermidis*	12 12 12 mm	[249]
*Morus alba*	Fruit	Palladium chloride 2 mM/plant extract 20% (5:1 *v*/*v*) 80 °C 3 h pH NM	Spherical and non-regular 50–150 nm	Diffusion 37± °C 24 h pH NM 1.5 × 10^8^ CFU/mL Amoxicillin **	*Listeria monocytogenes* ATCC 19115 *E. coli* O157:H7	26 29 mm	[120]

* MIC = minimal inhibition concentration; ZOI = zone of inhibition; PI = percentage of inhibition. ** The quantity or concentration is not mentioned. NM = not mentioned.

**Table 7:** Green copper nanoparticles exhibiting antimicrobial activity.

Plant Type	Part Used	Operative Conditions for Synthesis	NP Characteristics (Shape and Size)	Microbiological Analyzes (Operative Conditions)			Refs.
				Methods, Incubation Temperature, Incubation Time, pH, Inoculum Density, Positive Control	Tested Bacteria and Fungi	MIC, DOI or PI *	
*Cymbopogon citratus*	Leaves	Copper (II) sulfate 0.25 mM/plant extract 50% (2:1 *v*/*v*) 60 °C 3 h pH 12	Spherical, hexagonal and oval 12–14 nm	Diffusion 37 °C 24 h pH NM 1 × 10^7^ CFU/mL No control	*E. coli* ESβL-336 MSSA MRSA	20 18 16 mm	[251]
*Ziziphus spina-christi*	Fruits	Copper (II) sulfate 20 mM/plant extract 6% (1:10 *v*/*v*) 80 °C 1 h pH NM	Spherical 9 nm	Diffusion 37 °C 24 h pH NM Inoculum size NM No control	*E. coli* *S. aureus*	2 2 mm	[252]
*Enicostemma axillare*	Leaves	Copper (II) sulfate 5 mM/plant extract 10% (10:1 *v*/*v*) 50 °C 24 h pH 7	Spherical 44 nm	Diffusion 37 °C 24 h pH NM Inoculum size NM No control	*E. coli* *P. aeruginosa* *K. pneumoniae* *S. aureus* *Proteus vulgaris*	4 6 8 11 8 mm	[253]
*Phyllanthus emblica*	Fruits	Copper (II) sulfate 20 mM/plant extract 50% (3:1 *v*/*v*) 80 °C 15 min pH 10	Flakes 15–30 nm	Diffusion 37 °C 24 h pH NM Inoculum size NM Ciprofloxacin 25 μg	*E. coli* *S. aureus*	14 14 mm	[254]
*Carica papaya*	Leaves	Copper (II) sulfate 5 mM/plant extract 10% (9:1 *v*/*v*) 60 °C 10 min pH NM	Rod 40 nm	Diffusion 37 °C 24 h pH NM Inoculum size NM No control	*E. coli* *S. aureus* *P. aeruginosa*	9 9 10 mm	[255]
*Sida acuta*	Leaves	Copper (II) sulphate 1000 mM/plant extract 4% (2:1 *v*/*v*) 100 °C 5–7 h pH NM	Spherical 50 nm	Diffusion 37 °C 24 h pH NM Inoculum size NM No control	*E. coli* *Proteus vulgaris*	15 11 mm	[256]
*Prosopis cineraria*	Leaves	Copper (I) acetate 5 mM/plant extract 10% (1:1 *v*/*v*) Room temperature 24 h pH NM	Hexagonal 19–32 nm	Diffusion 37 °C 24 h pH NM Inoculum size NM Cefotaxim **	*Proteus vulgaris* *P. aeruginosa* *K. pneumoniae* *E. coli* *S. aureus* *S. epidermidis*	17 18 22 22 19 23 mm	[257]
*Syzygium aromaticum*	Buds	Copper (II) acetate 1 mM/plant extract 100% (5:1 *v*/*v*) 30 °C 15 min pH NM	Spherical 20 nm	Diffusion 37 °C 24 h pH NM Inoculum size NM No control	*Staphylococcus* spp. *E. coli* *Pseudomonas* spp. *Bacillus* spp.	5 6 7 8 mm	[258]
				Diffusion 37 °C 72 h pH NM Inoculum size NM No control	*A. niger* *A. flavus* *Penicillium* spp.	5 5 6 mm	
*Ruellia tuberosa*	Leaves	Copper (II) sulfate 1 mM/plant extract 5% (1:1 *v*/*v*) 100 °C 7–8 h pH NM	Nanorod 20–100 nm	Diffusion 37 °C 24 h pH NM Inoculum size NM Streptomycin **	*S. aureus* *K. pneumoniae* *E. coli*	13 14 18 mm	[259]
*Punica granatum*	Peels	Copper (II) sulfate 50 mM/plant extract 10% (1:1 *v*/*v*) 80 °C 10 min pH NM	Spherical 15–20 nm	Diffusion 37 °C 24 h pH NM Inoculum size NM Streptomycin **	*P. aeruginosa* MTCC 424 *Salmonella enterica* MTCC 1253 *Micrococcus luteus* MTCC 1809 *Enterobactera erogenes* MTCC 2823	19 20 20 19 mm	[260]
*Asparagus adscendens*	Leaves	Copper (II) sulfate 1 mM/plant extract 5% (10:1 *v*/*v*) 100 °C 1 h pH NM	Spherical 10–15 nm	Diffusion 37 °C 24 h pH NM 1 × 10^8^ CFU/mL Ampicillin 25 µg/mL	*E. coli* *B. subtilis* *S. typhi* *K. pneumoniae* *S. aureus*	20 18 21 18 17 mm	[261]
*Gloriosa superba*	Leaves	Copper (II) sulfate 1 mM/plant extract 5% (4:1 *v*/*v*) 60 °C 3–4 min pH NM	Spherical 5–10 nm	Diffusion 37 °C 24–36 h pH NM Inoculum size NM Ciprofloxacin 0.5 µg/µL	*Klebsiella aerogenes* NCIM 2098 *E. coli* NCIM 5051 *S. aureus* NCIM 5022 *Pseudomonas desmolyticum* NCIM 2028	15 13 6 5 mm	[262]
*Cassia auriculata*	Leaves	Copper (II) sulfate1 mM/plant extract 5% (4:1 *v*/*v*) Room temperature 5 h pH NM	Clusters 38 nm	Diffusion 37 °C 24 h pH NM 1 × 10^8^ CFU/mL Amoxicillin **	*E. coli* *P. aeruginosa* *S. aureus* *Proteus mirabilis* *Bacillus cereus* *K. pneumoniae*	16 10 14 16 18 14 mm	[263]
*Bersama abyssinica*	Leaves	Copper (I) acetate 100 mM/plant extract 10% (1:1 *v*/*v*) 80 °C 2 h pH NM	Spherical 31 nm	Diffusion 37 °C 24 h pH NM Inoculum size NM Gentamicin **	*S. aureus* *B. subtilis* *E. coli* *P. aeruginosa*	12 6 14 6 mm	[264]
*Datura innoxia*	Leaves	Copper (II) sulfate 1 mM/plant extract 5% (1:1 *v*/*v*) 100 °C 1 h pH NM	Nanoclusters 90–200 nm	Diffusion 37 °C 24 h pH NM Inoculum size NM Plantomycin *	*Xanthomos oryzae pv. oryzae*	24 mm	[265]
*Zingiber officinale*	Rhizome	Copper (II) sulfate 5 mM/plant extract 30% (5:3 *v*/*v*) 60 °C 4 h pH NM	Spherical 31 nm	Diffusion 37 °C 24 h pH NM Inoculum size NM Ciprofloxacin **	*E. coli*	22 mm	[266]
*Vaccinium arctostaphylos*	Fruit	Copper (II) acetate/plant extract 5% (1:20 *w*/*v*) 60 °C 24 h pH 10	Spherical 14 nm	Diffusion 37 °C 24 h pH NM Inoculum size NM Nitrofurantoïn **	*E. coli*	22 mm	[267]
*Cissus arnotiana*	Leaves	Copper (II) sulphate 10 mM/plant extract 1% (9:1 *v*/*v*) 60 °C 4 h pH NM	Spherical 60–90 nm	Diffusion 37 °C 18 h pH NM Inoculum size NM Ampicillin **	*E. coli* *Streptococcus* spp. *Rhizobium* spp. *Klebsiella* spp.	22 20.2 16.3 18.3 mm	[268]

* MIC = minimal inhibition concentration; ZOI = zone of inhibition; PI = percentage of inhibition. ** The quantity or concentration is not mentioned. NM = not mentioned; MRSA = methicillin-resistant *S. aureus*; MSSA = methicillin-susceptible *S. aureus.*

**Table 8:** Antimicrobial green-synthesized iron nanoparticles.

Plant Type	Part Used	Operative Conditions for Synthesis	NP Characteristics (Shape and Size)	Microbiological Analyzes (Operative Conditions)			Refs.
				Methods, Incubation Temperature, Incubation Time, pH, Inoculum Density, Positive Control	Tested Bacteria and Fungi	MIC, DOI or PI *	
*Withania coagulans*	Berries	Iron (III) chloride 2000 mM/plant extract 12% (5:1 *v*/*v*) 90 °C 30 min pH NM	Rod 16 nm	Diffusion 37 °C 24 h pH NM Inoculum size NM No control	*P. aeruginosa* *S. aureus*	24 23 mm	[269]
*Acacia nilotica*	Pods	Iron (II) sulfate 100 mM/plant extract 10% (3:2 *v*/*v*) Room temperature 24 h pH 6	Irregular 39 nm	Diffusion 30 °C 24 h pH NM Inoculum size NM No control	*E. coli* MRSA *S. typhi* *S. aureus* *C. albicans*	17 24 23 25 25 mm	[270]
*Musa ornate*	Flowers	Iron (III) chloride 1 mM/plant extract 10% (1:1 *v*/*v*) 70–80 °C 1 h pH NM	Spherical 20–40 nm	Diffusion 37 °C 24 h pH NM Inoculum size NM No control	*Streptococcus agalactiae* *S. aureus* *Salmonella enterica* *E. coli*	28 32 0 0 mm	[271]
*Skimmia laureola*	Leaves	Iron (III) chloride 100 mM/plant extract 10% (1:1 *v*/*v*) Room temperature 30 min pH NM	Spherical 56–350 nm	Diffusion 37 °C 24 h pH NM Inoculum size NM Streptomycin 200 ppm	*Ralstornia solanacearum*	18 mm	[272]
*Lagenaria siceraria*	Leaves	Iron (III) chloride 10 mM/plant extract 5% (1:1 *v*/*v*) 40 °C 60 min pH NM	Cubic 30–100 nm	Diffusion 37 °C 24 h pH NM Inoculum size NM Ampicillin 20 mg/mL	*S. aureus* *E. coli*	14 17 mm	[273]
*Trigonella foenum-graecum*	Seed	Iron (II) chloride 1000 mM/plant extract 5% (1:2 *v*/*v*) Room temperature 2 h pH 10	Spherical ∼20 nm	Diffusion 37 °C 24 h pH NM Inoculum size NM No control	*E. coli* ATCC 11775 *S. aureus* ATCC 6538	22 24 mm	[148]
*Dodonaea vicosa*	Leaves	Iron (III) chloride 10 mM/plant extract 20% (2:1 *v*/*v*) 50 °C 1 h pH NM	Spherical 50–60 nm	Diffusion 37 °C 24 h pH NM Inoculum size NM No control	*E. coli* MTCC 443 *K. pneumoniae* NCIM 2079 *B. subtilis* MTCC 441 *S. aureus* MTCC 4032 *Pseudomonas fluorescens* MTCC 121	8 10 12 14 24 mm	[274]
*Couroupita guianensis*	Peel	Iron (III) chloride 100 mM/plant extract 5% (1:1 *v*/*v*) 80 °C 30 min pH 10	Spherical 7–80 nm	Diffusion 37 °C 24 h pH NM 1 × 10^5^ CFU/mL Streptomycin 1 mg	*S. aureus* MTCC 96 *E. coli* MTCC 2939 *S. typhi* MTCC 3917 *K. pneumoniae* MTCC 530	8 15 15 12 mm	[275]
*Psidium guajava*	Fruit	Iron (III) chloride 500 mM/plant extract 4% (4:1 *v*/*v*) 100 °C 1 h pH NM	Hexagonal 27 nm	Diffusion 37 °C 18–24 h pH NM Inoculum size NM Gentamicin 10 μg	*Bacillus cereus* *E. coli* *K. pneumoniae* *S. aureus*	14 17 10 14 mm	[276]
*Punica granatum*	Peel	Iron (III) chloride 150 mM/plant extract 4.6% (5:2 *v*/*v*) 20 °C 5 h pH NM	Spherical 20–90 nm	Diffusion 30 °C 24 h pH NM Inoculum size NM Streptomycin **	*P. aeruginosa*	22 mm	[277]
*Argemone mexicana*	Leaves	Iron (III) chloride 25 mM/plant extract 3% (1:1 *v*/*v*) 45 °C 12 h pH NM	Spherical 10–30 nm	Diffusion 37 °C 24 h pH NM 1 × 10^6^ CFU/mL Streptomycin 30 µg	*E. coli* MTCC 443 *B. subtilis* MTCC 425 *Proteus mirabilis* MTCC 441	13 18 10 mm	[278]
*Ruellia tuberosa*	Leaves	Iron (II) sulphate 1000 mM/plant extract 5% (1:1 *v*/*v*) 80 °C 30 min pH NM	Hexagonal 53 nm	Diffusion 37 °C 24 h pH NM 1 × 10^6^ CFU/mL Streptomycin **	*K.pneumoniae* *E. coli* *S.aureus*	13 16 11 mm	[279]
*Leucas aspera*	Leaves	Iron (III) chloride 5 mM/plant extract 20% (1:1 *v*/*v*) 80 °C 15 min pH NM	Irregular rhombic 117 nm	Diffusion 37 °C 24 h pH NM Inoculum size NM Ampicillin 10 µg	*E. coli* *K. pneumoniae* *Proteus mirabilis* *Salmonella enterica* *Shigella flexneri* *Vibrio cholera* *P. aeruginosa* *Bacillus cereus* *S. aureus* *Listeria monocytogens*	10 10 11 19 22 10 20 00 11 12 mm	[280]
*Eichhornia crassipes*	Leaves	Ferrous sulphate 100 mM/plant extract 5% (1:1 *v*/*v*) 55 °C 2 h pH 10	Rod 10–100 nm	Diffusion 37 °C 24 h pH NM Inoculum size NM No control	*S. aureus* *Pseudomonas fluorescens* *E. coli*	23 23 20 mm	[281]
*Sida cordifolia*	Whole the plant	Iron nitrate 10 mM/plant extract 5% (2:1 *v*/*v*) 60 °C 5 min pH NM	Spherical 10–22 nm	Diffusion 37 °C 24 h pH NM Inoculum size NM No control	*B. subtilis* *S. aureus* *E. coli* *K. pneumoniae*	17 15 15 19 mm	[282]
*Trigonella foenum-graecum*	Seed	Iron (III) chloride 10 mM/plant extract 0.04% (1:20 *v*/*v*) 30 °C 15 min pH NM	Spherical 11 nm	Diffusion 37 °C 24 h pH NM Inoculum size NM Streptomycin **	*E. coli* *S. aureus*	22 19 mm	[283]
*Piliostigma thonningii*	Flowers	Iron (II) chloride 1 mM/plant extract 20% (9:1 *v*/*v*) 80 °C 2 min pH NM	Spherical 20–100 nm	Diffusion 37 °C 24 h pH NM Inoculum size NM Gentamycin **	*E. coli* *S. aureus*	21 20 mm	[284]

* MIC = minimal inhibition concentration; ZOI = zone of inhibition; PI = percentage of inhibition. ** The quantity or concentration is not mentioned. NM = not mentioned; MRSA = methicillin-resistant *S. aureus.*

**Table 9:** Experimental conditions for antimicrobial susceptibility testing methods as recommended by CLSI (Adapted from [303,318,365,368,369,370,371,372]).

Methods	Microorganism	Growth Medium	Final Inoculum Size	Incubation Temperature	Incubation Time ^1^
Disk diffusion	Bacteria	MHA	1–2 × 10^8^ CFU/mL	35 ± 2 °C	16–18 h
	Fungi	MHA + GMB (Yeast)	1–5 × 10^6^ CFU/mL (yeast)	35 ± 2 °C	20–24 h
		Non-supplemented MHA (molds)	0.4–5 × 10^6^ CFU/mL (molds)	–	–
Broth dilution	Bacteria	MHB	5 × 10^5^ CFU/mL	35 ± 2 °C	20 h ^2^
	Fungi	RPMI 1640 (yeast)	0.5–2.5 × 10^3^ CFU/mL (yeast)	35 °C	24–48 h (yeast) ^3^
		RPMI 1640 (molds)	0.4–5 × 10^4^ CFU/mL (molds)	35 °C	48 h (molds) ^4^
Agar dilution	Bacteria	MHA	1 × 10^4^ CFU/spot	35 ± 2 °C	16–20 h
Time-kill test	Bacteria	MHB	5 × 10^5^ CFU/mL	35 ± 2 °C	0, 4, 18 and 24 h

^1^ MHA: Mueller–Hinton agar; MHB: Mueller–Hinton broth; GMB: glucose (2%) and methylene blue (0.5 mg/mL); RPMI 1640: Roswell Park Memorial Institute medium. ^2^ The USP recommends incubating *Escherichia coli*, *Pseudomonas aeruginosa* and *Staphylococus aureus* at 32.5 ± 2.5 °C for 18–24 h. In contrast, *Candida albicans* should be incubated at 22.5 ± 2.5 °C for 44–52 h, and *Aspergillus niger* at 22.5 ± 2.5 °C for 6–10 days. ^3^ 24–48 h for microdilution and 46–50 h for macrodilution.^4^ 48 h for both microdilution and macrodilution.

## Data Availability

The data presented in this study are available on request from the corresponding authors.

## Supplementary Materials

The following supporting information can be downloaded at: https://www.mdpi.com/article/10.3390/nano12111841/s1, Table S1. Green silver nanoparticles exhibiting antimicrobial activity; Table S2. Green gold nanoparticles exhibiting antibacterial and antifungal activities; Table S3. Antimicrobial green synthesized zinc (oxide) nanoparticles; Table S4. Green platinum nanoparticles exhibiting antimicrobial activity; Table S5. Green palladium nanoparticles with antimicrobial activity; Table S6. Green copper nanoparticles exhibiting antimicrobial activity; Table S7. Antimicrobial green-synthesized iron nanoparticles [465,466,467,468,469,470,471,472,473,474,475,476,477,478,479,480,481,482,483,484,485,486,487,488,489,490,491,492,493,494,495,496,497,498,499,500,501,502,503,504,505,506,507,508,509,510,511,512,513,514,515,516,517,518,519,520,521,522,523,524,525,526,527,528,529,530,531,532,533,534,535,536,537,538,539,540,541,542,543,544,545,546,547,548,549,550,551,552,553,554,555,556,557,558,559,560,561,562,563,564,565,566,567,568,569,570,571,572,573,574,575,576,577,578,579,580,581,582,583,584,585,586,587,588,589,590,591,592,593,594,595,596,597,598,599,600,601,602,603,604,605,606,607,608,609,610,611,612,613,614,615,616,617,618,619,620,621,622,623,624,625,626,627,628,629,630,631,632,633,634,635,636,637,638,639,640,641,642,643,644,645,646,647,648,649,650,651,652,653,654,655,656,657,658,659,660,661,662,663,664,665,666,667,668,669,670,671,672,673,674,675,676,677,678,679,680,681,682,683,684,685,686,687,688,689,690,691,692,693,694,695,696,697,698,699,700,701,702,703,704,705,706,707,708,709,710,711,712,713,714,715,716,717,718,719,720,721,722,723,724,725,726,727,728,729,730,731,732,733,734,735,736,737,738,739,740,741,742,743,744,745,746,747,748,749,750,751,752,753,754,755,756,757,758,759,760,761,762,763,764,765,766,767,768,769,770,771,772,773,774,775,776,777,778,779,780,781,782,783,784,785,786,787,788,789,790,791,792,793,794,795,796,797,798,799,800,801,802,803,804,805,806,807,808,809,810,811,812,813,814,815,816,817,818,819,820,821,822,823,824,825,826,827,828,829,830,831,832,833,834,835,836,837,838,839,840,841,842,843,844,845,846,847,848,849,850,851,852,853,854].
