# Supplementary material for: A Critical Review of the Antimicrobial and Antibiofilm Activities of Green-Synthesized Plant-Based Metallic Nanoparticles
Source: Nanomaterials (Basel). 2022 May 27;12(11):1841. doi: 10.3390/nano12111841 (PMC9182092; doi:10.3390/nano12111841)
Supplement: Supplementary file 1 [file nanomaterials-12-01841-s001.zip › nanomaterials-1627847-supplementary.pdf]

Table S1. Green silver nanoparticles exhibiting antimicrobial activity

**Plant type**	**Part used**	**Operative conditions for synthesis**	**NP characteristics (Shape and size)**	**Microiological analyses (operative conditions)**			**Ref.**
				**Methods,<br>incubation temperature,<br>incubation time,<br>pH,<br>inoculum density,<br>positive control**	**Tested bacteria and fungi**	**MIC, ZOI or PI***	
Piper longum	Catkin	Silver nitrate 1 mM/<br>plant extract 10 %<br>(19:1 v/v)<br>95°C<br>Incubation time NM<br>pH NM	Spherical<br>10–42 nm	Diffusion<br>37°C<br>24 h<br>pH NM<br>Inoculum NM<br>No control	*B. cereus* MTCC 1272<br>*E. coli* MTCC 1687<br>*K. pneumoniae* MTCC 530<br>*P. mirabilis* MTCC 425<br>*P. aeruginosa* MTCC 1688<br>*S. typhi* MTCC 531<br>*S. aureus*MTCC 96	12<br>13<br>14<br>15<br>11<br>12<br>11 mm	[465]
Carissa carandas	Leaves	Silver nitrate 1 mM/<br>plant extract 10 % (9:1 v/v)<br>60°C<br>1 h<br>pH 7.2	Spherical<br>30 nm	Diffusion<br>37°C<br>24 h<br>pH NM<br>$1×10^8$ CFU/ml<br>No control	*S. typhi*<br>*Enterococcus faecalis*<br>*Shigella flexneri*<br>*Citrobacter spp*<br>*Gonococci spp*	12<br>16<br>24<br>14<br>21 mm	[466]
S*olanum* tricobatum	Leaves	Silver nitrate 1 mM/<br>plant extract 1.5 %<br>(1:10 v/v)<br>37°C<br>48 h<br>pH NM	Irregular<br>26.5 nm	Diffusion<br>35°C<br>18 h<br>pH NM<br>Inoculum NM<br>No control	*S. aureus*<br>*P. aeruginosa*<br>*E. coli*<br>*K. pneumoniae*	30<br>12<br>14<br>18 mm	[467]
Cleome viscosa	Fruit	Silver nitrate 1 mM/<br>plant extract 10 %<br>(10:1 v/v)<br>25 ± 2°C<br>24 h	Spherical<br>5–30 nm	Diffusion<br>37°C<br>18 h<br>pH NM<br>$1×10^7$ CFU/ml	*B. subtilis*<br>*S. aureus*<br>*E. coli*<br>*K. pneumoniae*	12<br>13<br>13<br>11 mm	[468]

		pH NM		Tétracycline**			
Musa paradisiaca	Peels	Silver nitrate 1.75 mM/ plant extract 1 % (5:1 v/v)<br>40–100°C<br>72 h<br>pH 4.5	Spherical<br>23.7 nm	Diffusion<br>37°C<br>24 h<br>pH NM<br>1×10^8 CFU/ml<br>Levofloxacin**	*B. subtilis*<br>*S. aureus* ATCC 6538<br>*P. aeruginosa* ATCC 9027<br>*P. aeruginosa isolated*<br>*E. coli* ATCC 8739	12<br>16<br>20<br>18<br>17 mm	[469]
Azadirachta indica	Leaves	Silver nitrate 1 mM/ plant extract 10 % (10:1 v/v)<br>Room temperature<br>Incubation time NM<br>pH NM	Spherical<br>5–20 nm	Diffusion<br>37°C<br>24 h<br>pH NM<br>Inoculum NM<br>No control	*S. aureus*<br>*E. coli*	9<br>9 mm	[470]
Centella asiatica	Leaves	Silver nitrate 1 mM/ plant extract 1.5 % (1:2 v/v)<br>37°C<br>24–48h<br>pH NM	Irregular<br>42 nm	Diffusion<br>35 °C<br>18 h<br>pH NM<br>Inoculum NM<br>No control	*S. aureus*<br>*P. aeruginosa*<br>*K. pneumoniae*<br>*E. coli*	15<br>16<br>0 mm<br>12	[471]
Citrus sinensis			Irregular<br>41 nm		*S. aureus*<br>*P. aeruginosa*<br>*K. pneumoniae*<br>*E. coli*	12<br>16<br>11<br>12 mm	
Rheum ribes	Leaves	Silver nitrate 1 mM/ plant extract 5 % (10:1 w/v)<br>75°C<br>48 h<br>pH NM	Spherical<br>18.2 ± 3.6 nm	Diffusion<br>37 °C<br>24 h<br>pH NM<br>1×10^8 CFU/ml<br>No control	*E. coli* ATCC 8739<br>*S. aureus* ATCC 6538<br>MRSA ATCC43300<br>*B. subtilis* ATCC6633	15<br>17<br>17<br>19 mm	[472]
Sisymbrium irio	Leaves	Silver nitrate 1 mM/ plant extract 10 % (9:1 v/v)<br>60 °C	Spherical<br>45 nm	Diffusion<br>37°C<br>24 h<br>pH NM	*P. aeruginosa*<br>*Acinetobacter baumannii*<br>*E. coli* ATCC 25922	20<br>18<br>6 mm	[473]

		24 h<br>NM		1×10^6 CFU/ ml<br>Meropenem 10 µg			
Tephrosia apollinea	Seeds	NM<br>NM<br>NM<br>NM	Spherical and cubic<br>300–700 nm	Dilution<br>37°C<br>24 h<br>pH NM<br>$1–2\times10^8$CFU/ml<br>No control	*E. coli* ATCC 25922<br>*S. aureus* ATCC 29213	31.25<br>15.625<br>µg/ml	[474]
Picea abies	Bark	Silver acetate 1 mM / plant extract 10 % (9:1 v/v)<br>60°C<br>3 h<br>pH 4	Spherical<br>165.5 nm	Dilution<br>Temperature NM<br>Incubation time NM<br>pH NM<br>Inoculum NM<br>Ciprofloxacin**	*S. aureus* ATCC 25923<br>*S. aureus* ATCC 43300<br>*E. coli* ATCC 25922<br>*P. aeruginosa* ATCC 27853<br>*K. pneumoniae* ATCC 700603	1,24<br>1.86<br>>1.86<br>>1.86<br>1.86 mg/ml	[475]
Aesculus hippocastanum	Leaves	Silver nitrate 5 mM/ plant extract 20 % (3:1 v/v)<br>95 °C<br>10 min<br>pH NM	Spherical<br>50 ± 5 nm	Diffusion<br>37°C<br>18 –24h<br>pH NM<br>1×10^8 CFU/ml<br>Ampicillin 10 µg	*S. aureus* ATCC 25923<br>*S. epidermidis* ATCC 12228<br>*Micrococcus luteus* ATCC 10240<br>*B. subtilis* ATCC 29213<br>*B. cereus* NRLL B-3008<br>*E. faecalis* ATCC 292112<br>*P. aeruginosa* ATCC 27853<br>*E. coli* ATCC 25922<br>*E. aerogenes* ATCC 13048<br>*K. pneumonia* ATCC 13883<br>*P. mirabilis* ATCC 25933<br>*Listeria monocytogenes* ATCC 19115<br>*Corynebacterium renale* ATCC 19412<br>*Pseudomonas fluorescens* ATCC 49838	18<br>13<br>17<br>15<br>15<br>11<br>20<br>10<br>11<br>13<br>10<br>13<br>12<br>8 mm	[476]

Galega officinalis	Leaves	Silver nitrate 1.6 mM/ plant extract 15 % (1:1 v/v)<br>Room temperature<br>24 h<br>pH 11	Spherical<br>8–34 nm	Dilution<br>37°C<br>24 h<br>pH NM<br>2×10^6 CFU/ml<br>Streptomycin**<br>Gentamycin**	*E. coli*<br>*Pseudomonas syringae*<br>*S. aureus*	5<br>10<br>50 µg/ml	[477]
Alpinia nigra	Fruits	Silver nitrate 2 mM/ plant extract 10 % (6.25 :1 v/v)<br>Room temperature<br>2 h<br>pH NM	Spherical<br>65.9 nm	Diffusion<br>37°C<br>16–18 h<br>pH NM<br>1×10^8 CFU/ml<br>Neomycin 1 mg/ml	*S. aureus* ATCC 11682<br>*K. pneumoniae* ATCC 13883	18<br>20 mm	[478]
				Diffusion<br>30°C<br>24-48h<br>pH NM<br>1×10^8 CFU/ml<br>Nystatin 2mg/ml	*C. albicans* MTCC 3017	12 mm	
Impatiens balsamina	Leaves	Silver nitrate 10 mM/ plant extract 10 % (1:1 v/v)<br>60°C<br>5h<br>pH NM	Spherical<br>12 –23.3 nm	Diffusion<br>37°C<br>24 h<br>pH NM<br>1×10^8 CFU/ml<br>Ciprofloxacin**	*S. aureus*<br>*E. coli*	11<br>10 mm	[479]
Lantana camara			Spherical<br>3.2–14.1 nm		*S. aureus*<br>*E. coli*	14<br>18 mm	
Juniperus procera	Leaves	Silver nitrate 1 mM / plant extract 20 % (9:1 v/v)<br>Room temperature<br>24h<br>pH NM	Spherical and cubic<br>30–90 nm	Diffusion<br>32°C<br>18 h<br>pH NM<br>Inoculum NM<br>Penicillin 10 µg	*B. subtilis*<br>*Micrococcus luteus*<br>*Proteus mirabilis*<br>*K. pneumoniae*<br>*C. albicans*	28<br>28<br>29<br>18<br>24 mm	[480]

Acalypha wilkesiana	Leaves	Silver nitrate 1 mM/ plant extract 10 % (9:1 v/v)<br>90–100°C<br>90 min<br>pH 9	Spherical<br>10–26 nm	Diffusion<br>37°C<br>24 h<br>pH 9<br>1.5×10^{8} CFU/ml<br>Chloramphenicol**	*E. coli*<br>*S. aureus*	17<br>19 mm	[481]
Selaginella bryopteris	Leaves	Silver nitrate 1 mM/ plant extract 25 %<br>(10:1 v/v)<br>80°C<br>30 min<br>pH NM	Spherical<br>5–10 nm	Diffusion<br>37 °C<br>24–48 h.<br>pH ±7<br>Inoculum NM<br>Ciproflaxin**	*E. coli*<br>*S. aureus*	12<br>11 mm	[482]
				Diffusion<br>25°C<br>48–72 h<br>pH 6.8<br>Inoculum size NM<br>Amphotericin B**	*A. niger*	2 mm	
Camellia sinensis	Leaves	Silver nitrate 10 mM / plant extract 15 %<br>(30:1 v/v)<br>30°C<br>10 min<br>pH NM	Spherical<br>30–40 nm	Diffusion<br>37°C<br>24 h<br>pH NM<br>1×10^{6} CFU/ml<br>No control	*E. coli* MTCC 443<br>*S. aureus* MTCC 10536<br>*S. pyogenes* MTCC 442	14<br>13<br>10 MM	[483]
Citrus sinensis	Pericarp	Silver nitrate 50 mM/ plant extract 0.3 %<br>(1:1 v/v)<br>60°C<br>2 h<br>pH NM	Spherical<br>107.1 ± 2.6 nm	Dilution<br>37°C<br>24 h<br>pH NM<br>1×10^{8} CFU/ml<br>Gentamycin**	*E. coli*<br>MRSA	0.5<br>0.5<br>0.5 µg/ml	[484]

Artemisia marschalliana	Aerial part	Silver nitrate 1 mM/ plant extract 10 % (25 :1 v/v)<br>Room temperature<br>5 min<br>pH NM	Spherical<br>5–20 nm	Diffusion<br>37°C<br>24 h<br>pH NM<br>$1×10^7$ CFU/ml<br>Ampicillin**	*B. cereus* ATCC 14579<br>*S. aureus* ATCC 6538<br>*P. aeruginosa* ATCC 15442<br>*Acinetobacter baumannii* ATCC 19606	9<br>16<br>13<br>11 mm	[485]
Gleichenia pectinata	Fern	Silver nitrate 5 mM/ plant extract 10 % (5:1 v/v)<br>Room temperature<br>30 min<br>pH NM	Spherical<br>7.5 ± 2.9 nm	Diffusion<br>Temperature NM<br>Incubation NM<br>pH NM<br>Inoculum NM<br>Streptomycin**	*P. aeruginosa*<br>*E. coli*<br>*K. pneumoniae*	15<br>11<br>10 mm	[486]
				Diffusion<br>Temperature NM<br>Incubation time NM<br>pH NM<br>Inoculum size NM<br>Nystatin**	*C. albicans*	12 mm	
Cymbopogon citratus	Leaves and raw fruits	Silver nitrate 1 mM / plant extract 10 % (9:1 v/v)<br>37°C<br>24 h<br>pH NM	Spherical<br>20–40 nm	Diffusion<br>37°C<br>24 h<br>pH NM<br>$1.5×10^6$ CFU/ml<br>No control	*B. cereus* ATCC BAA512<br>*E. coli* ATCC 11775<br>*P. aeruginosa* ATCC 19429<br>*Bacillus licheniformis* D5MZ-8059	12<br>16<br>18<br>10 mm	[487]
Rhus coriaria	Leaves	Silver nitrate 1 mM/ plant extract 5 % (1:1 v/v)<br>25°C<br>2 h<br>pH NM	Spherical and tetragonal<br>22.4–37.6 nm	Diffusion<br>37°C<br>24 h<br>pH NM<br>$1×10^6$ CFU/ml<br>No control	*P. vulgaris*<br>*S. aureus*<br>*Erwinia carotovora*<br>*B. subtilis*<br>*K. pneumoniae*	8<br>8<br>11<br>10<br>10 mm	[488]
Carthamus tinctorius	Leaves		Spherical and tetragonal<br>14.5–35.8 nm		*P. vulgaris*<br>*S. aureus*<br>*Erwina carotovora*	14<br>14<br>11	

					B. subtilis<br>*K. pneumoniae*	9<br>15 mm	
Verbena officinalis	Leaves	Silver nitrate 2 mM / plant extract 10 % (10:1 v/v)<br>37°C<br>24 h<br>pH NM	Spherical<br>42.57 ± 5.34 nm	Diffusion<br>37°C<br>24 h<br>pH NM<br>1.5×10^6 CFU/ml<br>No control	*Listeria monocytogenes*<br>*Yersinia ruckeri*<br>*Vibrio cholera*	15<br>16<br>13 mm	[489]
Vaccinium macrocarpon	NM	Silver nitrate 10 mM / plant extract 0.8 % (10:1 v/v)<br>30°C<br>24 h<br>pH 6	Spherical<br>1.4–11.1 nm	Dilution<br>37°C<br>24 h<br>pH NM<br>Inoculum size NM<br>No control	*S. aureus*<br>*P. aeruginosa*<br>MRSA	39.5<br>25.3<br>39.5 µg/ml	[490]
Garcinia quaesita.	Fruits	Silver nitrate 1 mM/ plant extract 1 % (10:1 v/v)<br>100°C<br>Room temperature<br>24 h<br>pH NM	Spherical<br>7–22 nm	Diffusion<br>37°C<br>24 h<br>pH NM<br>1.5×10^8 CFU/ml<br>Vancomycin 10 mg/ml<br>Gentamycin 10 mg/ml	*S. aureus*<br>*P. aeruginosa*<br>*E. coli*<br>MRSA<br>*C. albicans* ATCC 10231	16<br>18<br>16<br>22<br>21 mm	[491]
Arisaema flavum	Tubers	Silver nitrate**/ plant extract 5 % (ratio NM)<br>Room temperature<br>4 h<br>pH NM	Spherical<br>5–8 nm	Diffusion<br>37 °C<br>18–20 h<br>pH NM<br>Inoculum size NM<br>Streptomycin 1 mg/ml	*E. coli*<br>*B. subtilis* BL-21<br>*S. aureus*<br>*Pseudomonas putida*<br>*E. coli* substrain QH4	16<br>18<br>23<br>12<br>11 mm	[492]
Olea europaea	Leaves	Silver nitrate 1 mM / plant extract 1.5 % (2:3 v/v)<br>37 °C<br>30 min	Spherical<br>45 ± 2 nm	Dilution<br>37°C<br>24 h<br>pH NM<br>1×10^6 CFU/ml	*E. coli*<br>*S. enterica*<br>*S. aureus*	9.38<br>18.75<br>9.38 µg/ml	[493]
Rosmarinus officinalis			Spherical		*E. coli*	4.69	

		pH NM	38 ± 3 nm	No control	*S. enterica*<br>*S. aureus*	18.75<br>4.69 µg/ml	
Bergenia ciliate	Roots	Silver nitrate 1 mM / plant extract 4 % (9:1 v/v)<br>37 °C<br>24 h<br>pH NM	Spherical<br>25 nm	Diffusion<br>37 °C<br>12–16 h<br>pH NM<br>1×10^7–10^8 CFU/ml<br>Streptomycin**	*S. aureus* KX 262679<br>*B. cereus* KX 262674<br>*E. coli* ATCC 10536<br>*S. typhi* ATCC 6539<br>*P. aeruginosa* ATCC 9027<br>*Staphylococcus haemolyticus* KX262673	13<br>0<br>0<br>13<br>15<br>0 mm	[494]
Bergenia stracheyi			Spherical<br>73 nm		*S. aureus* KX 262679<br>*B. cereus* KX 262674<br>*E. coli* ATCC 10536<br>*S. typhi* ATCC 6539<br>*P. aeruginosa* ATCC 9027<br>*Staphylococcus haemolyticus* KX 262673	13<br>0<br>0<br>13<br>16<br>0 mm	
Rumex dantatus			Spherical<br>50 nm		*S. aureus* KX 262679<br>*B. cereus* KX 262674<br>*E. coli* ATCC 10536<br>*S. typhi* ATCC 6539<br>*P. aeruginosa* ATCC 9027<br>*Staphylococcus haemolyticus* KX 262673	16<br>0<br>0<br>12<br>14<br>16<br>0 mm	
Rumex hastatus			Spherical<br>48 nm		*S. aureus* KX 262679<br>*B. cereus* KX 262674<br>*E. coli* ATCC 10536<br>*S. typhi* ATCC 6539<br>*P. aeruginosa* ATCC 9027<br>*Staphylococcus haemolyticus* KX 262673	15<br>8<br>10<br>18<br>17<br>9 mm	

Bougainvillea spectabilis	Flowers	Silver nitrate 1 mM/ plant extract 5 % (9:1 v/v)<br>Room temperature<br>24 h<br>pH NM	Spherical<br>16–83 nm	Diffusion<br>37°C<br>24 h<br>pH NM<br>Inoculum size NM<br>No control	*S. typhi*<br>*S. aureus*<br>*B. subtilis*<br>*B. cereus*<br>*Enterococcus faecalis*<br>*Corynebacterium diphtheria*<br>*Streptococcus pneumoniae*<br>*K. pneumoniae*<br>*E. coli*<br>*Enterobacter aerogenes*<br>*P. aeruginosa*	4<br>8<br>15<br>9<br>6<br>6<br>9<br>3<br>7<br>10<br>3 mm	[495]
Bauhinia acuminate	Leaves	Silver nitrate 1 mM/ plant extract 10 % (10:1 v/v)<br>Room temperature<br>24 h<br>pH NM	Irregular<br>78–110 nm	Dilution<br>37° C<br>24 h<br>pH NM<br>1×10^5 CFU/ml<br>Ciproflaxin**	*E. coli*<br>*S. aureus*	900<br>750 µg/ml	[496]
Prosopis juliflora	Leaves	Silver nitrate 2 mM/ plant extract 10 % (1:1 v/v)<br>Room temperature<br>24 h<br>pH NM	Spherical<br>10–20 nm	Diffusion<br>37º C<br>24 h<br>pH NM<br>10^5 CFU/ml<br>Kanamycin**	*E. coli*<br>*P. aeruginosa*	15<br>12 mm	[172]
Coriandrum sativum	Leaves	Silver nitrate 1 mM/ plant extract 10 % (4:1 v/v)<br>75°C<br>20 min<br>pH 8.6	Ball shape<br>11.9 nm	Diffusion<br>37°C<br>24 h<br>pH NM<br>Inoculum size NM<br>Gentamycin**	*Pasteurella multocida*<br>*Enterobacter aerogenes*<br>*S. aureus*<br>*B. subtilis*	10<br>11<br>12<br>14 mm	[497]
Datura metel	Leaves	Silver nitrate 1 mM/ plant extract 30 % (20:1 v/v)	Spherical<br>20 nm	Diffusion<br>28°C<br>48 h	*E. coli*<br>*Pseudomonas spp*	14<br>11 mm	[498]

		60°C<br>2 h<br>pH NM		pH NM<br>Inoculum size NM<br>No control			
Abelmoschus esculentus	Vegetable	Silver nitrate 45 mM/ plant extract 25 % (1:5 v/v)<br>Room temperature<br>12 h<br>NM	Spherical<br>30 nm	Diffusion<br>37°C<br>24 h<br>pH 7.4<br>Inoculum size NM<br>No control	*S. aureus*<br>*E. coli*	11<br>17 mm	[499]
Tithonia diversifolia	Leaves	Silver nitrate 1 mM/ plant extract 2 % (9 :1 v/v)<br>Room temperature<br>90 min<br>pH NM	Spherical<br>10–26 nm	Diffusion<br>37°C<br>24 h<br>pH NM<br>Inoculum size NM<br>Ciprofloxacin**	*E. coli*<br>*S. typhirium*<br>*Salmonella enterica*<br>*B. subtilis*	13<br>12<br>13<br>15 mm	[500]
Raphanus sativus	Roots	Silver nitrate 1 mM/ plant extract 16 % (9:1 v/v)<br>Room temperature<br>15 min<br>pH NM	Polygonal<br>30–60 nm	Diffusion<br>37°C<br>24 h<br>pH NM<br>Inoculum size NM<br>Amikacin**	*E. coli*<br>*K. pneumoniae*<br>*B. subtilis*<br>*S. aureus*	10<br>10<br>14<br>12 mm	[501]
Ribes khorasanicum	Fruits	Silver nitrate 1 mM/ plant extract 1.2 % (9:1 v/v)<br>70°C<br>1 h<br>pH NM	Spherical<br>15–45 nm	Diffusion<br>37 °C<br>24 h<br>pH NM<br>1.5×10^8 CFU/ml<br>Gentamicin**<br>Streptomycin**	*E. coli*<br>*P. aeruginosa*<br>*S. aureus*	9<br>9<br>13 mm	[502]
Withania coagulans	Leaves	Silver nitrate 1 mM/ plant extract 5 % (1:9 v/v)<br>27 °C	Spherical<br>7–32 nm	Diffusion<br>37 °C<br>24 h<br>pH NM	*E. coli*<br>*S. aureus*<br>*K. pneumoniae*	4<br>10<br>8 mm	[503]

		72 h<br>pH NM		Inoculum size NM<br>Ampicillin**			
Eucalyptus critriodora	Leaves	Silver nitrate 1 mM/ plant extract 5 % (1:1 v/v)<br>28 °C<br>16h<br>pH NM	Spherical<br>8–15 nm	Dilution<br>37 °C<br>18 h<br>pH NM<br>1×10^8 CFU/ml<br>No control	*Acinetobacter baumannii*	0.18 µg/ml	[504]
Canarium ovatum	Leaves	Silver nitrate 1.5 mM/ plant extract 10 % (1:20 v/v)<br>25°C<br>40 min<br>pH NM	Spherical<br>119.7 ± 7 nm	Diffusion<br>37°C<br>24 h<br>pH NM<br>1×10^6 CFU/ml<br>Kanamycin 10 µg	*P. aeruginosa*	15 mm	[505]
Elaeocarpus ganitrus	Beads	Silver nitrate 1 mM/ plant extract 10 % (9:1 v/v)<br>60–80°C<br>20 min<br>pH NM	Spherical<br>13.6–36.3 nm	Diffusion<br>37°C<br>24 h<br>pH NM<br>1×10^5 CFU/ml<br>Ampicillin**	*S. typhimurium* MTCC 98<br>*E. coli* MTCC 433<br>*Clostridium perfringes* MTCC 450<br>*Listeria monocytogenes* MTCC 657<br>*S. aureus* MTCC 740<br>*Vibrio mimicus* MTCC 4434	29<br>20<br>28<br>29<br>29<br>26 mm	[506]
Oryza sativa	Beads	Silver nitrate 10 mM/ plant extract 0.9 % (1:100 v/v)<br>75°C<br>60 min<br>NM	Spherical<br>80.4 ± 2.8 nm	Diffusion<br>37°C<br>24 h<br>pH NM<br>1.5×10^6 CFU/ml<br>No control	*E. coli* ATCC 2592<br>*S. aureus* ATCC 25923	13<br>8 mm	[507]
Glaucium corniculatum	Arial part	Silver nitrate 10 mM/ plant extract 8 % (8:1 v/v)<br>60°C	Spherical<br>45 nm	Diffusion<br>37°C<br>16 h<br>pH NM	*E. coli* ATCC 35218<br>*S. typhimurium* ATCC 14028<br>*S. aureus* ATCC 29213<br>*B. cereus* ATCC 14579	10<br>9<br>8<br>8 mm	[508]

		1h<br>pH NM		1×10^5 CFU/ml<br>No control			
Elettaria cardamom	Seeds	Silver nitrate 1 mM/ plant extract 40 % (9:1 v/v)<br>Room temperature<br>24 h<br>NM	Spherical<br>40–70 nm	Diffusion<br>37°C<br>24 h<br>pH NM<br>Inoculum size NM<br>No control	*B. subtilis*	14 mm	[509]
Trigonella foenum-graecum	Seeds	Silver nitrate 1 mM/ plant extract 10 % (10:1 v/v)<br>50–100°C<br>30 min<br>pH NM	Spherical<br>95–110 nm	Diffusion<br>37°C<br>24 h<br>pH NM<br>1×10^8 CFU/ml<br>Ampicillin**	*S. aureus*<br>*P. aeruginosa*<br>*Proteus vulgaris*<br>*E. coli*	17<br>12<br>6<br>9 mm	[510]
Synedrella nodiflora	Leaves	Silver nitrate 1 mM/ plant extract 25 % (9:1 v/v)<br>Room temperature<br>Incubation time NM<br>pH NM	Spherical<br>14.2 nm	Diffusion<br>37°C<br>24–48 h<br>pH NM<br>Inoculum NM<br>No control	*B. subtilis*<br>*Streptococcus spp*<br>*Pseudomonas spp*<br>*E. coli*	9<br>8<br>11<br>14 mm	[511]
				Diffusion<br>25°C<br>7 days<br>pH NM<br>Inoculum size NM<br>No control	*Aspergillus spp*<br>*Penicillium spp*	12<br>10 mm	
Cassia roxburghii	Leaves	Silver nitrate 1 mM/ plant extract 4 % (9:1 v/v)<br>Room temperature<br>24h<br>pH NM	Spherical<br>10–30 nm	Diffusion<br>37°C<br>24 h<br>pH NM<br>Inoculum NM<br>Streptomycin 10 µg	*B. subtilis*<br>*S. aureus*<br>*Micrococcus luteus*<br>*P. aeruginosa*<br>*E. coli*<br>*Enterobacter aerogenes*	17<br>19<br>12<br>21<br>18<br>16 mm	[512]

Acacia rigidula	Stems	Silver nitrate 1 mM/ plant extract 10 % (3:1 v/v)<br>60°C<br>1 h<br>pH NM	Spherical<br>8–66 nm	Dilution<br>37°C<br>20 h<br>pH NM<br>1×10^6 CFU/ml<br>No control	*E. coli* ATCC 11229<br>*P. aeruginosa* ATCC 27853<br>*P. aeruginosa* MDRS strain<br>*B. subtilis* ATCC 23857	62.5<br>15.6<br>7.8<br>0.5 µg/ml	[313]
Napier grass	Fibers	Silver nitrate 5 mM/ plant extract 5 % (1:1 v/v)<br>80°C<br>24 h<br>pH NM	Triangle, cube and spherical<br>10–100 nm	Diffusion<br>37 °C<br>48 h<br>pH 7<br>1×10^6 CFU/ml<br>No control	*E. coli*<br>*S. aureus*	32<br>33 mm	[513]
Thymus vulgaris	Leaves	Silver nitrate1 mM/plant 2 % (5:1 v/v)<br>Room temperature<br>Incubation time NM<br>pH NM	Spherical<br>95 nm	Dilution<br>37°C<br>24 h<br>pH NM<br>1×10^8 CFU/ml<br>No control	MRSA<br>*E. coli*	3.13<br>0.39 µg/ml	[514]
Malva sylvestris	Flowers	Silver nitrate 50 mM/ plant extract 15 % (1:18 v/v)<br>25°C<br>4 h<br>pH NM	Spherical<br>20–40 nm	Diffusion<br>37°C<br>24 h<br>pH NM<br>Inoculum size NM<br>No control	*E. coli*<br>*S. aureus*<br>*S. pyogenes*	10<br>9<br>9 mm	[515]
Salvadora persica	Roots	Silver nitrate 0.5 mM/ plant extract 10 % (49:1 v/v)<br>90°C<br>2 h<br>pH NM	Spherical<br>10–20 nm	Diffusion<br>37°C<br>24 h<br>pH NM<br>1×10^5 CFU/ml<br>No control	*E. coli*<br>*P. aeruginosa*<br>*S. aureus*<br>*Microccus luteus*	13<br>11<br>15<br>14 mm	[516]

Costus afer	Leaves	Silver nitrate 1 mM/ plant extract 1.3 % (5:1 v/v)<br>90°C<br>2 h<br>pH NM	Spherical<br>20 nm	Diffusion<br>37°C<br>24 h<br>pH NM<br>Inoculum NM<br>Gentamycin**	*S. aureus*<br>*E. coli*<br>*P. aeruginosa*<br>*K. pneumoniae*	23<br>21<br>18<br>14 mm	[517]
Phoenix dactylifera	Fruits	Silver nitrate 1 mM/ plant extract 10 % (9:1 v/v)<br>25°C<br>48 h<br>pH NM	Spherical<br>20–60 nm	Diffusion<br>37°C<br>18–24h<br>pH NM<br>2.5×10^5 CFU/ml<br>Ciprofloxacin**	*S. aureus*<br>*P. aeruginosa*<br>*E. coli*	18<br>12<br>15 mm	[518]
Madhuca longifolia	Flowers	Silver nitrate 1 mM/ plant extract 10 % (10:1 v/v)<br>40°C<br>20 min<br>pH NM	Spherical and oval<br>30–50 nm	Dilution<br>37°C<br>24 h<br>pH NM<br>1×10^8 CFU/ml<br>No control	*E. coli* KCTC 1682<br>*S. typhimurium* KCCM 11862<br>*B. cerues* ATCC13061<br>*Staphylococcus saprophyticus* KCTC 3345	90<br>80<br>60<br>40 µg/ml	[519]
Saussurea lappa	Roots	Silver nitrate 5 mM/ plant extract 10 % (1:1 v/v)<br>37°C<br>24 h<br>pH NM	Spherical<br>20.2 nm	Diffusion<br>30°C<br>24 h<br>pH NM<br>Inoculum size NM<br>No control	*E. coli*<br>*P. aeruginosa*	11<br>9 mm	[520]
Jurinea dolomiaea	Roots	Silver nitrate 5 mM / plant extract 20 % (1:1 v/v)<br>37°C<br>24 h<br>pH NM	Spherical, cubic and triangular<br>24.6 nm	Diffusion<br>35°C<br>24 h<br>pH NM<br>Inoculum NM<br>No control	*P. aeruginosa*<br>*E. coli*	11<br>11 mm	[521]

Gardenia jasminoides	Seeds	Silver nitrate 3 mM/ plant extract 5 % (9:1 v/v)<br>25°C<br>24 h<br>pH NM	Spherical<br>20 nm	Dilution<br>37°C<br>24 h<br>pH NM<br>1×10^9 CFU/ml<br>No control	*S. aureus* ATCC13150<br>*S. typhimurium* ATCC14028	7.5<br>10 µg/ml	[522]
Stachys lavandulifolia	Leaves	Silver nitrate 8 mM/ plant extract 10 % (2:1 v/v)<br>25°C<br>2 h<br>pH NM	Spherical<br>20–40 nm	Dilution<br>37°C<br>24 h<br>pH NM<br>Inoculum NM<br>Tetracycline**	*E. coli* ATCC 1103<br>*S. aureus* ATCC9144	43<br>16 µg/ml	[523]
Cocos nucifera	Nuts	Silver nitrate 1 mM/ plant extract 10 % (10:1 v/v)<br>25°C<br>1 h<br>pH NM	Spherical<br>14.2–22.9 nm	Diffusion<br>37°C<br>24 h<br>pH NM<br>1×10^8 CFU/ml<br>Ampicillin**	*S. aureus* ATCC 25923<br>*E. coli* ATCC 25922<br>*S. typhimurium* ATCC 13311<br>*Listeria monocytogenes* ATCC 19111	26<br>53<br>106<br>212 µg/ml	[524]
Tridax procumbens	Leaves	Silver nitrate 1 mM / plant extract 10 % (20:1 v/v)<br>25°C<br>48 h<br>pH NM	Spherical<br>20–50 nm	Diffusion<br>37 °C<br>24 h<br>pH NM<br>Inoculum NM<br>No control	*S. aureus*<br>*E. coli*<br>*P. aeruginosa*<br>*S. epidermidis*	10<br>14<br>8<br>10 mm	[525]
Reinwardtia indica	Leaves	Silver nitrate 1 mM/ plant extract 14 % (9:1 v/v)<br>38°C<br>48 h<br>pH 7	Spherical<br>3–15nm	Diffusion<br>37°C<br>24 h<br>pH NM<br>1×10^8 CFU/ml<br>No control	*E. coli* ATCC 25922<br>*S. aureus* ATCC 25923<br>*P. aeruginosa* ATCC 27853	13<br>15<br>16 mm	[526]

				Diffusion<br>25°C<br>Incubation time NM<br>pH NM<br>1×10^6 CFU/ml<br>No control	*C. albicans* ATCC 90028	14 mm	
Bergenia ciliata	Rhizome	Silver nitrate 1 mM/ plant extract 5 % (1:4 v/v)<br>100°C<br>Incubation time NM<br>pH NM	Spherical<br>35 nm	Diffusion<br>37°C<br>24 h<br>pH NM<br>Inoculum NM<br>Chloramphenicol 10 µg/ml	*E. coli*<br>*K. pneumoniae*<br>*P. aeruginosa*<br>*S. epidermidis*<br>*Serratia marcescens*<br>*S. aureus*<br>*S. pyogenes*	8<br>8<br>8<br>8<br>7<br>8<br>6 mm	[527]
Citrus sinensis	Peels	Silver nitrate 1 mM/ plant extract 6.25 % (1:1 v/v)<br>Temperature NM<br>2 h<br>pH NM	Spherical<br>48.1 ± 20.5 nm	Dilution<br>35°C<br>24 h<br>pH NM<br>1×10^8 CFU/ml<br>Ampicillin 50 µg/ml	*Xanthomonas axonopodis*	22 µg/ml	[528]
Stereospermum suaveolens	Roots	Silver nitrate 1 mM/ plant extract 5 % (9:1 v/v)<br>Temperature NM<br>Incubation time NM<br>pH NM	Spherical<br>27.2 ± 5.9 nm	Diffusion<br>37°C<br>Incubation time NM<br>pH NM<br>Inoculum NM<br>No control	*B. subtilis* MTCC 441<br>*S. aureus* MTCC 96<br>*E. coli* MTCC 443<br>*P. aeruginosa* MTCC 424	14<br>16<br>17<br>20 mm	[529]
				Diffusion<br>Temperature NM<br>Incubation time NM<br>pH NM<br>Inoculum NM<br>No control	*Aspergillus nidulans* MTCC 344<br>*A. flavus* MTCC 277	11<br>12 mm	

Wedelia chinensis	Leaves	Silver nitrate 1 mM/ plant extract 10 % (9:1 v/v)<br>60°C<br>Incubation time NM<br>pH NM	Spherical<br>18–68.8 nm	Diffusion<br>37°C<br>24 h<br>pH NM<br>Inoculum NM<br>Ampicillin**	*E. coli*<br>*Listeria monocytogenes*	17<br>12 mm	[530]
Allium sativum	Bulb	Silver nitrate 1 mM/ plant extract 20 % (3:1 v/v)<br>Room temperature<br>15 min<br>pH NM	Spherical<br>3–6 nm	Dilution<br>37°C<br>24 h<br>pH NM<br>1×10^5 CFU/ml<br>Ciprofloxacin**	*Streptococcus faecalis* ATCC 29212<br>*B. cereus* ATCC 10702<br>*E. coli* ATCC 25922<br>*Shigella flexneri* KZN	125<br>125<br>125<br>125 µg/ml	[531]
Zingiber officinale	Rhizome		Spherical<br>3–22 nm		*Streptococcus faecalis* ATCC 29212<br>*B. cereus* ATCC 10702<br>*E. coli* ATCC 25922<br>*Shigella flexneri* KZN	25<br>25<br>25<br>25 µg/ml	
Capsicum frutescens	Fruits		Spherical<br>3–18 nm		*Streptococcus faecalis* ATCC 29212<br>*B. cereus* ATCC 10702<br>*E. coli* ATCC 25922<br>*Shigella flexneri* KZN	125<br>125<br>125<br>125 µg/ml	
Brassica oleracea	Whole plant	Silver nitrate 0.9 mM/ plant extract 20 % (9 :1 v/v)<br>40 °C<br>2 h<br>pH 10	Shperical<br>4–18 nm	Diffusion<br>37°C<br>24 h<br>pH NM<br>Inoculum NM<br>Erythromycin 1.5 µg/ml	*E. coli*<br>*P. aeruginosa*<br>*Kocuria myroides*<br>*Promicromonospora*	9<br>7<br>12<br>6 mm	[532]
Borago officinalis	Leaves	Silver nitrate 1 mM/ plant extract 10 % (4:1 v/v)<br>65 °C<br>30 min	Spherical, hexagonal and irregular<br>30–80 nm	Diffusion<br>28°C<br>24 h<br>pH NM<br>1×10^6 CFU/ml	*P. aeruginosa*<br>*E. coli*<br>*S. aureus*<br>*Vibrio parahaemolyticus*	14<br>11<br>13<br>13 mm	[533]

		pH NM		No control			
Phoenix dactylifera	Seeds	Silver nitrate 1 mM/ plant extract 10 % (9:1 v/v)<br>37 °C<br>48 h<br>pH NM	Spherical<br>14–30 nm	Diffusion<br>37°C<br>24 h<br>pH NM<br>Inoculum NM<br>No control	MRSA ATCC 43300	24 mm	[534]
Zea mays	Leaves	Silver nitrate 1 mM/ plant extract 10 % (4:1v/v)<br>37°C<br>40 min<br>pH NM	Spherical<br>12.6 nm	Dilution<br>37°C<br>24 h<br>pH NM<br>1×10^8 CFU/ml<br>No control	*S. aureus* ATCC 29213<br>*E. coli* ATCC 25922	0.337<br>0.084 µg/ml	[535]
				Dilution<br>37°C<br>Incubation time NM<br>pH NM<br>1.5×10^8 CFU/ml<br>Fluconazol**	*C. albicans*	0.021 µg/ml	
Tirmania sp.	Ascocarps	Silver nitrate 1 mM/ plant extract 5 % (1:2 v/v)<br>30°C<br>72 h<br>pH NM	Spherical or irregular<br>3–15 nm	Diffusion<br>37°C<br>24 h<br>pH NM<br>Inoculum size NM<br>Gentamycin**<br>25 µg/ml	*E. coli*<br>*P. aeruginosa*<br>*Klebsiella spp*<br>*S. aureus*	26<br>28<br>25<br>22 mm	[536]
Ulmus wallichiana	Leaves	Silver nitrate 1 mM / plant extract 10 % (4:1 v/v)<br>37°C	Spherical<br>54 nm	Diffusion<br>37°C<br>24 h<br>pH NM	*S. aureus*<br>*E. coli*<br>*P. aeruginosa*<br>*K. pneumoniae*	17<br>18<br>18<br>15 mm	[537]

		24 h<br>NM		Inoculum size NM<br>Ampicillin 100 µg/ml			
Garcinia indica	Fruits	Silver nitrate 2 mM/ plant extract 10 % (1:1 v/v)<br>70°C<br>24 h<br>pH 10	Spherical and hexagonal<br>5–30 nm	Diffusion<br>37°C<br>24 h<br>pH NM<br>Inoculum NM<br>Tetracyclin 10 µg/ml	*E. coli*<br>*B. subtilis*<br>*S. aureus*<br>*P. aeruginosa*<br>*S. typhi*<br>*P. vulgaris*<br>*Serratia marcescens*	14<br>12<br>15<br>12<br>0<br>0<br>0 mm	[538]
Swertia paniculata	Arial part	Silver nitrate 1 mM / plant extract 5 % (1:1 v/v)<br>40°C<br>24 h<br>pH 7	Spherical<br>31–44 nm	Diffusion<br>37°C<br>24 h<br>pH 7<br>10^5–10^6 CFU/ml<br>No control	*S. aureus*<br>*K. pneumoniae*<br>*P. aeruginosa*	15<br>18<br>19 mm	[539]
Prunus cerasifera	Fruits	Silver nitrate 1 mM/ plant extract 10 % (5:1 v/v)<br>80°C<br>2 h<br>NM	Spherical<br>2 nm	Diffusion<br>37°C<br>24 h<br>pH NM<br>Inoculum size NM<br>Ampicillin**	*Xanthomonas axonopodus*<br>*Pseudomonas syringae*	21<br>19	[540]
				Diffusion<br>Temperature NM<br>72 h<br>pH NM<br>Inoculum size NM<br>Amphotericin B**	*A. niger*<br>*A. flavus*<br>*Aspergillus spergillus*<br>*Aspergillus fumigatus*<br>*Aspergillus terreus*<br>*Penicillium chrysogeum*<br>*Fusarium solani*<br>*Lasiodiplodia heobromae*	20<br>26<br>18<br>14<br>24<br>23<br>21<br>22 mm	

Thymbra spicata	Leaves	Silver nitrate 1 mM/<br>plant extract 10 %<br>(10:1 v/v)<br>60°C<br>12 h<br>pH NM	Spherical<br>20–50 nm	Diffusion<br>35°C<br>24 h<br>pH NM<br>1×10^5–10^6 CFU/ml<br>Gentamycin**	*B. cereus* ATCC 11778<br>*S. aureus* ATCC 25923<br>*E. coli* ATCC 25922<br>*S. typhimurium* ATCC 14028	15<br>16<br>12<br>14 mm	[541]
Sida acuta	Leaves and stems	Silver nitrate 1 mM/<br>plant extract 10 % (9:2 v/v)<br>Temperature NM<br>Incubation time NM<br>pH NM	Spherical<br>5–30 nm	Diffusion<br>Temperature NM<br>Incubation time NM<br>pH NM<br>Inoculum NM<br>Gentamycin**	*S. aureus*<br>*Streptococcus faecalis*<br>*E. coli*	9<br>0<br>11 mm	[542]
Ocimum Sanctum	Leaves	Silver nitrate 2 mM /<br>plant extract 5 % (2:1 v/v)<br>60°C<br>10 min<br>pH 10	Spherical<br>18 nm	Diffusion<br>35°C<br>48 h<br>pH NM<br>Inoculum NM<br>No control	*E. coli*	14 mm	[543]
Desmodium adscendens	Whole plant	Silver nitrate 1 mM /<br>plant extract 5 % (1:1 v/v)<br>37°C<br>5 h<br>pH NM	Spherical<br>15–100 nm	Dilution<br>37°C<br>24 h<br>pH NM<br>1×10^6 CFU/ml<br>Ciprofloxacin10 µg /ml	*E. coli* ATCC 25922<br>*S. aureus* ATCC 25923<br>*B. cereus* ATCC 11778	31.3<br>7.8<br>12.5 µg/ml	[544]
Cyperus rotundus	Weeds	Silver nitrate 15 mM/<br>plant extract 9 % (1 :1 v/v)<br>37°C<br>24 h<br>pH NM	Spherical<br>20.5 ± 9.6 nm	Diffusion<br>37°C<br>24 h<br>pH NM<br>5×10^5 CFU/ml<br>No control	*Chromobacterium haemolyticum*<br>*E. coli*<br>*B. cereus*	15<br>8<br>10 mm	[545]
Eleusin indica			Spherical<br>55.0 ± 24.1 nm		*Chromobacterium haemolyticum*<br>*E. coli*	9<br>12	

					B. cereus	10 mm	
Punica granatum	Peels	Silver nitrate 1 mM/ plant extract 10 % (1:1 v/v)<br>60°C<br>3 h<br>pH NM	Spherical<br>3–13 nm	Diffusion<br>Temperature NM<br>Incubation time NM<br>pH NM<br>Inoculum NM<br>No control	*S. aureus*<br>*P. aeruginosa*<br>*Micrococcus leutus*<br>*Bordetella bronchiseptica*	11<br>10<br>10<br>11 mm	[546]
Ardisia solanacea	Leaves	Silver nitrate 1 mM/ plant extract 10 % (9:1 v/v)<br>60°C<br>12 h<br>pH NM	Polyhedral<br>29 nm	Diffusion<br>37°C<br>24 h<br>pH NM<br>Inoculum NM<br>Gentamycin**	*B. subtilis* MTCC 736<br>*S. aureus* MTCC 737<br>*E. coli* MTCC 443<br>*P. aeruginosa* MTCC 424	16<br>0<br>0<br>28 mm	[547]
Agrimonia pilosa	Arial part	Silver nitrate 1 mM/ plant extract 10 % (10:1 v/v)<br>40°C<br>10 min<br>pH NM	Spherical and oval<br>10–20 nm	Diffusion<br>37°C<br>24 h<br>pH NM<br>Inoculum NM<br>No control	*B. cereus* ATCC 130601<br>*S. aureus* ATCC 6538<br>*E. coli* KCTC 1635<br>*Pseudomonas putida* ATCC 49128<br>*Listeria monovytogenes*ATCC 7644<br>*Staphylococcus saprophyticus* KCTC 1682	14<br>15<br>15<br>15<br>16<br>17 mm	[548]
Limonia acidissima	Leaves	Silver nitrate 5 mM/ plant extract 10 % (1:1 v/v)<br>Room temperature<br>24 h<br>pH NM	Spherical<br>21–42 nm	Diffusion<br>37°C<br>24 h<br>pH NM<br>1×10^6 CFU/ml<br>Erythromycin 15 µg	*S. aureus* MTCC 3160<br>*B. cereus* MTCC 8733<br>*Enterococcus faecalis* ATCC 35550<br>*E. coli* MTCC 433<br>*S. typhi* MTCC 3216<br>*P. aeruginosa* ATCC 25619	16<br>16<br>15<br>18<br>9<br>9 mm	[549]
Lantana camara	Leaves	Silver nitrate 1 mM/ plant extract 33 % (6:1 v/v)	Spherical<br>42.5 nm	Diffusion<br>37°C<br>24 h	*S. aureus* MTCC 87<br>*P. aeruginosa* MTCC 741<br>*E. coli* MTCC 443	28<br>21<br>22 mm	[550]

		37°C<br>24 h<br>pH NM		pH NM<br>Inoculum NM<br>Ciprofloxacin**			
Euphorbia cf. *lactea*	Latex	Silver nitrate 10 mM/ plant extract 0.2 % (19:1 v/v)<br>Room temperature<br>3 h<br>pH NM	Pseudospherical and cubic<br>57 ± 14.7 nm	Dilution<br>37°C<br>24 h<br>pH NM<br>1×10^4 CFU/ml<br>No control	*S. aureus* TISTR 1466<br>MRSA DMST 20654<br>*B. cereus* TISTR 687<br>*E. coli* TISTR 780<br>*P. aeruginosa* TISTR781	6.25<br>25<br>6.25<br>12.5<br>12.5 µg/ml	[551]
Sterculia foetida	Leaves	Silver nitrate 1 mM/ plant extract 4 % (1:9 v/v)<br>Temperature NM<br>48 h<br>pH NM	Spherical<br>50–70 nm	Diffusion<br>Temperature NM<br>Incubation time NM<br>pH NM<br>Inoculum NM<br>Gentamycin**	*S. aureus*<br>*B. cereus*<br>*E. coli*<br>*P. aeruginosa*	19<br>11<br>13<br>20 mm	[552]
Ziziphus jujuba	Whole plant	Silver nitrate 10 mM/ plant extract 3 % (1:4 v/v)<br>60°C<br>30 min<br>pH 4.85	Spherical<br>11.5 ± 4. 8 nm	Diffusion<br>37°C<br>24 h<br>pH NM<br>Inoculum NM<br>Gentamycin**	*S. aureus*<br>*E. coli*	8<br>6 mm	[553]
Rumex hymenosepalus	Roots	Silver nitrate 10 mM/ plant extract 15 % (1 :1 v/v)<br>25°C<br>24 h<br>pH NM	Spherical<br>10 nm	Diffusion<br>37°C<br>24 h<br>pH NM<br>1.5×10^8 CFU/ml<br>No control	*S. aureus* ATCC 25923<br>*Listeria monocytogenes* ATCC 7644<br>*E. coli* ATCC 11229<br>*E. coli* ATCC 43895<br>*S. typhi* ATCC 6539<br>*P. aeruginosa* ATCC 15442	20<br>18<br>11<br>17<br>17<br>16 mm	[554]
				Diffusion<br>37°C<br>24 h<br>pH NM	*C. albicans* ATCC 90028	24 mm	

				1.5×10^8 CFU/ml<br>No control			
Heterotheca inuloides	Leaves	Silver nitrate 10 mM/ plant extract 1 % (4 :1 v/v)<br>37°C<br>8 h<br>pH NM	Spherical<br>17 nm	Diffusion<br>37°C<br>24 h<br>pH NM<br>1×10^8 CFU/ml<br>No control	*S. aureus*<br>*E. coli*<br>*Lactobacillus casei*<br>*Streptococcus mutans*	3<br>2<br>5<br>7 mm	[555]
Citrus maxima	Leaves	Silver nitrate 1 mM/ plant extract 10 % (20:1 v/v)<br>Temperature NM<br>4–6 h<br>pH NM	Spherical<br>2–50 nm	Diffusion<br>37°C<br>24 h<br>pH NM<br>1×10^6 CFU/ml<br>Gentamycin 10 µg/ml	*E. coli* MG 1655<br>*B. cereus* MTCC 430<br>*B. subtilis* MTCC 121<br>*K. pneumoniae* MTCC 3384<br>*P. aeruginosa* MTCC 741<br>*S. aureus* MTCC 740	14<br>11<br>12<br>11<br>12<br>12 mm	[556]
Melissa officinalis	Leaves	Silver nitrate 1 mM/ plant extract 14 % (4:1 v/v)<br>Temperature NM<br>Incubation time NM<br>pH NM	Spherical<br>13 nm	Diffusion<br>37°C<br>24h<br>pH NM<br>1.5×10^8 CFU/ml<br>No control	*S. aureus* ATCC 25923<br>*B. cereus* B1079<br>*P. aeruginosa* ATCC 27853<br>*E. coli* ATCC 25922	12<br>20<br>12<br>15 mm	[557]
				Diffusion<br>37°C<br>24–48 h<br>pH NM<br>1.5×10^8 CFU/ml<br>No control	*C. albicans* ATCC 10231<br>*Candida parapsilosis* ATCC 22109<br>*Candida glabrata* ATCC 64677<br>*Candida krusei* ATCC 1424<br>*A. niger*<br>*Trichoderma viride*	11<br>10<br>0<br>7<br>0<br>0 mm	
Elephanto pusscaber	Leaves	Silver nitrate 1 mM / plant extract 5 % (9:1 v/v)<br>Temperature NM<br>Incubation time NM	Spherical<br>20–60 nm	Diffusion<br>37°C<br>24 h<br>pH NM<br>Inoculum NM	*B. subtilis* MTCC 441<br>*Lactococcus lactis* MTCC 3041<br>*P. aeruginosa* MTCC 424<br>*Pseudomonas fluorescens* MTCC 2421	10<br>6<br>6<br>5 mm	[558]

		pH NM		Streptomycin**			
Artemisia quttensis	Aerial part	Silver nitrate 1 mM/ plant extract 10 % (1:15 v/v)<br>Temperature NM<br>Incubation time NM<br>pH NM	Spherical<br>10.9 ± 8.1 nm	Diffusion<br>37°C<br>24 h<br>pH NM<br>1×10^8 CFU/ml<br>Ampicillin**	*S. aureus* ATCC 6538<br>*P. aeruginosa* ATCC 15442<br>*E. coli* ATCC 6633	14<br>7<br>10 mm	[559]
Cucurbita maxima	Fruit Pulp, Seeds, Flowers and Callus	Silver nitrate 1 mM/ plant extract 5 % (9:1 v/v)<br>60°C<br>Incubation time NM<br>pH NM	Spherical<br>7.3–15.6 nm	Diffusion<br>37°C<br>24 h<br>pH NM<br>Inoculum NM<br>Ciprofloxacin 10 µg	*E. coli*	14 mm	[560]
Corchorus capsularis	Leaves	Silver nitrate 1 mM/ plant extract 2 % (1 :1 v/v)<br>NM<br>10 min<br>pH NM	Spherical and ellipsoidal<br>20.5 nm	Diffusion<br>37°C<br>24 h<br>pH NM<br>1×10^6 CFU/ml<br>Gentamycin 15 µg	*P. aeruginosa*<br>*S. aureus*	21<br>15 mm	[561]
Mentha pulegium	NM	Silver nitrate 10 mM/ plant extract 4 % (1:4 v/v)<br>Room temperature<br>Incubation time NM<br>pH NM	Anisotropic<br>5–50 nm	Diffusion<br>37°C<br>24 h<br>pH NM<br>1×10^5 CFU/ml<br>Ampicillin 10 µg	*E. coli* ATCC 25922<br>*S. aureus* ATCC 25923<br>*S. pyogenes* ATCC 1447	10<br>11<br>10 mm	[562]

Diospyros sylvatica	Roots	Silver nitrate 1 mM/ plant extract** (5 :1 v/v)<br>60°C<br>3 h<br>pH NM	Spherical<br>10–40 nm	Diffusion<br>37°C<br>18 h<br>pH NM<br>Inoculum size NM<br>Chloramphenicol 100 µg/ml	*B. subtilis* MTCC 441<br>*Bacilllus pumilis* MTCC 432<br>*S. pyogenes* MTCC 2327<br>*P. aeuriginosa* MTCC 424<br>*S. aureus* MTCC 3160<br>*E. coli* MTCC 443<br>*K. pneumoniae* MTCC 452<br>*P. vulgaris* MTCC 426	19<br>23<br>19<br>16<br>16<br>13<br>15<br>20 mm	[300]
				Diffusion<br>37°C<br>48 h<br>pH NM<br>Inoculum size NM<br>Nystatin 100 µg/ml	*A. niger* MTCC 961<br>*A. flavus* MTCC 149<br>*Penicillium notatum* MTCC 722<br>*Saccharomyces cerevisiae* MTCC 783	15<br>12<br>13<br>15 mm	
Trigonella foenum graecum	Leaves	Silver nitrate 1 mM / plant extract 2.5 % (49:1 v/v)<br>37°C<br>24 h<br>pH NM	Spherical<br>2–15 nm	Diffusion<br>37°C<br>24 h<br>pH 7.3<br>Inoculum NM<br>Ciproflaxacin**	*Enterococcus faecalis*<br>*S. aureus*<br>*Acinetobacter baumannii*<br>*Enterobacter asburiae*<br>*Enterobacter cloacae*<br>*Enterobacter kobei*<br>*E. coli*<br>*K. pneumoniae*<br>*Proteus mirabilis*<br>*P. aeruginosa*	9<br>10<br>8<br>10<br>10<br>9<br>10<br>8<br>9<br>7 mm	[563]
Catharanthus roseus	Callus	Silver nitrate 1 mM/ plant extract 10 % (1:1 v/v)<br>25°C<br>96 h<br>pH NM	Spherical<br>2–15 nm	Diffusion<br>37°C<br>24 h<br>pH NM<br>Inoculum NM<br>No control	*E. coli* ATCC 25922	5 mm	[564]

Artemisia vulgaris	Leaves	Silver nitrate 20 mM/ plant extract 1 % (1:1 v/v)<br>Room temperature<br>2 h<br>pH NM	Spherical<br>27–53 nm	Diffusion<br>37°C<br>24 h<br>pH NM<br>10^5–10^6 CFU/ml<br>Gentamycin**	*E. coli*<br>*S. aureus*<br>*P. aeruginosa*<br>*K. pneumoniae*<br>*Haemophilus influenza*	14<br>18<br>14<br>13<br>16 mm	[565]
Momordica charantia	Fruits	Silver nitrate 1 mM/ plant extract 20 % (1:3 v/v)<br>NM<br>2 h<br>pH NM	Circular<br>78.5–100 nm	Diffusion<br>37°C<br>24 h<br>pH NM<br>Inoculum NM<br>Ciprofloxacin**	*S. aureus* ATCC 25923<br>*S. typhi* ATCC 14028<br>*E. coli* ATCC 25922<br>*P. aeruginosa* ATCC 27853	24<br>7<br>345<br>26 mm	[566]
Trigonella foenum graecum	Leaves	Silver nitrate 1 mM/ plant extract 5 % (20:1 v/v)<br>35°C<br>96 h<br>pH NM	Spherical<br>20–30 nm	Diffusion<br>35°C<br>24 h<br>pH NM<br>Inoculum NM<br>$1×10^5$ CFU/ml<br>Streptomycin**	*S. aureus* ATCC 29736<br>*E. coli* ATCC 8739	12<br>16 mm	[567]
Syzygium cumini	Leaves	Silver nitrate 5 mM/ plant extract 10 % (10:1 v/v)<br>Room temperature<br>6 h<br>pH NM	Spherical<br>16 ± 2 nm	Diffusion<br>37°C<br>24 h<br>pH NM<br>$1×10^8$ CFU/ml<br>No control	*S. aureus*<br>*P. aeruginosa*	14<br>18 mm	[568]
Arbutus unedo	Leaves	Silver nitrate 3 mM / plant extract 10 % (10:1 v/v)<br>80°C<br>0.5 h<br>pH 4.3	Spherical<br>40–50 nm	Dilution<br>37°C<br>48 h<br>pH NM<br>$5×10^6$ CFU/ml<br>No control	*S. epidermidis* C5M6<br>*B. subtilis subsp.* Bg G11<br>*E. coli* DH5a<br>*P. aeruginosa* ADD1976	15<br>3<br>15<br>15 µg/ml	[569]
Carica papaya	Leaves		Spherical	Diffusion	*E. coli*	18	[570]

		Silver nitrate 150 mM/<br>plant extract 3 % (1:1 v/v)<br>37°C<br>24 h<br>pH NM	13–69 nm	37°C<br>24 h<br>pH NM<br>5×10^5 CFU/ml<br>No control	*B. cereus*	17 mm	
Manihot esculenta			Spherical<br>13–38 nm		*E. coli*<br>*B. cereus*	17<br>17 mm	
Morinda citrifolia			Spherical<br>9–54 nm		*E. coli*<br>*B. cereus*	16<br>13 mm	
Curcuma aromatica	Tuber	Silver nitrate 1 mM/<br>plant extract 20 %<br>(10:1 v/v)<br>37°C<br>24 h<br>pH NM	Triangular and spherical<br>10–40 nm	Dilution<br>37°C<br>24h<br>pH NM<br>Inoculum NM<br>No control	*S. aureus*	1.95 µg/ml	[571]
Decalepis hamiltonii	Roots	Silver nitrate 1 mM/<br>plant extract 10 %<br>(9:1 v/v)<br>37°C<br>24 h<br>pH NM	Spherical<br>32.5 nm	Diffusion<br>37°C<br>24 h<br>pH NM<br>Inoculum NM<br>Norfloxacin**	*B. cereus* MTCC 430<br>*Bacillus licheniformus* MTCC 2465<br>*E. coli* MTCC 10312<br>*S. aureus* MTCC 7443<br>*P. aeruginosa* ATCC 27853	15<br>13<br>14<br>12<br>17 mm	[572]
Cardiospermum halicacabum	Leaves	Silver nitrate 1 mM/<br>plant extract 10 % (9:1 v/v)<br>Room temperature<br>16 h<br>pH NM	Spherical<br>74 nm	Diffusion<br>37°C<br>24 h<br>pH NM<br>Inoculum NM<br>Streptomycin**<br>Ampicillin**	*P. vulgaris* MTCC 426<br>*P. aeruginosa* MTCC 2453<br>*S. aureus* MTCC 96<br>*B. subtilis* MTCC 441<br>*S. paratyphi* MTCC 735	15<br>15<br>15<br>16<br>21 mm	[573]
				Diffusion<br>37°C<br>24 h<br>pH NM<br>Inoculum NM<br>Ketoconazol**	*Alternaria solania*<br>*Fusarium porum*	8<br>18 mm	

Malus domestica	Leaves	Silver nitrate 1 mM/ plant extract 50 % (9:1 v/v)<br>80°C<br>16 h<br>pH NM	Spherical<br>30.3 ± 5.3 nm	Dilution<br>37°C<br>24 h<br>pH NM<br>5×10^5CFU/ml<br>No control	*E. coli*<br>*S. aureus*<br>MRSA<br>*P. aeruginosa*	125<br>1000<br>1000<br>500 µg/ml	[574]
Ziziphus nummularia	Leaves	Silver nitrate 1 mM/ plant extract 5 % (12:1 v/v)<br>Room temperature<br>4 h<br>pH NM	Spherical<br>30–85 nm	Diffusion<br>37°C<br>24 h<br>pH NM<br>Inoculum NM<br>No control	*E. coli*<br>*P. aeruginosa*<br>*S. aureus*<br>*S. typhi*<br>*B. cereus*	18<br>17<br>18<br>21<br>16 mm	[575]
				Dilution<br>Room temperature<br>96 h<br>8 days<br>Inoculum NM<br>No control	*A. niger*<br>*A. flavus*<br>*C. albicans*	120<br>150<br>95 µg/ml	
Pedalium murex	Leaves	Silver nitrate 1 mM/ plant extract 5 % (2:1 v/v)<br>Temperature NM<br>Incubation time NM<br>pH NM	Spherical<br>20–50 nm	Diffusion<br>37°C<br>24 h<br>pH NM<br>1×10^8 CFU/ml<br>Ofloxacin 2 mg/ml	*B. substilis* ATCC 6633<br>*S. aureus* ATCC 29737<br>*E. coli* ATCC 8739<br>*Micrococcus flavus* ATCC 25619<br>*P. aeruginosa* ATCC 25619<br>*K. pneumoniae* ATCC 10031<br>*B. pumilis* ATCC 12228	10<br>10<br>11<br>9<br>11<br>9<br>10 mm	[576]
Dracocephalum moldavica	Seeds	Silver nitrate 1 mM/ plant extract 20 % (100:1 v/v)<br>60°C<br>24 h<br>pH NM	Spherical<br>5–50 nm	Diffusion<br>37°C<br>24 h<br>pH NM<br>Inoculum NM<br>No control	*E. coli*<br>*P. vulgaris*<br>*S. aureus*<br>*Serratia marcescens*<br>*S. epidermidis*<br>*B. subtilis*	18<br>13<br>8<br>20<br>11<br>10 mm	[577]

Biophytum sensitivum	Fruits	Silver nitrate 1 mM/ plant extract 10 % (10:1 v/v)<br>Room temperature<br>1 h<br>pH NM	Spherical<br>7.4 nm	Diffusion<br>37°C<br>24 h<br>pH NM<br>1×10^8 CFU/ml<br>Ciprofloxacin 30 μg	*E.coli* ATCC 1222<br>*S. aureus* ATCC 6538-P	13<br>11 mm	[578]
Helicteres isora	Roots	Silver nitrate 1 mM/ plant extract 1 % (1:1 v/v)<br>Room temperature<br>6 h<br>pH NM	Spherical<br>16–95 nm	Diffusion<br>37°C<br>24 h<br>pH NM<br>Inoculum NM<br>Streptomycin**	*E. coli*<br>*Vibrio cholera*<br>*S. typhi*<br>*P. aeruginosa*<br>*B. subtilis*<br>*Micrococcus luteus*	4<br>6<br>8<br>7<br>8<br>8 mm	[579]
Psidium guajava	Leaves	Silver nitrate 1 mM/ plant extract 8 % (100:1 v/v)<br>30°C<br>10 min<br>pH NM	Spherical<br>10–90 nm	Diffusion<br>37°C<br>24 h<br>pH NM<br>Inoculum NM<br>No control	*P. aeruginosa* MTCC 741	8 mm	[580]
Vigna radiate	Seeds	Silver nitrate 1 mM/ plant extract 20 % (9:1 v/v)<br>Temperature NM<br>Incubation time NM<br>pH NM	Spherical<br>18 nm	Diffusion<br>37°C<br>24 h<br>pH NM<br>Inoculum NM<br>No control	*E. coli*<br>*S. aureus*	20<br>16 mm	[581]
Anthemis tropatana	Arial parts	Silver nitrate 1 mM / plant extract 10 % (25:1 v/v)<br>Room temperature<br>10 min<br>pH NM	Spherical<br>38.9 nm	Dilution<br>37°C<br>24 h<br>pH NM<br>5×10^5 CFU/ml<br>No control	*S.aureus* ATCC 6538<br>*S. pyogenes* ATCC 19615<br>*P. aeruginosa* ATCC 15442<br>*E. coli* ATCC 25922	12.5<br>50<br>25<br>100 µg/ml	[582]

Descurainia sophia	Seeds	Silver nitrate 4 mM /plant extract 10 % (9:1 v/v)<br>28°C<br>10 min<br>pH 4.5	Spherical<br>1–35 nm	Dilution<br>37°C<br>48 h<br>pH NM<br>1.5×10^8 CFU/ml<br>Rifampicin**	*Agrobacterium tumefaciens* 15843<br>*Agrobacterium rhizogenes* GV 3850	4<br><br>4 µg/ml	[583]
				Diffusion<br>28°C<br>3 days<br>pH NM<br>Inoculum NM<br>No control	*Rhizoctonia solani*	15 mm	
Cocoa pod husk	Host	Silver nitrate 1 mM / plant extract 10 % (40:1 v/v)<br>30°C<br>10 min<br>pH NM	Spherical<br>4–32 nm	Diffusion<br>37°C<br>18 h<br>pH NM<br>1×10^6 CFU/ml<br>Cefuroxime 30 µg<br>Ampicillin 10 µg	*E. coli*<br>*K. pneumoniae*	14<br>10 mm	[584]
				Diffusion<br>30 ± 2°C<br>48 h<br>pH NM<br>1×10^6 CFU/ml<br>No control	*A. flavus*<br>*Aspergillus fumigatus*<br>*A. niger*	25<br>23<br>24 mm	
Aloe vera	Leaves	Silver nitrate 10 mM /plant extract 10% (1:1 v/v)<br>Temperature NM<br>Incubation time NM<br>pH NM	Octahedron<br>5–50 nm	Diffusion<br>37°C<br>24 h<br>pH NM<br>1×10^7 CFU/ml<br>Gentamycin**	*S. aureus*<br>*B. cereus*<br>*Micrococcus luteus*<br>*E. coli*<br>*K. pneumonia*	43<br>38<br>33<br>42<br>38 mm	[585]

Salmalia malabarica	Gum	Silber nitrate 1 mM/<br>plant extract 5 % (1:1 v/v)<br>120°C<br>Incubation time NM<br>pH NM	Spherical<br>7 ± 2 nm	Diffusion<br>37°C<br>24 h<br>pH NM<br>Inoculum NM<br>Ampicillin**	*E. coli*<br>*S. aureus*	16<br>18 mm	[586]
Taxus yunnanensis	Callus	Silver nitrate 1 mM/<br>plant extract 20 %<br>(9 :1 v/v)<br>37°C<br>24 h<br>pH 9	Spherical<br>6.4–27.2 nm.	Dilution<br>37°C<br>24 h<br>pH NM<br>1×10^6 CFU/ml<br>Gentamycin 1 mM	*S. aureus* CMCC(B) 26003<br>*S. paratyphi B* CMCC(B) 50094<br>*B. subtilis* CMCC(B) 63501<br>*E. coli* CMCC(B) 44102	2<br>2<br>2<br>2 µg/ml	[587]
Thevetia peruviana	Leaves	Silver nitrate 1 mM/<br>plant extract 10 % (4:1 v/v)<br>50°C<br>30 min<br>pH NM	Spherical<br>18.1 nm	Diffusion<br>37°C<br>18–24 h<br>pH 7<br>Inoculum NM<br>No control	*E. coli*<br>*P. aeruginosa*<br>*K. pneumoniae*<br>*S. aureus*<br>*B. subtilis*<br>*S. typhi*	10<br>10<br>15<br>20<br>11<br>12 mm	[588]
				Diffusion<br>28°C<br>18–24 h<br>pH 7<br>1×10^5 CFU/ml<br>No control	*C. albicans*<br>*A. niger*	20<br>20 mm	
Hesperidin *(Citrus sinensis)*	Leaves	Silver nitrate 1 mM/<br>plant extract 0.02 %<br>(9:1 v/v)<br>Temperature NM<br>Incubation time NM<br>pH 7	Oval<br>5–50 nm	Diffusion<br>37°C<br>24 h<br>pH NM<br>2.5×10^5 CFU/ml<br>No control	*E. coli* MTCC 4604<br>*Pseudomonas putida* MTCC 1194<br>*S. aureus* NCIM 2127	6<br>5<br>7 mm	[589]
Naringin (*Citrus sinensis*)			Oval<br>5–40 nm		*E. coli* MTCC 4604<br>*Pseudomonas putida* MTCC 1194<br>*S. aureus* NCIM 2127	6<br>5<br>5 mm	

Diosmin (*Citrus sinensis*)			Hexagonal<br>20–80 nm		*E. coli* MTCC 4604<br>*Pseudomonas putida* MTCC 1194<br>*S. aureus* NCIM 2127	6<br>6<br><br>7 mm	
Indigofera tinctoria	Leaves	Silver nitrate 1 mM/ plant extract 10 % (9:1 v/v)<br>Temperature NM<br>Incubation time NM<br>pH NM	Spherical<br>9–26 nm	Diffusion<br>37°C<br>24–48h<br>pH NM<br>Inoculum NM<br>No control	*Pseudomonas spp*<br>*B. pumilis*<br>*S. aureus*<br>*E. coli*	28<br>13<br>22<br>25 mm	[590]
				Diffusion<br>37°C<br>24–48 h<br>pH NM<br>Inoculum size NM<br>No control	*Aspergillus fumigates*<br>*A. niger*	16<br>18 mm	
Justicia adhatoda	Roots	Silver nitrate 1mM/ plant extract 10 % (10:1 v/v)<br>60°C<br>20 min<br>pH NM	Spherical<br>25 nm	Diffusion<br>37°C<br>24 h<br>pH NM<br>Inoculum NM<br>Tétracyclin**	*S. paratyphi*<br>*P. aeruginosa*	15<br>10 mm	[591]
Skimmia laureola	Leaves	Silver nitrate 1 mM/ plant extract 10 % (10:1 v/v)<br>Room temperature<br>Incubation time NM<br>pH NM	Spherical and hexagonal<br>38±0.3 nm	Diffusion<br>37°C<br>24 h<br>pH NM<br>Inoculum NM<br>Tetracyclin 10 µg/ml	*E. coli*<br>*K. pneumoniae*<br>*P. aeruginosa*<br>*P. vulgaris*<br>*S. aureus*	14<br>12<br>13<br>13<br>13 mm	[592]
Justicia adhatoda	Leaves	Silver nitrate 1 mM/ plant extract 8 % (7:3 v/v)<br>60°C	Spherical<br>5–50 nm	Diffusion<br>37°C<br>24 h<br>pH NM	*P. aeruginosa* MTCC 741	9 mm	[593]

		0.5 h<br>pH NM		Inoculum NM<br>No control			
Caesalpinia ferrea	Seed	Silver nitrate 3 mM/ plant extract 0.1 % (99:1 v/v)<br>25°C<br>96 h<br>pH NM	Spheroidal<br>30–50 nm	Dilution<br>35 ± 2°C<br>48 h<br>pH 7<br>5–25×10^2 CFU/ml<br>Amphotericin B**<br>Nystatin**	*C. albicans* ATCC 10231<br>*Candida glabrata*<br>*Candida krusei*<br>*Candida guilliermondii*	312.5<br>156,3<br>312.5<br>156.3 µg/ml	[594]
Alphonsea sclerocarpa	Leaves	Silver nitrate 1 mM/ plant extract 3 % (2.5:1 v/v)<br>65°<br>1 h<br>pH NM	Irregular<br>20–40 nm	Diffusion<br>25 °C<br>72 h<br>pH NM<br>1.5×10^8 CFU/ml<br>Fluconazol 10µg/100µl	*Aspergillus brasiliensis*<br>*A. flavus*<br>*A. niger*<br>*Alternaria solani*<br>*C. albicans*	20<br>10<br>11<br>10<br>12mm	[595]
Oryza sativa	Seeds	Silver nitrate 1 mM/ plant extract 20 % (4:1 v/v)<br>Room temperature<br>24 h<br>pH NM	Spherical<br>17.3 ± 0.9 nm	Diffusion<br>25°C<br>2–3 days<br>pH NM<br>Inoculum NM<br>No control	*Purycularia oryzae*	28 mm	[596]
Saccharum officinarum	Leaves	Silver nitrate 1 mM/ plant extract 20 % (8:1 v/v)<br>Temperature NM<br>Incubation time NM<br>pH NM	Spherical<br>20–50 nm	Diffusion<br>Temperature NM<br>Incubation time NM<br>pH NM<br>Inoculum NM<br>Nystatin**	*Phytophthora capsici*<br>*Phytophthora drechsleri*<br>*Didymella bryoniae*<br>*Colletotrichum acutatum*<br>*Cladosporium fulvum*	5<br>4<br>0<br>4<br>4 mm	[597]
Gymnema sylvestre	Callus	Silver nitrate 1 mM/ plant extract 10 % (9:1 v/v)<br>Temperature NM	Spherical<br>3–30 nm	Diffusion<br>28°C<br>24 h<br>pH NM	*C. albicans*<br>*Candida nonalbicans*<br>*Candida tropicalis*	15<br>13<br>16 mm	[598]

		24 h<br>pH NM		Inoculum size NM<br>Voriconazole**<br>Amphotericin B**			
Arnicae anthodium	Leaves	Silver nitrate 1 mM/ plant extract 4.5 % (9:1 v/v)<br>25°C<br>24 h<br>pH NM	Spherical<br>90–118 nm	Dilution<br>34 °C<br>18 h<br>pH NM<br>1×10^6 CFU/ml<br>Nystatin**	*C. albicans* ATCC 10231	0.2 µg/ml	[599]
Amaranthus gangeticus	Leaves	Silver nitrate 1 mM/ plant extract 10 % (5:1.5 v/v)<br>20 min<br>80°C<br>pH NM	Globular<br>11–15 nm	Dilution<br>25°C<br>48 h<br>pH NM<br>1×10^8 CFU/ml<br>No control	*Sclerotinia spp*	0.2 µg/ml	[307]
Syzygium alternifolium	Stem bark	Silver nitrate 1 mM/ plant extract 25 % (10:1 v/v)<br>60–80°C<br>1 h<br>pH NM	Spherical<br>4–48 nm	Diffusion<br>37°C<br>24 h<br>pH NM<br>Inoculum NM<br>Fluconazole 50 µg/ml	*Alternaria solani*<br>*A. niger*<br>*A. flavus*<br>*Penicillium chrysogenum*<br>*Trichoderma harzianum*	10<br>9<br>11<br>10<br>11 mm	[600]
Alpinia galanga	Rhizome	Silver nitrate 1 mM/ plant extract 5 % (9:1 v/v)<br>Temperature NM<br>3 h<br>pH NM	Spherical<br>20.8 ± 1.8 nm	Diffusion<br>37°C<br>48 h<br>pH NM<br>Inoculum size NM<br>No control	*A. niger*	29 mm	[601]
Guignardia mangiferae	Leaves	Silver nitrate 1 mM/ plant extract 5% (1:1 v/v)<br>48 h<br>80°C	Spherical<br>5–30 nm	Diffusion<br>37°C<br>20 h<br>pH 7<br>5×10^5 CFU/ ml	*A. niger*<br>*Colletotrichum spp*<br>*Curvularia lunata*<br>*Fusarium spp*<br>*Rhizoctonia solani*	9<br>12<br>11<br>10<br>12 mm	[602]

		pH 10		Amphotericin B 1 mg/ ml			
Phoenix dactylifera	Fruits	Silver nitrate 5 mM/ plant extract 20 % (2:1 v/v)<br>28°C<br>Temperature NM<br>pH NM	Spherical<br>1–40 nm	Diffusion<br>28 ± 1°C<br>72 h<br>pH NM<br>Inoculum NM<br>No control	*Rhizoctonia solani*	18 mm	[603]
Ocimum tenuiflorum	Leaves	Silver nitrate 2 mM/ plant extract 10 % (9:1 v/v)<br>Room temperature<br>24 h<br>pH NM	Spherical<br>2–7 nm	Dilution<br>35°C<br>24 h<br>pH NM<br>4.5×10^5 CFU/ml<br>Fluconazole 4 µg	*C. albicans* ATCC 90028<br>*Candida tropicalis*ATCC 750<br>*Candida glabrata* ATCC 90030	60<br>30<br>30 µg/ml	[604]
Erythrina suberosa	Leaves	Silver nitrate 1 mM/ plant extract 10 % (9:1 v/v)<br>Room temperature<br>24 h<br>pH NM	Spherical<br>15–34 nm	Diffusion<br>Temperature NM<br>Incubation time NM<br>pH NM<br>Inoculum size NM<br>Clotrimazole**	*C. albicans* MTCC227<br>*Candida kruseii* MTCC 9215<br>*Trichophyton mentagrophytes* MTCC 8476<br>*Candida viswanathii* MTCC 1929	0<br>15<br>16<br>0 mm	[605]
Brassica rapa	Leaves	Silver nitrate 1 mM/ plant extract 30 % (5:1 v/v)<br>28°C<br>24 h<br>pH NM	Spherical<br>16.1 nm	Diffusion<br>30 °C<br>48 h<br>pH NM<br>Inoculum NM<br>No control	*Gloeophyllum abietinum* KACC 51949<br>*Gloeophyllum trabeum* KACC 43361<br>*Chaetomium globosum* KACC 42262<br>*Phanerochaete sordida* KACC 43367	3<br>8<br>6<br>12 mm	[606]
Phyllanthus urinaria	Leaves	Silver nitrate 1 mM/ plant extract 2 % (1:10 v/v)<br>25°C<br>8 h	Spherical<br>28.3 nm	Diffusion<br>Room temperature<br>96 h<br>pH NM<br>Inoculum size NM	*A. niger*<br>*Fusarium oxysporum*<br>*A. flavus*	10<br>12<br>16 mm	[607]
Pouzolzia zeylanica			Hexagonal and triangular		*A. niger*	6	

		pH 7	26.7 nm	No control	*Fusarium oxysporum*<br>*A. flavus*	8<br>11 mm	
Scoparia dulcis			Spherical<br>5 nm		*A. niger*<br>*Fusarium oxysporum*<br>*A. flavus*	13<br>12<br>15 mm	
Tagetes erecta	Flower	Silver nitrate 1 mM/ plant extract 10 % (20:3 v/v)<br>Room temperature<br>24 h<br>pH NM	Spherical, triangular and irregular<br>10–90 nm	Diffusion<br>Temperature NM<br>Incubation time NM<br>pH NM<br>Inoculum size NM<br>Nystatin 30 µg/ml	*C. albicans* NCIM 3102<br>*Candida glabrata* NCIM 3448<br>*Candida neoformans* NCIM 3542	32<br>21<br>25 mm	[608]
Allium cepa	Bulb	Silver nitrate 5 mM/ plant extract 30 % (5:1 v/v)<br>50°C<br>1 h<br>pH 12	Spherical<br>1–10 nm	Dilution<br>28°C<br>48 h<br>pH NM<br>1×10^{8} CFU/ml<br>No control	*Fusarium oxysporum* MTCC 3656	10.7 µg/ml	[609]
Musa acuminate	Leaves		Spherical<br>15–25 nm		*Fusarium oxysporum* MTCC 3656	5.4 µg/ml	
Alstonia scholaris	Bark	Silver nitrate 1 mM/ plant extract 10 % (9:1 v/v)<br>Temperature NM<br>Incubation time NM<br>pH NM	Spherical<br>50 nm	Diffusion<br>37°C<br>10 days<br>pH NM<br>Inoculum size NM<br>No control	*Aspergillus fumigates*<br>*A. niger*<br>*Aspergillus clavatus*<br>*Cephalosporium acremonium*<br>*Trichoderma spp*<br>*R. sporus*	4<br>4<br>3<br>2<br>3<br>3 mm	[610]
Allamanda cathartica	Leaves	Silver nitrate 1 mM/ plant extract 5 % (1:1 v/v)<br>Temperature NM<br>Incubation time NM<br>pH NM	Spherical<br>19–34.9 nm	Diffusion<br>Temperature NM<br>7 days<br>pH NM<br>Inoculum NM<br>Nystatin**	*A. flavus*<br>*A. niger*<br>*Curcuvalaria lunata*<br>*Fusarium porum*<br>*Rhizopus arrhizus*	12<br>10<br>12<br>7<br>13 mm	[611]

Rhinacanthus nasutus	Leaves	Silver nitrate 1 mM/ plant extract 10 % (9:1 v/v)<br>60–80°C<br>20 min<br>pH NM	Spherical<br>22 nm	Diffusion<br>37°C<br>48 h<br>pH NM<br>Inoculum size NM<br>Ciprofloxacin 10 µg	*A. niger*<br>*A. flavus*	18<br>19 mm	[612]
Sesuvium portulacastrum	Callus	Silver nitrate1 mM/ plant extract 20 % (9:1 v/v)<br>Temperature NM<br>24 h<br>pH NM	Spherical<br>5–20 nm	Diffusion<br>37°C<br>24 h<br>pH NM<br>10^3 CFU/ml<br>No control	*Alternaria alternate*<br>*Penicillium italicum*<br>*Fusarium equisetii*<br>*C. albicans*	16<br>18<br>17<br>13 mm	[613]
	Leaf				*Alternaria alternate*<br>*Penicillium italicum*<br>*Fusarium equisetii*<br>*C. albicans*	15<br>14<br>14<br>15 mm	
Brassica oleracea	Leaves	Silver nitrate 1 mM/ plant extract 8 % (10:1 v/v)<br>Room temperature<br>10 min<br>pH NM	Spherical<br>20 nm	Diffusion<br>37°C<br>24 h<br>pH NM<br>1×10^8 CFU/ml<br>No control	*Bacteroides fragilis* ATCC 25285<br>*P. aeruginosa* ATCC 9027<br>*S. aureus* ATCC 6538<br>*Enterococcus faecalis* ATCC 33186<br>*S. pneumoniae* ATCC 10015<br>*P. mirabilis* ATCC 12453<br>*K. pneumoniae* ATCC 10031<br>*E. coli* ATCC25922<br>*S. epidermidis* ATCC 12228	9<br>12<br>10<br>11<br>10<br>11<br>10<br>10<br>14 mm	[614]
Citrus tangerina	Peels	Silver nitrate 1 mM/ plant extract 10 % (40:3 v/v)<br>Room temperature<br>5 h<br>pH NM	Spherical<br>10–70 nm	Diffusion<br>37°C<br>48–72 h<br>pH NM<br>Inoculum NM<br>No control	*E. coli*<br>*S. aureus*	20<br>14 mm	[615]
Citrus sinensis			Spherical<br>5–80 nm		*E. coli*<br>*S. aureus*	21<br>17 mm	
Citrus limon			Spherical<br>10–50 nm		*E. coli*<br>*S. aureus*	20<br>16 mm	

Abelmoschus esculentus	Flowers	Silver nitrate 1 mM/<br>plant extract 5 %<br>(25:1 v/v)<br>Room temperature<br>72 h<br>pH NM	Spherical<br>16.2 nm	Diffusion<br>37°C<br>24 h<br>pH NM<br>1×10^6 CFU/ml<br>Ciprofloxacin 1 mg/ml	*B. subtilis* MTCC 441<br>*S. aureus* ATCC 29213<br>*S. epidermidis* MTCC 3615<br>*S. pyogenes* ATCC 29213<br>*K. pneumoniae* G455<br>*E. coli* ATCC *25922*<br>*P. aeruginosa* ATCC 27584<br>*P. vulgaris* ATCC 8427<br>*S. typhi* ATCC 14028<br>*Shigella sonnei*	12<br>13<br>12<br>13<br>14<br>13<br>11<br>16<br>12<br>14 mm	[616]
Amorphophallus paeoniifolius	Tuber	Silver nitrate 10 mM/<br>plant extract 0.1 %<br>(1:1 v/v)<br>Temperature NM<br>2–3 min<br>pH NM	Spherical<br>22.5 nm	Diffusion<br>37°C<br>48 h<br>pH NM<br>Inoculum NM<br>Ciprofloxacin**	*E. coli*<br>*Citrobacter freundii*<br>*B. subtilis*<br>*P. aeruginosa*<br>*S. typhi*<br>*S. aureus*	18<br>16<br>14<br>20<br>17<br>13 mm	[617]
Rumexa cetosa	Leaves	Silver nitrate 6 mM/<br>plant extract 20 %<br>(10 :1 v/v)<br>60°C<br>12 h<br>pH 7.5	Spherical<br>5–80 nm	Diffusion<br>37°C<br>24 h<br>pH 7.2<br>Inoculum NM<br>No control	*B. cereus*<br>*Enteroccocus spp*<br>*E. coli*<br>*Klebsiella spp*<br>*Proteus mirabilis*<br>*Proteus vulgaris*<br>*Pseudomonas spp*<br>*S. paratyphy A*<br>*S. typhi*<br>*S. typhimurium*<br>*Serratia*<br>*Shigella dysentery A*<br>*Shigella flexneri*<br>*S. aureus*<br>*S. epidermidis*<br>*Vibrio cholera*	16<br>15<br>18<br>15<br>15<br>16<br>13<br>13<br>14<br>15<br>16<br>18<br>17<br>18<br>18<br>16 mm	[618]

Vaccinium arctostaphlyos	Leaves	Silver nitrate 3 mM/ plant extract 10 % (1:1 v/v)<br>Room temperature<br>20 min<br>pH NM	Spherical<br>7–16 mm	Diffusion<br>37°C<br>24 h<br>pH NM<br>1×10^8 CFU/ml<br>Gentamycin**<br>Streptomycin**	*S. aureus* ATCC 25923<br>*B. subtilis* ATCC 9372<br>*E. coli* ATCC 25922	14<br>15<br>11 mm	[619]
Stenocereus queretaroensis	Fruit peels	Silver nitrate 2 mM/ plant extract 1 % (20:1 v/v)<br>90°C<br>24 h<br>pH NM	Spherical<br>99 nm	Dilution<br>37°C<br>24 h<br>pH NM<br>1.5×10^8 CFU/ml<br>No control	*E. coli*<br>*S. enterica*<br>*P. aeruginosa*<br>*S. aureus*<br>MRSA	0.3<br>0.1<br>0.2<br>0.3<br>0.3 µg/ml	[620]
Ocimum tenuiflorum	Leaves	Silver nitrate 1 mM/ plant extract 1.5 % (1:10 v/v)<br>37°C<br>24–48 h<br>pH NM	Irregular<br>28 nm	Diffusion<br>35°C<br>18 h<br>pH NM<br>Inoculum NM<br>No control	*S. aureus*<br>*P. aeruginosa*<br>*E. coli*<br>*K. pneumoniae*	25<br>20<br>30<br>19 mm	[621]
Lippia citriodora	Leaves	Silver nitrate 1 mM/ plant extract 10 % (1:1 v/v)<br>37°C<br>24 h<br>pH NM	Spherical<br>20 nm	Diffusion<br>37 °C<br>24 h<br>pH NM<br>Inoculum NM<br>Tetracyclin 0.1 mg/ml	*S. aureus*	19 mm	[622]
Ocimum gratissimum	Leaves	Silver nitrate 5 mM/ plant extract 2.5 % (1:0.06 v/v)<br>Room temperature<br>24 h<br>pH 9	Hexagonal<br>12-60 nm	Disc diffusion<br>37°C<br>24 h<br>pH NM<br>10^5–10^6 CFU/ml<br>No control	*Staphylococcus aureus* MTCC7443<br>*Micrococcus luteus* MTCC 4821<br>*B. Subtilis* MTCC 2389<br>*E. coli* MTCC2127<br>*K. pneumoniae* MTCC 7162	7.5<br><br>8<br>8<br>10<br>12.5 mm	[623]

*MIC=minimal inhibition concentration; ZOI=zone of inhibition; PI=percentage of inhibition

**The quantity or concentration is not mentioned.

NM=not mentioned, MRSA=methicillin-resistant *S. aureus*

Table S2. Green gold nanoparticles exhibiting antibacterial and antifungal activities

Plant type	Part used	Operative conditions for synthesis	NP characteristics (shape and size)	Microbiological analyses (operative conditions)			Ref.
				**Methods, Incubation temperature, incubation time, pH, inoculum density, positive control**	**Tested bacteria**	**MIC, DOI or PI***	
Convolvulus fruticosus	Flowers	Chloroauric acid 5 mM / plant extract 15 % (5:2 v/v)<br>50°C<br>24 h<br>pH NM	Spherical<br>35 nm	Dilution<br>37°C<br>18 h<br>pH NM<br>$1{\times}10^7$ CFU/ml<br>Ciprofloxacin**	*E. coli*<br>*Acinetobacter baumannii*<br>*P. aeruginosa*<br>*S. aureus*<br>*K. pneumoniae*<br>*P. mirabilis*<br>*Enterococcus faecalis*	75<br>75<br>75<br>75<br>150<br>75<br>37.5 µg/ml	[624]
Acalypha indica	Leaves	Chloroauric acid 1 mM/ plant extract 30 % (1:1 v/v)<br>100°C<br>15 min<br>pH 7	Spherical<br>20 nm	Disc diffusion<br>37°C<br>24 h<br>pH NM<br>$1{\times}10^8$ CFU/ml<br>No control	*S. epidermidis*<br>*E. coli*	31<br>26 mm	[625]
Euphorbia wallichii	Leaves	Chloroauric acid 1 mM/ plant extract 5 % (1:10 v/v)<br>30°C<br>24 h<br>pH NM	Spherical<br>28 nm	Dilution<br>34°C<br>24 h<br>pH NM<br>Inoculum size NM<br>Streptomycin**	*E. coli*<br>*S. aureus*<br>*B. pumilus*<br>*P. aeruginosa*<br>*K. pneumoniae*	21<br>15<br>21<br>17<br>17 mm	[626]

Guazuma ulmifolia	Bark	Chloroauric acid 1 mM / plant extract 10 % (10:1 v/v)<br>Room temperature<br>1 h<br>pH NM	Spherical<br>20–25 nm	Diffusion<br>37°C<br>48 h<br>pH NM<br>Inoculum size NM<br>Neomycin**	*B. subtilis*<br>*S. aureus*<br>*S. pneumoniae*<br>*E. coli*<br>*P. vulgaris*<br>*Shigella dysenteriae*<br>*K. pneumoniae*<br>*P. aeruginosa*	5<br>3<br>7<br>6<br>5<br>0<br>1<br>1 mm	[627]
				Diffusion<br>27°C<br>72 h<br>pH NM<br>Inoculum size NM<br>Amphotricin B**	*Trichoderma viridae*<br>*Fusarium solani*<br>*A. niger*<br>*Nigrospora oryzae*<br>*Aspergillus fumicatus*<br>*C. albicans*	2<br>6<br>4<br>0<br>0<br>0 mm	
Glycyrrhiza uralensis	Roots	Chloroauric acid 1mM/ (1:1 v/v)<br>80°C<br>24 h<br>pH NM	Spherical<br>12 nm	Diffusion<br>30°C<br>24 h<br>pH NM<br>Inoculum size NM<br>Neomycin 30 µg	*S. aureus*<br>*E. coli*<br>*Salmonella enterica*<br>*P. aeruginosa*	13<br>17<br>14<br>11 mm	[628]
Artocarpus heterophyllus	Fruit	Chloroauric acid 2 mM/ plant extract 20 % (1:5 v/v)<br>90°C<br>2 h<br>pH NM	Spherical<br>20–25 nm	Diffusion<br>37°C<br>24 h<br>pH NM<br>Inoculum size NM<br>Ampicillin**<br>Penicillin**<br>Bavistin**	*E. coli*<br>*Streptobacillus spp*	17<br>15 mm	[629]
Allium noeanum	Leaves	Chloroauric acid 1 mM/ plant extract 10 % (10:1 v/v)<br>50°C	Spherical<br>10–30 nm	Dilution<br>37°C<br>24 h<br>pH NM	*S. pneumoniae*<br>*B. subtilis*<br>*S. aureus*<br>*Staphylococcus saprophyticus*	60 µg/ml	[630]

		0.5 h<br>pH NM		Inoculum size NM<br>No control	*S. typhi*<br>*P. aeruginosa*<br>*Shigella flexneri*<br>*E. coli*		
Ribes nigrum	Fruit	Chloroauric acid 0.3 mM/ plant extract 2.5 % (1:1 v/v)<br>90°C<br>24 h<br>pH NM	Hexagonal<br>20 nm	Dilution<br>34°C<br>24–48 h<br>pH NM<br>5×10^5 CFU/ml<br>Amikacin**	*S. aureus* NCTC 4163<br>*P. aeruginosa* NCTC 6749<br>*E. coli* ATCC 25922<br>*P. aeruginosa* K1<br>*S. aureus* K1	13<br>13<br>13<br>13<br>13 µg/ml	[631]
				Dilution<br>34°C<br>pH NM<br>48h<br>$2.5–1\times10^5$ CFU/ml<br>Nystatin**	*C. albicans* ATCC 10231<br>*C. albicans* K1<br>*A. niger* ATCC 16404<br>*Trichophyton rubrum* ATCC 28188	26<br>26<br>26<br>26 µg/ml	
Memecylon umbellatum	Leaves	Chloroauric acid 1 mM/ plant extract 10 % (1:2 v/v)<br>37°C<br>24 h<br>pH NM	Spherical<br>15–25 nm	Diffusion<br>37°C<br>24 h<br>pH NM<br>Inoculum size NM<br>Ofloxacin 10 µg<br>Ciprofloxacin 10 µg<br>Netilmicin 30 µg	*P. aeruginosa*<br>*Streptococcus pyogenes*<br>*Enterococcus faecalis*	10<br>14.5<br>7 mm	[632]
Citrullus lanatus	Rind	Gold (III) chloride 1 mM/ plant extract 50 % (10:1 v/v)<br>100°C<br>1 h<br>pH NM	Spherical<br>20–140 nm	Diffusion<br>37°C<br>24 h<br>pH NM<br>Inoculum size NM<br>Kanamycin 5 µg	*B. cereus* ATCC 13061<br>*E. coli* ATCC 43890<br>*Listeria monocytogenes* ATCC 19115<br>*S. aureus* ATCC 49444<br>*S. typhimurium* ATCC 43174	11<br>9<br>0<br>0<br>11 mm	[633]

Artemisia annua	Leaves	Chloroauric acid 5 mM/ plant extract 2 % (1:1 v/v)<br>Room temperature<br>10 min<br>pH NM	Triangular<br>15–40 nm	Diffusion<br>37°C<br>24 h<br>pH NM<br>Inoculum size NM<br>Ampicillin 100 mg/ml	*E. coli* KCTC-1924<br>*P. aeruginosa* KCTC-2004<br>*Enterococcus aerogenes* KCTC-2190<br>*S. aureus* KCTC-1916<br>*B. cereus* KCTC-1012	18<br>10<br>16<br><br>18<br>23 mm	[634]
Citrus maxima	Peel	Chloroauric acid 1 mM/ plant extract 1% (10:1 v/v)<br>Room temperature<br>24 h<br>pH NM	Spherical<br>8–25 nm	Diffusion<br>37°C<br>24 h<br>pH NM<br>Inoculum size NM<br>Gentamicin**	*S. aureus*<br>*E. coli*	NM<br>NM	[635]
Bauhinia purpurea	Leaves	Chloroauric acid 1 mM/ plant extract 25 % (10:1 v/v)<br>90°C<br>25 min<br>pH NM	Nanorods<br>10–50 nm	Diffusion<br>37°C<br>24–48 h<br>pH NM<br>Inoculum size NM<br>No control	*S. aureus*<br>*B. subtilis*<br>*E. coli*<br>*P. aeruginosa*	8<br>9<br>11<br>10	[636]
				Diffusion<br>Room temperature<br>1 week<br>pH NM<br>Inoculum size NM<br>No control	*A. flavus*<br>*Aspergillus nidulans*	8<br>11 mm	
				Diffusion<br>37°C<br>1 week<br>pH NM<br>Inoculum size NM<br>No control	*Aspergillus fumigatus*<br>*A. niger*	10<br>12.5 mm	

Teraxacum officinale	Leaves	Chloroauric acid 1 mM/ plant extract 10 % (19:1 v/v)<br>70°C<br>10 min<br>pH NM	Cubic, spherical and rod<br>20–40 nm	Diffusion<br>37°C<br>24 h<br>pH NM<br>1×10^6 CFU/ml<br>Penicillin**	*E. coli*<br>*S. aureus*<br>*B. subtilis*<br>*C. albicans*<br>*Saccharomyces cerevisiae*	21<br>19<br>23<br>19<br>18 mm	[637]
Rhodomyrtus tomentosa	Leaves	Chloroauric acid 1 mM/ plant extract 10 % (1:1 v/v)<br>50°C<br>48 h<br>pH NM	Spherical<br>10–20 nm	Dilution<br>37°C<br>24 h<br>pH NM<br>Inoculum size NM<br>No control	*S. aureus* ATCC 29213<br>*E. coli* ATCC 25922	31.25<br>500 µg/ml	[638]
Euphrasia officinalis	Leaves	Chloroauric acid 1 mM/ plant extract 10 % (1:5 v/v)<br>65°C<br>3 h<br>pH NM	Quasi-spherical<br>49 ± 1 nm	Diffusion<br>28°C<br>24 h<br>pH NM<br>Inoculum size NM<br>No control	*P. aeruginosa* KACC 14021<br>*E. coli* CCARM 0237<br>*S. aureus* KCTC 3881<br>*Vibrio parahaemolyticus* KACC 15069	15<br>12<br>14<br>15 mm	[639]
Justicia glauca	Leaves	Chloroauric acid 1 mM/ plant extract 3 % (1:1 v/v)<br>Room temperature<br>10 min<br>pH NM	Hexagonal and spherical<br>32 nm	Dilution<br>37°C<br>24 h<br>pH NM<br>Inoculum size NM<br>Azitromycin 50 µg/ml<br>Claritromycin 50 µg/ml	*Micrococcus luteus*<br>*B. subtilis*<br>*S. aureus*<br>*Streptococcus mutans*<br>*Lactobacillus acidophilus*<br>*E. coli*<br>*P. aeruginosa*<br>*Saccharomyces cerevisiae*<br>*C. albicans*	12.5<br>12.5<br>12.5<br>25<br>25<br>25<br>6.3<br>12.5<br>12.5 µg/ml	[640]
Lonicera japonica	Flowers	Chloroauric acid 1 mM/ plant extract 10 % (19:1 v/v)<br>Room temperature<br>2 h	Triangular<br>8 nm	Diffusion<br>37°C<br>24 h<br>pH NM<br>Inoculum size NM	*E. coli*<br>*B. subtilis*<br>*C. albicans*<br>*Saccharomyces cerevisiae*	21<br>17<br>19<br>15 mm	[641]

		pH NM		Ampicillin**			
Solanum torvum	Fruit	Chloroauric acid 1 mM/ plant extract 10 % (1:4 v/v)<br>Room temperature<br>24 h<br>pH NM	Spherical<br>5–10 nm	Diffusion<br>37°C<br>24 h<br>pH NM<br>Inoculum size NM<br>No control	*E. coli*<br>*P. aeruginosa*<br>*B. subtilis*	12<br>11<br>10 mm	[642]
Terminalia chebula	Seed	Chloroauric acid 10 mM/ plant extract 10 % (1:100 v/v)<br>80°C<br>4 h<br>pH NM	Spherical<br>6–60 nm	Diffusion<br>37°C<br>24 h<br>pH NM<br>1×10^6 CFU/ml<br>Neomycin 45 μg	*S. aureus* ATCC 25923<br>*E. coli* ATCC 25922	NM<br>NM	[643]
Salix alba	Leaves	Chloroauric acid 1 mM/ plant extract 10 % (15:1 v/v)<br>Room temperature<br>30 min<br>pH NM	Spherical<br>50–80 nm	Diffusion<br>37°C<br>18–24 h<br>pH NM<br>Inoculum size NM<br>Streptomycin**	*K. pneumoniae*<br>*B. subtilis*<br>*S. aureus*	0<br>0<br>10	[644]
				Diffusion<br>28°C<br>18–24 h<br>pH NM<br>Inoculum size NM<br>Miconazol**	*Alternaria solani*<br>*A. niger*<br>*A. flavus*	40<br>50<br>10 mm	
Hibiscus cannabinus	Stem	Chloroauric acid 1 mM/ plant extract 5 % (1:1 v/v)<br>Room temperature<br>5 min<br>pH NM	Spherical<br>13 nm	Diffusion<br>37°C<br>24 h<br>pH NM<br>Inoculum size NM<br>No control	*S. aureus*<br>*P. aeruginosa*	13<br>15 mm	[645]

Brassica oleracea	Leaves	Chloroauric acid 1 mM/ plant extract 0.5 % (5:1 v/v)<br>45°C<br>24 h<br>pH NM	Spherical<br>24–38 nm	Diffusion<br>37°C<br>24 h<br>pH NM<br>Inoculum size NM<br>No control	*S. typhi*<br>*E. coli*<br>*S. aureus*<br>*B. subtilis*	9.5<br>9<br>8<br>6 mm	[646]
Solanum nigrum	Leaves	Gold aurochlorate 1 mM/ plant extract 10 % (10:1 v/v)<br>85°C<br>24 h<br>pH NM	Spherical<br>50 nm	Diffusion<br>37°C<br>24 h<br>pH NM<br>Inoculum size NM<br>No control	*B. subtilis*<br>*S. aureus*<br>*E. coli*<br>*P. aeruginosa*	22<br>24<br>21<br>23 mm	[647]
Gymnocladus assamicus	Pod	Chloroauric acid 0.5 mM/ plant extract 1 % (2:1 v/v)<br>60°C<br>1 h<br>pH NM	Spherical<br>4–23 nm	Diffusion<br>37°C<br>24 h<br>pH NM<br>Inoculum size NM<br>No control	*P.aeruginosa* MTCC 2453<br>*S. aureus* MTCC 96	23<br>24 mm	[648]
Carthamus tinctorius	Flowers	Chloroauric acid 2 mM/ plant extract 20 % (9:1 v/v)<br>60°C<br>3–4 h<br>pH NM	Triangular and spherical<br>40–200 nm	Diffusion<br>37°C<br>24 h<br>pH NM<br>Inoculum size NM<br>Imipenem**<br>Norfloxacin**<br>Vancomycin **	*E. coli*<br>*Streptobacillus spp*<br>*A. niger*<br>*A. flavus*	30<br>21<br>0<br>30 mm	[649]
Embelia ribes	Seed	Chloroauric acid 1 mM/ plant extract 10 % (10:1 v/v)<br>100°C<br>25 min<br>pH NM	Spherical<br>10–30 nm	Diffusion<br>37°C<br>24 h<br>pH NM<br>Inoculum size NM<br>No control	*E. coli*<br>*S. aureus*	28<br>22 mm	[650]

Trianthema decandra	Roots	Chloroauric acid 1 mM/ plant extract 20 % (1:1 v/v)<br>37°C<br>24 h<br>pH NM	Spherical<br>33–65 nm	Diffusion<br>37°C<br>24–48 h<br>pH NM<br>1×10^6 CFU/ml<br>Chloramphenicol**	*S. aureus* MTCC 29213<br>*Streptococcus faecalis* MTCC 0459<br>*Enterococcus faecalis* MTCC 2729<br>*E. coli* MTCC 443<br>*P. aeruginosa* MTCC 1035<br>*Proteus vulgaris* MTCC 1771<br>*B. subtilis* MTCC 121<br>*Yersinia enterocolitica* MTCC 840	14.5<br>13.5<br>10<br>9.5<br>11.5<br>15<br>9.5<br>15.5 mm	[651]
				Diffusion<br>37°C<br>24–48 h<br>pH NM<br>1×10^6 CFU/ml<br>Nystatin**	*C. albicans* MTCC 183	8.5 mm	
Rivea hypocrateriformis	Aerial parts	Chloroauric acid 1 mM/ plant extract 5 % (5:2 v/v)<br>40°C<br>1 h<br>pH NM	Spherical<br>20–30 nm	Diffusion<br>37°C<br>24 h<br>pH NM<br>1×10^6 CFU/ml<br>Ciprofloxacin 100 µg/ml	*S. aureus* MTCC 7443<br>*K. pneumoniae* MTCC 7407<br>*E. coli* MTCC 7410<br>*B. subtilis* MTCC 121<br>*P. aeruginosa* MTCC 7903	8<br>7<br>9<br>9<br>6 mm	[652]
				Diffusion<br>37°C<br>24 h<br>pH NM<br>1×10^6 CFU/ml<br>Fluconazole 100 µg/ml	*C. albicans* MTCC 227<br>*Chrysosporium indicum* MTCC 4266<br>*Trichopyton rubrum* MTCC 296	6<br>6<br>5 mm	

Jasminum sambac	Leaves	Chloroauric acid 1 mM/ plant extract 5 % (5:1 v/v)<br>80°C<br>12 h<br>pH NM	Spherical<br>20–50 nm	Diffusion<br>37 °C<br>24–48 h<br>pH NM<br>1×10^{8} CFU/ml<br>Kanamycin 10 µg<br>Ampicillin 10 µg<br>Erythromycin 10 µg<br>Ciprofloxacin 10 µg	*S. aureus*<br>*E. coli*<br>*S. typhi*<br>*P. aeruginosa*	18<br>26<br>24<br>21 mm	[653]
				Diffusion<br>37 °C<br>24–48 h<br>pH NM<br>1×10^{8} CFU/ml<br>Fluconazol 10 µg<br>Imidazol 10 µg	*Trichophyton rubrum*<br>*C. albicans*<br>*Candida indicum*	9<br>11<br>8 mm	
Albizia amara	Leaves	Chloroauric acid 1 mM / plant extract 5 % (10:1 v/v)<br>30°C<br>2 h<br>pH NM	Hexagonal<br>34–64 nm	Diffusion<br>37 °C<br>24 h<br>pH 7.4<br>Inoculum size NM<br>Ciprofloxacin**	*S. aureus* MTCC 96	16 mm	[654]
Ananas comosus	Peel	Chloroauric acid 2 mM/ plant extract 20 % (2:5 v/v)<br>90°C<br>20 min<br>pH NM	Spherical<br>10 ± 5 nm	Diffusion<br>37°C<br>24 h<br>pH NM<br>Inoculum size NM<br>Ampicillin**<br>Penicillin**	*E. coli*<br>*Streptobacillus spp*	14<br>17 mm	[655]
				Diffusion<br>37°C<br>48 h	*A. niger*<br>*A. flavus*	14<br>14 mm	

				pH NM<br>Inoculum size NM<br>No control			
Achillea wilhelmsii	Leaves	Chloroauric acid 1 mM/ plant extract 5 % (9:1 v/v)<br>60°C<br>15 min<br>pH NM	Spherical<br>2.7–38.7 nm	Diffusion<br>37°C<br>24 h<br>pH NM<br>1×10^8 CFU/ml<br>Ampicillin**<br>Penicillin**<br>Bavistin**	*E. coli*<br>*Salmonella enterica*<br>*S. epidermidis*<br>*B. subtilis*	0<br>0<br>10<br>11 mm	[656]
Cerasus serrulata	Leaves	Chloroauric acid 0.5 mM/ plant extract 1 % (5:1 v/v)<br>Room temperature<br>20 min<br>pH NM	Spherical<br>5–25 nm	Diffusion<br>37°C<br>24 h<br>pH NM<br>5×10^5 CFU/ml<br>No control	*E. coli*<br>*S. aureus*	16<br>15 mm	[657]
Tamarindus indica	Seeds	Chloroauric acid 1 mM/ plant extract 5 % (1:4 v/v)<br>Room temperature<br>24 h<br>pH NM	Spherical<br>8 nm	Diffusion<br>37°C<br>24 h<br>pH NM<br>5×10^5 CFU/ml<br>Streptomycin**	*K. pneumoniae*<br>*B. subtilis*<br>*S. epidermidis*	10<br>12<br>10 mm	[658]
Turnera diffusa	Leaves	Chloroauric acid 1 mM/ plant extract 1 % (9:1 v/v)<br>60°C<br>4 h<br>pH NM	Spherical<br>24 nm	Diffusion<br>37°C<br>24 h<br>pH NM<br>Inoculum size NM<br>No control	*Vibrio parahaemolyticus*<br>*Aeromonas hydrophila*	15<br>17 mm	[659]
Cotoneaster horizontalis	Leaves	Chloroauric acid 1 mM/ plant extract 10 % (7:5 v/v)	Spherical<br>18 ± 2 nm	Diffusion<br>37°C<br>24 h	*P. aeruginosa*	17 mm	[660]

		37°C<br>24 h<br>pH NM		pH NM<br>Inoculum size NM<br>No control			
Combretum erythrophyllum	Leaves	Chloroauric acid 1 mM/ plant extract 5 % (25:1 v/v)<br>90°C<br>1 h<br>pH NM	Spherical<br>13.20 nm	Dilution<br>37°C<br>24 h<br>pH NM<br>1×10^8 CFU/ml<br>Streptomycin 1 mg/ml	*Staphylococcus epidermidis* ATCC 14990<br>*S. aureus* ATCC 25923<br>*Mycobacterium smegmatis* MC 215<br>*Proteus mirabilis* ATCC 7002<br>*E. coli* ATCC 25922<br>*K. pneumoniae* ATCC 13822<br>*Klebsiella oxytoca* ATCC 8724	62.5<br>62.5<br>62.5<br>62.5<br>62.5<br>62.5<br>62.5 µg/ml	[661]
Tragopogon dubius	Leaves	Chloroauric acid 1 mM/ plant extract 10 % (10:1 v/v)<br>Room temperature<br>24 h<br>pH NM	Spherical<br>34 ± 3 nm	Diffusion<br>37°C<br>24 h<br>pH NM<br>Inoculum size NM<br>Tetracycline 30 µg	*B. cereus* ATCC 14579<br>*E. coli* ATCC 35218<br>*S. aureus* ATCC 6538<br>*K. pneumoniae* ATCC 700603	25<br>12.5<br>26<br>13 mm	[662]
Petroselinum crispum	Leaves	Chloroauric acid 1 mM/ plant extract 10 % (1:1 v/v)<br>90°C<br>20 min<br>pH NM	Spherical<br>17 nm	Diffusion<br>37°C<br>48 h<br>pH NM<br>Inoculum size NM<br>No control	*B. subtilis*<br>*Enterococcus faecalis*<br>*E. coli*<br>*Enterobacter ludwigii*	0<br>0<br>4<br>3.2 mm	[663]
Pimenta dioica	Leaves	Chloroauric acid 1 mM/ plant extract 50 % (3:2 v/v)<br>30°C<br>24 h<br>pH NM	Spherical<br>17 nm	Diffusion<br>37°C<br>24 h<br>pH NM<br>Inoculum size NM<br>No control	*S. aureus*<br>*E. coli*	4<br>9 mm	[664]

Morchella esculenta	Mushroom	Chloroauric acid 1 mM/ plant extract 20 % (5:1 v/v)<br>75°C<br>48 h<br>pH NM	Spherical<br>16.51 nm	Dilution<br>37°C<br>24 h<br>pH NM<br>1×10^8 CFU/ml<br>Vancomycin**<br>Colistin**	*S. aureus*<br>*B. subtilis*<br>*E. coli*	0.4<br>0.9<br>0.2 µg/ml	[665]
				Dilution<br>37°C<br>24 h<br>pH NM<br>1×10^8 CFU/ml<br>Fluconazole**	*C. albicans*	0.1 µg/ml	
Cynodon dactylon	Leaves	Chloroauric acid 1 mM/ plant extract 1 % (4:1 v/v)<br>Room<br>2 h<br>pH NM	Spherical<br>21.33 nm	Diffusion<br>37°C<br>24 h<br>pH NM<br>5×10^6 CFU/ml<br>Ciprofloxacin**	*Enterobacter cloacae*<br>*Staphylococus haemolytics*<br>*Staphylococcus petrasii*<br>*B. cereus*	13<br>12<br>13<br>12 mm	[666]
Peganum harmala	Leaves	Chloroauric acid/ plant extract 2 % (1:50 w/v)<br>Room temperature<br>2 h<br>pH NM	Spherical<br>43 nm	Diffusion<br>37°C<br>24 h<br>pH NM<br>Inoculum size NM<br>Cefoxitin**<br>Chloramphenicol**	*E. coli*<br>*S. aureus*	25<br>30 mm	[667]
Rhazya stricta decne	Leaves	Chloroauric acid 2 mM/ plant extract 12 % (5:1 v/v)<br>24°C<br>4 h<br>pH NM	Spherical<br>40 nm	Diffusion<br>37 °C<br>24 h<br>pH NM<br>Inoculum size NM<br>No control	*E. coli*<br>*B. subtilis*	18<br>12 mm	[668]

Actinidia deliciosa	Fruit	Chloroauric acid 1 mM/ plant extract 10 % (9:1 v/v)<br>80°C<br>1 h<br>pH NM	Spherical<br>25–40 nm	Diffusion<br>37°C<br>24 h<br>pH NM<br>Inoculum size NM<br>No control	*P. aeruginosa*	NM	[669]
Angelica pubescens	Roots	Chloroauric acid 5 mM/ plant extract 5 % (2:1 v/v)<br>80°C<br>50 min<br>pH NM	Spherical<br>7 nm	Diffusion<br>37°C<br>24 h<br>pH NM<br>Inoculum size NM<br>Neomycin 30 μg	*E. coli* ATCC 10798<br>*S. aureus* ATCC 6538<br>*P. aeruginosa* ATCC 27853<br>*Salmonella enterica* ATCC 13076	14<br>17<br>13<br>12 mm	[670]
Prunus armeniaca	Gum	Chloroauric acid 1 mM/ plant extract 0.5 % (8:5 v/v)<br>80°C<br>10 min<br>pH NM	Spherical<br>5–40 nm	Diffusion<br>37°C<br>24 h<br>pH NM<br>Inoculum size NM<br>Streptomycin**	*S. aureus* ATCC 25923<br>*E. coli* ATCC 25922<br>*P. aeruginosa* ATCC 27853	10<br>9<br>8 mm	[671]
Senna siamea	Seed	Chloroauric acid 2 mM/ plant extract 16 % (5:2 v/v)<br>70°C<br>3 h<br>pH NM	Nearly triangular<br>50–70 nm	Diffusion<br>37°C<br>24 h<br>pH NM<br>Inoculum size NM<br>No control	*S. aureus*<br>*B. subtilis*<br>*P. aeruginosa*<br>*K. pneumoniae*<br>*E. coli*	13<br>18<br>15<br>21<br>15 mm	[672]
Ginkgo biloba	Leaves	Chloroauric acid 1 mM/ plant extract 33 % (2:5 v/v)<br>40°C<br>12 h<br>pH NM	Spherical<br>10–30 nm	Diffusion<br>37°C<br>24 h<br>pH NM<br>Inoculum size NM<br>No control	*Agrobacterium tumefaciens*<br>*B. subtilis*<br>*E. coli*	15<br>19<br>19 mm	[673]

Ficus benghalensis	Leaves	Chloroauric acid 1 mM/ plant extract 10 % (2:1 v/v)<br>30°C<br>5–7 min<br>pH NM	Spherical<br>20 nm	Diffusion<br>37°C<br>24 h<br>pH NM<br>Inoculum size NM<br>No control	*E. coli*<br>*B. subtilis*<br>*S. aureus*<br>*K. pneumonia*	12<br>8<br>16<br>10 mm	[674]
Evolvulus alsinoides	Leaves	Chloroauric acid 1 mM/ plant extract 10 % (9:1 v/v)<br>60°C<br>15 min<br>pH NM	Spherical<br>80 nm	Diffusion<br>37°C<br>24–48 h<br>pH NM<br>Inoculum size NM<br>Methicillin 10 µg<br>Itraconazol 10 µg	*Aeromonas liquefaciens* MTCC 2645<br>*Enterococcus faecalis* MTCC 439<br>*K. pneumoniae* NCIM 2883<br>*Micrococcus luteus* NCIM 2871<br>*S. typhimurium* NCIM 2501<br>*Vibrio cholera* MTCC 3906	13<br>17<br>16<br>18<br>14<br>15 mm	[675]
				Diffusion<br>25°C<br>48–72 h<br>pH NM<br>Inoculum size NM<br>Itraconazol 10 µg	*C. albicans* MTCC 1637<br>*Cryptococcus spp* MTCC 7076<br>*Microsporum canis* MTCC 3270<br>*Trichophyton rubrum* MTCC 3272	10<br>9<br>9<br>7 mm	
Abelmoschus esculentus	Seed	Chloroauric acid 1 mM/ plant extract 2 % (3:2 v/v)<br>Room temperature<br>10 min<br>pH NM	Spherical<br>45–75 nm	Diffusion<br>25°C<br>72 h<br>pH NM<br>Inoculum size NM<br>Amphotericin B**	*Puccinia graminis*<br>*A. flavus*<br>*A. niger*<br>*C. albicans*	17<br>15<br>16<br>18 mm	[676]
Rumex hymenosepalus	Roots	Tetrachlauric acid 10 mM/ plant extact 1 % (1:1 v/v)<br>Room temperature<br>1 h<br>pH NM	Spherical<br>24 nm	Dilution<br>37°C<br>24 h<br>pH NM<br>1×10^8 CFU/ml<br>No control	*E. coli* ATCC 25922<br>*S. aureus* ATCC 5538	50<br>50 µg/ml	[677]

Capsicum annuum	Fruits	Tetrachloroauric acid 5 mM/ plant extract 8 % (5:1 v/v)<br>Room temperature<br>Incubation time<br>pH NM	Spherical<br>35.1 nm	Diffusion<br>37°C<br>48 h<br>pH NM<br>Inoculum size NM<br>Chloramphenicol**<br>Difloxacin**	*Pasteurella multocida*<br>*Klebsiella rhinoscleromatis*<br>*Streptococcus pyogenes*<br>*Vibrio vulnificus*	32<br>28<br>18<br>33 mm	[678]
Asparagus racemosus	Roots	Tetrachloroauric acid 1 mM/ plant extract 10 % (10:1 v/v)<br>Room temperature<br>24 h<br>pH NM	Spherical<br>10–50 nm	Diffusion<br>37°C<br>24 h<br>pH NM<br>1×10^5 CFU/ml<br>Gentamycin**<br>Streptomycin **	*P. aeruginosa* ATCC 27853<br>*S. aureus* ATCC 25923	26<br>23 mm	[679]
Euphorbia hirta	Leaves	Chloroauric acid 1 mM/ plant extract 10 % (1:1 v/v)<br>37°C<br>5 min<br>pH NM	Spherical<br>6–71 nm	Dilution<br>37°C<br>24 h<br>pH NM<br>Inoculum size NM<br>No control	*E. coli*<br>*P. aeruginosa*<br>*K. pneumoniae*	88<br>86<br>94 %	[680]
Acorus calamus	Rhizome	Chloroauric acid 1 mM/ plant extract 30 % (1:1 v/v)<br>100°C<br>15 min<br>pH 4	Spherical<br>10 nm	Dilution<br>37°C<br>24–48 h<br>pH NM<br>Inoculum size NM<br>No control	*S. aureus* MTCC 96<br>*E. coli* MTCC 1671	63<br>80 %	[681]
Chenopodium formosanum	Shell	Chloroauric acid 1 mM/ plant extract 1 % (1:2 v/v)<br>25°C<br>1 h<br>pH NM	Spherical<br>8 ± 6 nm	Dilution<br>37°C<br>18 h<br>pH NM<br>1×10^6 CFU/ml<br>Streptomycin 40 μg	*E. coli*<br>*S. aureus*	85<br>60 %	[682]

Avicennia marina	Seeds	Chloroauric acid 1mM/ plant extract 20 % (20:1 v/v)<br>40°C<br>24 h<br>pH NM	Spherical<br>10-15 nm	Dilution<br>35°C<br>24 h<br>pH NM<br>Inoculum size NM<br>Neomycin**	*E. coli*<br>*K. pneumoniae*<br>*S. aureus*<br>*P. aeruginosa*<br>*Enterococcus faecalis*	200<br>200<br>200<br>200 µg/ml<br>NM	[683]
Chenopodium quinoa	Seed	Chloroauric acid 5 mM/ plant extract 4 % (1:4 v/v)<br>82°C<br>30 min<br>pH NM	Spherical<br>5.5 nm	Dilution<br>37°C<br>24 h<br>pH NM<br>1×10^6 CFU/ml<br>No control	*E. coli* ATCC 25972<br>*S. aureus* ATCC 1901	99<br>98 %	[684]
Acer pentapomicum	Leaves	Chloroauric acid 1 mM/ plant extract 10 % (10:1 v/v)<br>Room temperature<br>24 h<br>pH NM	Spherical<br>19–24 nm	Diffusion<br>37°C<br>24 h<br>pH NM<br>Inoculum size NM<br>No control	*B. subtilus*<br>*Citrobacter freundi*<br>*E. coli*<br>*K. pneumoniae*<br>*P. aeruginosa*<br>*S. aureus*<br>*Xanthomonas compestris*	27<br>42<br>38<br>81<br>40<br>38<br>32 mm	[685]
Panax ginseng	Berries	Gold (III) chloride 1 mM/ plant extract 6 % (1:1 v/v)<br>80°C<br>30 min	Spherical<br>5-10 nm	Diffusion<br>37°C<br>24 h<br>pH NM<br>Inoculum size NM<br>Neomycin**	*E. coli*<br>*S. aureus*	11<br>12.3 mm	[686]
Clitoria ternatea	Leaves	Chloroauric acid 1 mM / plant extract 20 %<br>(2:5 v/v)<br>Room temperature	Rod<br>100 nm	Diffusion<br>37°C<br>24 h<br>pH NM<br>Inoculum size NM	*S. aureus*<br>*S. epidermidis*<br>*E. coli*<br>*P. aeruginosa*	NM	[687]

		24 h		No control			
Pleurotus ostreatus	Basidiocarps	Chloroauric acid 1 mM/plant extract 8 % (5:1 v/v)<br>Room temperature<br>20 min	Spherical<br>22.9 nm	Diffusion<br>37°C<br>24 h<br>pH NM<br>Inoculum size NM<br>Tetracycline**	*Enterococcus faecalis* ATCC 29212<br>*E. coli* ATCC 25922<br>*K. pneumonia* ATCC 700603<br>*P. aeruginosa* ATCC 254992<br>*S. aureus* ATCC 254996	0<br>0<br>0<br>29<br>12 mm	[688]
				Diffusion<br>37°C<br>48 h<br>pH NM<br>Inoculum size NM<br>Fluconazole**	*C. albicans* ATCC 10231	32 mm	
Cajanus cajan	Leaves	Chloroauric acid 1 mM/ plant extract 10 % (9:1 v/v)<br>60°C<br>10 min<br>pH NM	Spherical<br>1-100 nm	Diffusion<br>37°C<br>24 h<br>pH NM<br>1×10^2 CFU/ml<br>No control	*Bacillus spp*<br>*Pseudomonas spp*	33<br>29 mm	[689]
Terminalia bellirica	Fruit	Chloroauric acid 1 mM/ plant extract 5 % (9:1 v/v)<br>Room temperature<br>20 min<br>pH NM	Spherical, triangular, hexagonal<br>20-30 nm	Diffusion<br>37°C<br>18 h<br>pH NM<br>Inoculum size NM<br>No control	*E. coli*	14 mm	[690]
				Diffusion<br>37°C<br>72 h<br>pH NM<br>Inoculum size NM<br>No control	*C. albicans*<br>*Candida tropicalis*	16<br>13 mm	

Achyranthes aspera	Leaves	Chloroauric acid 1 mM/ plant extract 10 % (1:1 v/v)<br>Room temperature<br>24 h<br>pH NM	Spherical<br>18 nm	Diffusion<br>37°C<br>24 h<br>pH NM<br>Inoculum size NM<br>No control	*Streptocccoccus mutans*	18.5 mm	[691]
Plumbago zeylanica	Leaves	Chloroauric acid 1 mM/ plant extract 2 % (1:1 v/v)<br>Room<br>24 h<br>pH NM	Spherical<br>16.89 nm	Diffusion<br>37°C<br>16–18 h<br>pH NM<br>Inoculum size NM<br>Netilmicin**	*E. coli*<br>*P. aeruginosa*<br>*B. subtilis*<br>*S. aureus*	8<br>11<br>12<br>8 mm	[692]
				Diffusion<br>35°C<br>48 h<br>pH NM<br>Inoculum size NM<br>Fluconazole**	*Candida tropicalis*<br>*A. flavus*	8<br>0 mm	
Carica papaya	Leaves	Chloroauric acid 1 mM/ plant extract 1 % (1:4 v/v)<br>55°C<br>30 min<br>pH NM	Spherical<br>15±2 nm	Diffusion<br>37°C<br>24 h<br>pH NM<br>1×10^8 CFU/ml<br>Ampicillin**	*S. aureus*<br>*Pseudomonas putida*	NM<br>NM	[693]
Garcinia kola	Pulp	Chloroauric acid 1 mM/ plant extract 20 % (4:1 v/v)<br>Room<br>4 h<br>pH NM	Spherical<br>28 nm	Diffusion<br>37°C<br>22 h<br>pH NM<br>1×10^8 CFU/ml<br>Amoxicillin**	*P. aeruginosa*<br>*E. coli*<br>*S. aureus*<br>*B. cereus*	11<br>12<br>4<br>7 mm	[694]

Terminalia arjuna	Leaves	Chloroauric acid 1 mM/ plant extract 5 % (20:1 v/v)<br>60°C<br>30 min<br>pH NM	Spherical<br>20 nm	Diffusion<br>37°C<br>24 h<br>pH NM<br>Inoculum size NM<br>No control	*S. aureus* NCIM 5021<br>*P. aeruginosa* NCIM 5029<br>*Salmonella typhimurium* NCIM 2501	20<br>18<br>16 mm	[695]
Populus alba	Leaves	Chloroauric acid 1 mM/ plant extract 5 % (9:1 v/v)<br>85°C<br>15 min<br>pH NM	Spherical<br>16.3 nm	Dilution<br>37°C<br>24 h<br>pH NM<br>Inoculum size NM<br>No control	*S. aureus*<br>*E. coli*	100<br>100 µg/ml	[696]
Curcuma pseudomontana	Rhizome	Chloroauric acid 1 mM/ plant extract 1 % (20:1 v/v)<br>Room<br>30 min<br>pH NM	Spherical<br>16.3 nm	Diffusion<br>37°C<br>24 h<br>pH NM<br>1×10^8 CFU/ml<br>Streptomycin**	*S. aureus*<br>*E. coli*<br>*B. subtilis*<br>*P. aeruginosa*	25<br>28<br>26<br>23 mm	[697]
Allium ampeloprasum	Leaves	Chloroauric acid 1 mM/ plant extract 10 % (10:1 v/v)<br>80°C<br>48 h<br>pH NM	Spherical<br>96 nm	Dilution<br>37°C<br>24 h<br>pH NM<br>Inoculum size NM<br>Colistin**<br>Vancomycin**	*E. coli* ATCC 25922<br>*S. aureus* ATCC 29213<br>*B. subtillis* ATCC 11774<br>*P. aeruginosa* ATCC 27853	0.5<br>0.1<br>0.3<br>1 mg/ml	[698]
				Dilution<br>37°C<br>24 h<br>pH NM<br>Inoculum size NM<br>Fluconazole**	*C. albicans*	0.1 mg/ml	

Glycyrrhzia glabra	Roots	Chloroauric acid 1 mM/ plant extract 2 % (6:1 v/v)<br>25°C<br>2.5 h<br>pH 5	Spherical<br>55 nm	Diffusion<br>37°C<br>24 h<br>pH NM<br>Inoculum size NM<br>Norfloxacin**	*B. subtillis*<br>*S. aureus*<br>*E. coli*<br>*P. aeruginosa*<br>*S. typhi*	25<br>26<br>29<br>25<br>26 mm	[699]
				Diffusion<br>37°C<br>72 h<br>pH NM<br>Inoculum size NM<br>Nystatin**	*A. niger*<br>*C. albicans*<br>*Fusarium oxyporum*<br>*A. flavus*<br>*Penicillium citrinum*	14<br>17<br>16<br>18<br>19 mm	
Viola betonicifolia	Leaves	Chloroauric acid 1 mM / plant extract 15 % (1:1 v/v)<br>40°C<br>60 min<br>pH NM	Spherical<br>13 nm	Dilution<br>37°C<br>24 h<br>pH NM<br>1×10^5 CFU/ml<br>No control	*S. aureus* ATCC 23235<br>*B. subtilis* ATCC 6051<br>*E. coli* ATCC 25922<br>*P. aeruginosa* ATCC 27853	89<br>87<br>92<br>93 %	[700]
				Dilution<br>30°C<br>24 h<br>pH NM<br>1×10^5 CFU/ml<br>No control	*C. albicans* ATCC 10231<br>*Aspergillus fumigatus* ATCC 13073<br>*A. flavus* ATCC 9643<br>*A. niger* ATCC 11414	69<br>76<br><br>85<br>70 %	

*MIC=minimal inhibition concentration; ZOI=zone of inhibition; PI=percentage of inhibition

**The quantity or concentration is not mentioned.

NM=not mentioned, MRSA=methicillin-resistant *S. aureus*

Table S3. Antimicrobial green synthesized zinc (oxide) nanoparticles

**Plant type**	**Part used**	**Operative conditions for synthesis**	**NP characteristics (shape and size)**	**Microbiological analyses (operative conditions)**			**Ref.**
				**Methods,<br>incubation temperature,<br>incubation time,<br>pH,<br>inoculum density,<br>positive control**	**Tested bacteria**	**MIC, ZOI or PI***	
Veronica multifida	Leaves	Zinc acetate/ plant extract 2 %<br>(1:100 w/v)<br>50 °C<br>15 min<br>pH 12	Hexagonal and quasi-spherical<br>10–100 nm	Diffusion<br>35°C<br>16–20 h<br>pH NM<br>1.5×10^8 CFU/ml<br>Penicillin G 10 mg<br>Gentamicin 10 mg<br>Tetracycline 30 mg	*E. coli* ATCC 43895<br>*S. aureus* ATCC 29213<br>*B. subtilis*<br>*Bacillus licheniformis*<br>*P. aeruginosa*<br>*S. typhi*	12<br>8<br>15<br>13<br>15<br>14 mm	[701]
Geranium wallichianum	Leaves	Zinc nitrate 1 mM/ plant extract 4 % (3:50 w/v)<br>60 °C<br>3 h<br>pH 12	Hexagonal<br>18 nm	Diffusion<br>37°C<br>24 h<br>pH NM<br>Inoculum size NM<br>Oxytetracycline 10 µg	*B. subtilis* ATCC 6633<br>*S. aureus* ATCC 25923<br>*P. aeruginosa* ATCC 9721<br>*E. coli* ATCC15224<br>*K. pneumoniae* ATCC 4617	30<br>18<br>22<br>10<br>15 mm	[702]

				Diffusion<br>28°C<br>48 h<br>pH NM<br>Inoculum size NM<br>Amphotericin B**	*A. flavus* FCBP 0064<br>*A.niger* FCBP 0918<br>*C. albicans* FCBP 478<br>*Fusarium solani* FCBP 0291<br>*Mucor racemosus* FCBP 0300	10<br>20<br>12<br>10<br>14 mm	
Pelargonium graveolens	Leaves	Zinc acetate/ plant extract 10 % (1:1 w/v)<br>100°C<br>20 min<br>pH NM	Spherical<br>60–80 nm	Diffusion<br>37°C<br>24 h<br>pH NM<br>$2×10^5$ CFU/ml<br>Amoxycillin**	*E. coli*<br>*S. aureus*	18<br>12 mm	[703]
Capsicum annuum	Fruit	Zinc acetate 5 mM/ plant extract 2 % (4 :1 v/v)<br>450 °C<br>30 min<br>pH NM	Hexagonal<br>30–40 nm	Diffusion<br>35 ± 1 °C<br>36 h<br>pH NM<br>$5×10^6$ CFU/ml<br>Gentamicin**	*E. coli* MTCC 443<br>*S. aureus* MTCC 5823	8<br>6 mm	[704]
				Diffusion<br>28 ± 1°C<br>72 h<br>pH NM<br>$3×10^5$ CFU/ml<br>Fluconazol**	*Humicola fuscoatra* MTCC 3938	4 mm	
Periploca aphylla	Plant	Zinc acetate/ plant extract 12.5 % (1:50 w/v)<br>45 °C<br>25 h<br>pH NM	Spherical<br>6 nm	Diffusion<br>37°C<br>24 h<br>pH NM<br>$1.5×10^8$ CFU/ml<br>Cefixime 20 µg/ml	*E. coli* ATCC 2340<br>*Serratia marcescens* ATCC 43297<br>*Enterobacter cloacae* ATCC 2341	23<br>19<br>24.5 mm	[705]
Citrullus colocynthis	Fruit	Zinc nitrate/ plant extract 10 % (3:5 w/v)<br>100 °C<br>8 min	Hexagonal<br>27–85 nm	Diffusion<br>37°C<br>24 h<br>pH NM<br>Inoculum size NM	*B. subtilis*<br>MRSA<br>*P. aeruginosa*<br>*E. coli*	14.3<br>7<br>12.2<br>13.4 mm	[706]

		pH NM		No control			
Catharanthus roseus	Leaves	Zinc acetate 10 mM/ plant extract 12 % (50:1 v/v)<br>40°C<br>30 min<br>pH 12	Spherical<br>62–94 nm	Diffusion<br>30°C<br>24h<br>pH NM<br>1×10^8 CFU/ml<br>Streptomycin 10 µg/ml	*S. aureus* MTCC 9760<br>*B. cereus* MTCC 430<br>*P. aeruginosa* MTCC 424<br>*P. mirabilis* MTCC 3310<br>*E. coli* MTCC 40<br>*Streptococcus pyogenes* MTCC 1926	12<br>11.5<br>11<br>11<br>11<br>11.5 mm	[707]
Hibiscus subdariffa	Leaves	Zinc acetate 91 mM /20 ml of plant extract 5 % (5:2 v/v)<br>50 °C<br>10 min<br>pH 10	Spherical<br>10–60 nm	Dilution<br>37°C<br>24 h<br>pH 7<br>1×10^7CFU/ml<br>No control	*E. coli* MTCC 1652<br>*S. aureus* MTCC 96	50<br>50 µg/ml	[708]
Moringa oleifera	Leaves	Zinc nitrate/ plant extract 20 % (1:5 w/v)<br>60–80 °C<br>1 h<br>pH NM	Spherical<br>16–20 nm	Diffusion<br>37°C<br>24 h<br>pH NM<br>Inoculum size NM<br>Ciprofloxacin 5 µg	*S. aureus*<br>*B. substilis*<br>*P. aeruginosa*<br>*Proteus mirabis*<br>*E.coli*	22.5<br>21.5<br>20<br>19<br>20 mm	[709]
				Diffusion<br>28–35°C<br>24h<br>pH NM<br>Inoculum size NM<br>Amphotercin B 10 µg	*C. albicans*<br>*Candida tropicalis*	20<br>18 mm	
Solanum nigrum	Leaves	Zinc nitrate /plant extract 20 % (1:10 w/v)<br>60°C<br>2 h<br>pH NM	Spherical<br>20–30 nm	Diffusion<br>37°C<br>18–24 h<br>pH NM<br>Inoculum size NM<br>No control	*S. aureus*<br>*Salmonella paratyphi*<br>*Vibrio cholerae*<br>*E. coli*	18<br>17<br>11<br>7 mm	[710]

Petroselum crispum	Leaves	Zinc acetate/ plant extract 10 % (1:10 w/v)<br>90°C<br>72 h<br>pH NM	Spherical<br>40–50 nm	Diffusion<br>37°C<br>24 h<br>pH NM<br>Inoculum size NM<br>No control	*E. coli*	4.3 mm	[711]
Sarcopoterium spinosum	Leaves	Zinc nitrate 1 mM/ plant extract 3 % (1:1 v/v)<br>60°C<br>2 h<br>pH 10	Spherical<br>26–115 nm	Dilution<br>37°C<br>24 h<br>pH 10<br>Inoculum size NM<br>Ampicillin**	*E. coli* ATCC 25922<br>*P. aeruginosa* ATCC 27853<br>*S. aureus* ATCC 25925<br>*B. subtilis* ATCC 6633<br>*Enterococcus faecalis* ATCC 29212<br>*Streptococcus pneumoniae* ATCC 10353	62.5<br>125<br>125<br>62.5<br>62.5<br><br>125 µg/ml	[712]
				Dilution<br>28°C<br>24 h<br>pH 10<br>Inoculum size NM<br>Fluconazol**	*Candida glabrata* ATCC 4322<br>*C. albicans* ATCC 90028	31.3<br>31.3 µg/ml	
Couroupita guianensis	Leaves	Zinc acetate 25 mM/ plant extract 5 % (25:2 v/v)<br>70°C<br>10 min<br>pH NM	Hexagonal<br>1–100 nm	Diffusion<br>37°C<br>24 h<br>pH NM<br>Inoculum size NM<br>No control	*B. cereus*<br>*K. pneumoniae*<br>*E. coli*<br>*Micrococcus luteus*<br>*S. typhi*<br>*Vibrio cholerae*	17<br>18<br>19<br>15<br>18<br>19 mm	[713]
Nephelium lappaceum	Peel	Zinc nitrate 100 mM/ plant extract 10 % (1:10 v/v)<br>80°C<br>2 h<br>pH 12	Needle nanocrystals<br>50 nm	Diffusion<br>37°C<br>24 h<br>pH NM<br>Inoculum size NM<br>Ampicillin 10 µg<br>Gentamicin 10 µg<br>Nalidixic acid 30 µg<br>Chloromphenicol 30 µg	*E. coli* ATCC 11632<br>*S. aureus* ATCC 10536	18<br>23 mm	[714]

				Cefalexin 30 µg<br>Co trimoxazole 25 µg<br>Penicillin G 10 µg<br>Vancomycin 30 µg<br>Ampicillin 10 µg<br>Oxacillin 1 µg<br>Erythromycin 15 µg<br>Methicillin 5 µg			
Rosa canina	Fruit	Zinc nitrate 50 mM/ plant extract 10 % (1:2 v/v)<br>150°C<br>5 h<br>pH 6	Spherical<br>50 nm	Difffusion<br>37°C<br>24 h<br>pH NM<br>Inoculum size NM<br>No control	*E. coli*<br>*Salmonella typhimurium*<br>*Listeria monocytogenes*<br>*S. aureus*	8<br>00<br>10<br>11 mm	[715]
Terminalia arjuna	Bark	Zinc acetate 1000 mM/ plant extract 10 % (1:10 v/v)<br>80°C<br>8 h<br>pH NM	Spherical<br>21 nm	Diffusion<br>37°C<br>18–24 h<br>pH NM<br>Inoculum size NM<br>No control	*S. aureus* ATCC 6538<br>*E. coli* ATCC 9677	22.5<br>24 mm	[716]
Glycosmis pentaphylla	Leaves	Zinc acetate 200 mM/ plant extract 10 % (1:2 v/v)<br>60°C<br>1 h<br>pH NM	Hexagonal<br>32–36 nm	Diffusion<br>37°C<br>24–48 h<br>pH NM<br>Inoculum size NM<br>Ciprofloxacin 5 µg	*Shigella dysenteriae*<br>*B. cereus* MTCC 430<br>*Salmonella paratyphi* MTCC 735<br>*S. aureus* MTCC 737	42<br>41<br>40<br>22 mm	[717]
				Diffusion<br>30°C<br>72–96h<br>pH NM<br>Inoculum size NM<br>Nystatin 50 µg	*C. albicans* MTCC 227<br>*A.niger* MTCC 281	34<br>20 mm	

Adhatoda vasica	Leaves	Zinc acetate 100 mM/ plant extract 5 % (5:1 v/v)<br>100°C<br>1 h<br>pH10	Hexagonal<br>10–12 nm	Dilution<br>37°C<br>24h<br>pH NM<br>1×10^8 CFU/ml<br>Streptomycin 10 µg	*S. epidermidis* MTCC 435<br>*E. coli* MTCC 443<br>*P. aeruginosa* MTCC 741<br>*Staphylococcus spp*<br>*Streptococcus spp*	16<br>16<br>128<br>32<br>128µg/ml	[718]
				Dilution<br>25°C<br>72 h<br>pH NM<br>1×10^6 CFU/ml<br>Fluconazole 10 µg	*Aspergillus fumigatus* MTCC 6594<br>*Trichophyton rubrum* MTCC 296<br>*Microsporum audouinii* MTCC 8197<br>*Fusarium spp*<br>*Candida spp*	32<br>64<br>256<br>64<br>256 µg/ml	
Euphorbia petiolata	Leaves	Zinc nitrate 1000 mM/ plant extract 10 % (5:3 v/v)<br>80°C<br>2 h<br>pH NM	Hexagonal<br>10–12 nm	Diffusion<br>37°C<br>24 h<br>pH NM<br>Inoculum size NM<br>Chloramphenicol**	*E. coli*	12 mm	[719]
Punica granatum	Peel	Zinc nitrate/ plant extract 16 % (1:25 w/v)<br>80°C<br>1 h<br>pH 5	Hexagonal<br>20± 5 nm	Diffusion<br>37°C<br>24 h<br>pH NM<br>1×10^5 CFU/ml<br>Streptomycin**<br>Gentamicin**	MRSA 33591<br>*Proteus vulgaris* ATCC 49132<br>*E. coli* K12	6<br>8<br>9.5 mm	[720]
Swertia chirayita	Leaves	Zinc nitrate 7 mM/ plant extract 3.5 % (1:5 v/v)<br>80°C<br>24 h<br>pH NM	Spherical<br>10 nm	Diffusion<br>35±2 °C<br>24h<br>pH NM<br>Inoculum size NM<br>Tetracycline 5 µg/50 ml<br>Ciprofloxacine 20 µg/50 ml	*S. aureus* ATCC 6538<br>*E. coli* ATCC 8739<br>*Salmonella enterica* MTCC 3858	14<br>23<br>16 mm	[721]

Chelidonium majus	Leaves	Zinc nitrate/ plant extract 1.5 % (1:10 w/v)<br>90°C<br>4 h<br>pH NM	Spherical<br>10 nm	Dilution<br>34±1 °C<br>18 h<br>pH NM<br>1×10^6 CFU/ml<br>Amikacin**	*S. aureus* ACTC 4163<br>*E. coli* ATCC 25922<br>*P. aeruginosa* NCTC 6749	40<br>80<br>120 µg/ml	[722]
				Dilution<br>34±1 °C<br>72 h<br>pH NM<br>2×10^6 CFU/ml<br>Nystatin**	*C. albicans* ATCC 10231<br>*A. niger* ATCC 16404<br>*Trichophyton rubrum* ATCC 28188	120<br>80<br>120 µg/ml	
Prunus yedoensis	Leaves	Zinc nitrate 100 mM/ plant extract 25 % (5:1 v/v)<br>80°C<br>8 h<br>pH NM	Spherical or oval<br>10–40 nm	Diffusion<br>37°C<br>24 h<br>pH NM<br>Inoculum size NM<br>Penicillin G 10 µg	*S. epidermidis* KACC 13234<br>*Brevibacterium linens* KACC 14346	24<br>26 mm	[723]
Sechium edule	Leaves	Zinc acetate 5 mM/ plant extract 8 % (4 :1 v/v)<br>60°C<br>2 h<br>pH NM	Spherical<br>36 nm	Diffusion<br>37°C<br>24 h<br>pH NM<br>1×10^4 CFU/ml<br>Streptomycin**	*B. subtilis*<br>*K. pneumoniae*	12<br>14 mm	[724]
Parthenium hysterophorus	Leaves	Zinc nitrate 1 mM/ plant extract 10 % (1:9 v/v)<br>18°C<br>15 min<br>pH NM	Spherical and hexagonal<br>27–84 nm	Diffusion<br>37°C<br>24 h<br>pH NM<br>Inoculum size NM<br>Norfloxacin**	*S. aureus*<br>*B. subtilis*<br>*K. pneumoniae*<br>*E. coli*<br>*Enterobacter aerogenes*	11<br>10<br>14<br>20<br>36 mm	[725]
Ruta graveolens	Stem	Zinc nitrate 100 mM/ plant extract 1 % (9:1 v/v)<br>60°C<br>4–5 h	Spherical<br>20–30 nm	Diffusion<br>37°C<br>36 h<br>pH NM<br>Inoculum size NM	*Klebsiella aerogenes* NCIM 2098<br>*E. coli* NCIM 5051<br>*S. aureus* NCIM 5022<br>*Pseudomonas desmolyticum* NCIM 2028	2.3<br>2.7<br>6.3<br>3.7 mm	[726]

		pH 12		Ciprofloxacin 5 µg/50 ml			
Coptidis rhizoma	Rhizome	Zinc nitrate 5 mM/ plant extract 10 % (1:1 v/v)<br>37°C<br>1 h<br>pH NM	Rod and spherical<br>3–25 nm	Diffusion<br>37°C<br>24 h<br>pH NM<br>Inoculum size NM<br>No control	*Bacillus megaterium*<br>*Bacillus pumilis*<br>*E. coli*<br>*B. cereus*	10<br>9<br>11<br>9 mm	[332]
Bauhinia tomentosa	Leaves	Zinc sulfate 2 mM/ plant extract 10 % (4:1 v/v)<br>60°C<br>4 days<br>pH NM	Hexagonal<br>22–94 nm	Diffusion<br>37°C<br>24 h<br>pH NM<br>Inoculum size NM<br>Levofloxacin**	*P. aeruginosa*<br>*E. coli*<br>*B. subtilis*<br>*S. aureus*	20<br>20<br>8<br>11 mm	[727]
Cassia fistula	Leaves	Zinc nitrate 1 mM/ plant extract 10 % (1:10 v/v)<br>100°C<br>5 h<br>pH NM	Irregular<br>5–15 nm	Diffusion<br>37°C<br>36 h<br>pH NM<br>Inoculum size NM<br>Ciprofloxacin 5 mg/50 ml	*Klebsiella aerogenes* NCIM 2098<br>*E. coli* NCIM 5051<br>*S. aureus* NCIM 5022<br>*Pseudomonas desmolyticum* NCIM 2028	10<br>5<br>5<br>4 mm	[728]
Berberis aristata	Leaves	Zinc acetate 100 mM/ plant extract 10 % (1:6 v/v)<br>70°C<br>8 h<br>pH 12	Spherical<br>5–25 nm	Diffusion<br>37°C<br>24 h<br>pH NM<br>Inoculum size NM<br>Ciprofloxacin**	*E. coli*<br>*K. pneumoniae*<br>*S. aureus*<br>*B. cereus*<br>*B. subtilis*<br>*Serratia marcescens*<br>*Proteus spp*<br>*S. typhi*	13<br>15<br>18<br>19<br>26<br>25<br>0<br>0 mm	[729]
Passiflora caerulea	Leaves	Zinc acetate 1 mM/ plant extract 20 % (2:1 v/v)<br>60°C<br>1 h<br>pH 12	Spherical<br>37 nm	Diffusion<br>37°C<br>24 h<br>pH NM<br>Inoculum size NM<br>Ampicillin**	*Klebsiella spp*<br>*Enterococcus spp*<br>*E.coli*<br>*Streptococcus spp*	11<br>9.3<br>13<br>11.7 mm	[730]

Vitex trifolia	Leaves	Zinc nitrate/ plant extract 10 %<br>(1:20 w/v)<br>60–80°C<br>2 h<br>pH NM	Spherical<br>30 nm	Diffusion<br>37°C<br>24 h<br>pH NM<br>Inoculum size NM<br>Ciprofloxacin 5 µg	*S. aureus*<br>*B. subtilis*<br>*P. aeruginosa*<br>*Proteus mirabilis*<br>*E. coli*	22.5<br>21.5<br>19.8<br>19.3<br>20.1 mm	[731]
				Diffusion<br>28–35°C<br>24 h<br>pH NM<br>Inoculum size NM<br>Amphotericin B 10 µg	*C. albicans*<br>*Candida tropicalis*	20.3<br>17.8 mm	
Cinnamomum verum	Bark	Zinc nitrate/ plant extract 50 %<br>(1:10 w/v)<br>45–50°C<br>2 h<br>pH NM	Spherical<br>10–30 nm	Diffusion<br>37°C<br>24 h<br>pH NM<br>1.5×10^8 CFU/ml<br>Streptomycin 25 µg	*S. aureus* MTCC 7443<br>*E. coli* MTCC 7410	16.8<br>13.3 mm	[732]
Limonia acidissima	Leaves	Zinc nitrate**/ plant extract 10 %<br>(19:1 v/v)<br>80°C<br>72 h<br>pH 10	Spherical<br>12–53 nm	Diffusion<br>37°C<br>5 days<br>pH NM<br>1×10^6 CFU/ml<br>Erythromycin 15 µg	*S. aureus* MTCC 3160<br>*B. cereus*MTCC 8733<br>*Enterococcus faecalis* ATCC 35550<br>*E. coli* MTCC 433<br>*S. typhi* MTCC 3216<br>*P. aeruginosa* ATCC 25619	15.2<br>0<br>0<br>0<br>15.5<br>13.3 mm	[733]
Rhamnus virgata	Leaves	Zinc nitrate/ plant extract 12 %<br>(3:50 w/v)<br>25°C<br>24 h<br>pH NM	Hexagonal<br>20–30 nm	Diffusion<br>37°C<br>24 h<br>pH NM<br>1×10^8 CFU/ml<br>Oxytetracycline 10 µg	*E. coli* ATCC 15224<br>*S. aureus* ATCC 25923<br>*B. subtilis* ATCC 6633<br>*K. pneumoniae* ATCC 4617<br>*P. aeruginosa* ATCC 9721	24<br>18<br>25<br>15<br>14 mm	[734]
				Diffusion<br>28°C<br>48 h<br>pH NM	*C. albicans* FCBP 478<br>*Mucor racemosus* FCBP 0300<br>*A. niger* FCBP 0918<br>*Fusarium solani* FCBP 0291	22<br>27<br>25<br>24	

				1×10^8 CFU/ml<br>Amphotericin B 10 µg	*A. flavus* FCBP 0064	19 mm	
Cassia auriculata	Flowers	Zinc nitrate/ plant extract 5 % (1:10 w/v)<br>40°C<br>2 h<br>pH NM	Hexagonal<br>50–80 nm	Diffusion<br>37°C<br>24 h<br>pH NM<br>Inoculum size NM<br>Tetracycline 10 µg/ml	*E. coli*<br>*B. subtilis*<br>*K. pneumonia*<br>*S.aureus*<br>*S. typhi*	13<br>17<br>16<br>19<br>13 mm	[735]
Emblica officinalis	Fruit	Zinc nitrate 100 mM/ plant extract 25 % (1:5 v/v)<br>80°C<br>2 h<br>pH 6	Hexagonal<br>35 nm	Diffusion<br>37°C<br>24 h<br>pH NM<br>Inoculum size NM<br>Ciprofloxacin 25 µg	*B. subtilis*<br>*Streptococcus pneumoniae*<br>*S. epidermidis*<br>*K. pneumoniae*<br>*S. typhi*<br>*E. coli*	18<br>16<br>29<br>15<br>15<br>17 mm	[736]
				Diffusion<br>28°C<br>72 h<br>pH NM<br>Inoculum size NM<br>Fluconazol 25 µg	*A. niger*<br>*C. albicans*	0<br>0 mm	
Jatropha curcas	Seed	Zinc acetate 500 mM/ plant extract 10 % (1:3 v/v)<br>60°C<br>1 h<br>pH NM	Hexagonal<br>53 nm	Dilution<br>37°C<br>12 h<br>pH NM<br>Inoculum size NM<br>No control	*S. aureus* ATCC 6538P<br>*S. epidermidis* ATCC 12228<br>*E.coli* ATCC 4157<br>*K. pneumoniae* ATCC 9621	62.5<br>62.5<br>31.3<br>31.3 µg/ml	[737]
Aloe vera	Leaves	Zinc sulfate 250 mM/ plant extract 10 % (1:4 v/v)<br>60 °C<br>3 h<br>pH 10	Spherical, oval and hexagonal<br>8–18 nm	Dilution<br>37°C<br>24 h<br>pH NM<br>Inoculum size NM<br>No control	*E. coli* ESBL 336<br>*E. coli* ATCC 25922<br>*P. aeruginosa* ESBL 621<br>*P. aeruginosa* ATCC 27853<br>MRSA<br>*S. aureus* ATCC 95923	2000<br>2200<br>2300<br>2200<br>2000<br>2200 µg/ml	[422]
Rubia cardifolia	Root	Zinc nitrate 25 mM/ plant extract 5 % (9:1 v/v)	Decahedral<br>14–18 nm	Diffusion<br>37°C<br>24h	*Vibrio parahaemolyticus*<br>*Plesiomonas shigelloides*<br>*Shigella spp*	6<br>16<br>14	[738]

		60–70°C<br>10–15 min<br>pH NM		pH NM<br>1.5×10^8 CFU/ml<br>No control	*P. aeroginosa*<br>*Vibrio alginolyticus*	10<br>12 mm	
Cochlospermum religiosum	Leaves	Zinc nitrate/ plant extract 10 % (1:10 w/v)<br>60–80°C<br>2 h<br>pH NM	Hexagonal<br>76 nm	Diffusion<br>37 ± 2 °C<br>24 h<br>pH NM<br>1.5×10^8 CFU/ml<br>Streptomycin**	*B. subtilis*MTCC 121<br>*S. aureus* MTCC 744<br>*E. coli* MTCC 7410<br>*S. typhi* MTCC 733	20.1<br>22.2<br>20.3<br>19.4 mm	[739]
Viola canescens	Leaves	Zinc nitrate 1 mM/ plant extract 5 % (1:1 v/v)<br>60°C<br>4 h<br>pH NM	Hexagonal<br>26 nm	Diffusion<br>37°C<br>24 h<br>pH NM<br>Inoculum size NM<br>Amoxicillin**	*S. aureus*<br>*E. coli*	10.3<br>7.3 mm	[740]
Vaccinium arctostaphylos	Fruit	Zinc acetate 200 mM/ plant extract 10 % (2:1 v/v)<br>60°C<br>24 h<br>pH 10	Spherical<br>12 nm	Diffusion<br>37°C<br>48 h<br>pH NM<br>1×10^7 CFU/ml<br>No control	*S. aureus* PTCC 1189<br>*E. coli* PTCC 1047	12<br>15 mm	[267]
Musa acuminata	Peel	Zinc nitrate/ plant extract 5 % (1:50 w/v)<br>80°C<br>2h<br>pH NM	Hexagonal<br>350 nm	Diffusion<br>37°C<br>24 h<br>pH NM<br>Inoculum size NM<br>No control	*S. aureus*	19 mm	[741]
Aristolochia indica	Leaves	Zinc nitrate/ plant extract 20 % (1:20 w/v)<br>100°C<br>2 h<br>pH NM	Spherical<br>22 nm	Dilution<br>37°C<br>24 h<br>pH NM<br>5×10^5 CFU/ml<br>Vancomycin**<br>Colistin**	*S. aureus* ATCC 29213<br>*P. aeruginosa* ATCC 27853<br>*E. faecalis* ATCC 29212<br>*E. coli* ATCC 25922	100<br>100<br>100<br>200 µg/ml	[742]

Nigella sativa	Seed	Zinc nitrate 50 mM/ plant extract 20 % (10 :1 v/v)<br>45°C<br>72 h<br>pH 8	Hexagonal<br>20 nm	Diffusion<br>37°C<br>24 h<br>pH NM<br>Inoculum size NM<br>Azithromycin 2 µg/ml	*Chromobacterium violaceum*<br>*P. aeruginosa*<br>*E. coli*<br>*Listeria monocytogenes*	32<br>40<br>38<br>14 mm	[70]
Azolla pinnata	Leaves	Zinc nitrate 100 mM / plant extract 10 % (10:1 v/v)<br>100°C<br>2 h<br>pH 12	Spherical<br>12–20 nm	Diffusion<br>37°C<br>24 h<br>pH NM<br>Inoculum size NM<br>No control	*S. aureus*	4 mm	[336]
Justicia adhatoda	Leaves	Zinc nitrate 1 mM/ plant extract 10 % (1:5 w/v)<br>80°C<br>10 min<br>pH 10	Spherical<br>55–83 nm	Diffusion<br>37°C<br>24h<br>pH NM<br>Inoculum size NM<br>No control	*S. aureus*<br>*E.coli*	19<br>21 mm	[743]
Melia dubia	Leaves	Zinc acetate100 mM/ plant extract 10 % (2:1 v/v)<br>60°C<br>2 h<br>pH NM	Hexagonal<br>13 nm	Diffusion<br>37°C<br>24 h<br>pH NM<br>Inoculum size NM<br>No control	*S. aureus* ATCC25933<br>*E. coli* ATCC25922	7<br>8 mm	[744]
Mentha pulegium	Leaves	Zinc nitrate/ plant extract 5 % (1:10 w/v)<br>60–80°C<br>2 h<br>pH NM	Quasi-spherical<br>40 nm	Diffusion<br>37 °C<br>24 h<br>pH NM<br>1×10^6 CFU/ml<br>Ciprofloxacin 5 µg	*S. aureus* ATCC 25923<br>*E. coli* ATCC 25922	23<br>19 mm	[301]
Tabernaemontana divaricata	Leaves	Zinc nitrate/ plant extract 20 % (1:10 w/v)<br>80°C	Spherical<br>20–50 nm	Diffusion<br>37 °C<br>24 h<br>pH NM	*Salmonella paratyphi*<br>*E. coli*<br>*S. aureus*	NM<br>NM<br>NM	[302]

		2 h<br>pH NM		Inoculum size NM<br>No control			
Lavandula vera	Leaves	Zinc sulfate 1 mM/ plant extract 10 % (4:1 v/v)<br>80°C<br>30 min<br>pH NM	Spherical<br>30–80 nm	Dilution<br>37°C<br>24 h<br>pH NM<br>1×10^5 CFU/ml<br>Tobramycin 10 µg<br>Nalidixic acid 30 µg<br>Ceftriaxone 30 µg<br>Cciprofloxacin 5 µg<br>Bacitracin 0.04 unit<br>Vancomycin 30 µg<br>Cephalexin 30 µg<br>Cefixime 5 µg<br>Gentamicin 10 µg<br>Tetracycline 30 µg<br>Amikacin 30 µg<br>Streptomycin 10 µg<br>Amoxicillin 25 µg<br>Cloxacillin 5 µg<br>Erythromycin 15 µg<br>Methicillin 5 µg<br>Imipenem 10 µg<br>Azithromycin 15 µg<br>Kanamycin 30 µg	*S. aureus*<br>*P. aeruginosa*<br>*Proteus mirabilis*	2.6<br>2.6<br>2.6 µg/ml	[745]
Pelargonium zonale	Leaves	Zinc oxide/ plant extract 10 % (1:10 w/v)<br>150°C<br>2h<br>pH NM	Hexagonal<br>61 nm	Diffusion<br>37°C<br>24 h<br>pH NM<br>Inoculum size NM<br>No control	*S. aureus*<br>*E. coli*	15<br>10 mm	[746]
Ailanthus altissima	Fruit	Zinc nitrate 5 mM/ plant extract 1 % (1:1 v/v)	Spherical<br>5–40 nm	Diffusion<br>37°C<br>24 h	*E. coli*<br>*S. aureus*	16<br>20 mm	[747]

		80°C<br>4 h<br>pH NM		pH NM<br>Inoculum size NM<br>Chloramphenicol**			
Sutherlandia frutescens	Leaves	Zinc nitrate/ plant extract 10 % (2:25 w/v)<br>80°C<br>2 h<br>pH NM	Spherical<br>5–25 nm	Dilution<br>37°C<br>24 h<br>pH NM<br>Inoculum size NM<br>No control	*S. aureus*<br>*E. faecalis*<br>*E. coli*	0.3<br>1.3<br>2.5 mg/ml	[292]
Mirabilis jalapa	Leaves	Zinc acetate 500 mM/ plant extract 10 % (1:1 v/v)<br>60°C<br>2 h<br>pH NM	Spherical<br>13–33 nm	Diffusion<br>37°C<br>24 h<br>pH NM<br>1×10^6 CFU/ml<br>Roxithromycin**	*S. aureus* ATCC 6538<br>*B. subtilis* ATCC 6633<br>*E. coli* ATCC 15224<br>*P. aeruginosa* ATCC 9721<br>*K. pneumoniae* ATCC 4619	18<br>25<br>17<br>20<br>21 mm	[748]
Albizia lebbeck	Stem	Zinc nitrate 10 mM / plant extract 20 % (9:1 v/v)<br>60°C<br>5h<br>pH 12	Irregular spherical<br>66 nm	Diffusion<br>37°C<br>24 h<br>pH NM<br>Inoculum size NM<br>Ciprofloxacin 10 µg	*B. cereus* ATCC 7064<br>*S. aureus* 6538 P<br>*E. coli* O157 H7<br>*K. pneumoniae* ATCC 27738<br>*S. typhi* B 4420	8.8<br>4.5<br>9.1<br>7.3<br>10.6 mm	[749]
Ocimum tenuiflorum	Leaves	Zinc nitrate/ plant extract 10 % (1:10 w/v)<br>60–80°C<br>2h<br>pH NM	Hexagonal<br>14–28 nm	Diffusion<br>37°C<br>24 h<br>pH NM<br>Inoculum size NM<br>Ciprofloxacin 10 µg	*S. aureus*<br>*P. aeruginosa*<br>*E. coli*	13<br>18<br>17 mm	[750]
Costus pictus	Leaves	Zinc nitrate 100 mM/ plant extract 8 % (5:1 v/v)<br>80°C<br>4h<br>pH NM	Hexagonal<br>20–40 nm	Diffusion<br>37°C<br>24 h<br>pH NM<br>Inoculum size NM<br>Ciprofloxacin 10 µg	*S. aureus*<br>*B. subtilis*<br>*E. coli*<br>*Salmonella paratyphi*	10<br>17<br>10<br>12 mm	[751]

				Diffusion<br>37°C<br>24 h<br>pH NM<br>Inoculum size NM<br>Fluconazol 10 µg	*C. albicans*<br>*A. niger*	12<br>16 mm	
Lagenaria siceraria	Leaves	Zinc nitrate/ plant extract 10 % (3:50 w/v)<br>80°C<br>2 h<br>pH 10	Hexagonal<br>120 nm	Diffusion<br>37°C<br>24 h<br>pH NM<br>Inoculum size NM<br>Ciprofloxacin 5 µg	*B. subtilis*<br>*E. coli*<br>*P. aeruginosa*<br>*S. aureus*<br>*S. pyogenes*	14<br>11<br>16<br>16<br>12 mm	[752]
Pithecellobium dulce			Hexagonal<br>34.7 nm		*B. subtilis*<br>*E. coli*<br>*P. aeruginosa*<br>*S. aureus*<br>*S. pyogenes*	15<br>17<br>19<br>19<br>15 mm	
Artabotrys hexapetala	Leaves	Zinc nitrate 500 mM/ plant extract 10 % (1:1 v/v)<br>80°C<br>2 h<br>pH NM	Spherical<br>15–20 nm	Diffusion<br>37°C<br>24 h<br>pH NM<br>Inoculum size NM<br>No control	*Streptococcus pneumoniae*<br>*Serratia marcescens*	5<br>13 mm	[753]
Bambusa vulgaris			Hexagonal<br>20–50 nm		*Streptococcus pneumoniae*<br>*Serratia marcescens*	6<br>15 mm	
Nyctanthes arbor-tristis	Flowers	Zinc acetate 10 mM/ plant extract 10 % (1:5 v/v)<br>60°C<br>2 h<br>pH 12	Hexagonal<br>32 nm	Dilution<br>28°C<br>72 h<br>pH NM<br>1×10^5 CFU/ml<br>No control	*Aspergillus alternata*<br>*A. niger*<br>*Botrytis cinerea*<br>*Fusarium oxysporum*<br>*Penicillium expansum*	64<br>16<br>128<br>64<br>128 µg/ml	[754]
Aegle marmelos	Fruit	Zinc nitrate/ plant extract 5 % (3:10 w/v)	Hexagonal<br>20 nm	Diffusion<br>37°C<br>24 h	*S. aureus*<br>*E. coli*<br>*P. aeruginosa*	6<br>7.5<br>3	[755]

		100°C<br>2h<br>pH NM		pH NM<br>1×10^5 CFU/ml<br>No control	MRSA<br>*K. pneumoniae*<br>*B. subtilis*<br>*S. typhi*	3<br>2<br>12<br>7 mm	
Ziziphus nummularia	Leaves	Zinc nitrate/ plant extract 5 % (1:10 w/v)<br>80°C<br>2 h	Spherical and irregular<br>17.3 nm	Diffusion<br>28°C<br>48 h<br>pH NM<br>6×10^5 CFU/ml<br>Clotrimazol 10 µg<br>Fluconazol 10 µg<br>Itraconazol 30 µg<br>Ketoconazol 30 µg	*C. albicans* ATCC 2091<br>*Candida glabrata* NCIM 3448<br>*Cryptococcus neoformans* ATCC 34664	12.5<br>12<br>15.5 mm	[756]
Lantana aculeata	Leaves	Zinc nitrate/ plant extract 10 % (1:2 w/v)<br>100°C<br>5 h<br>pH NM	Spherical<br>12±3 nm	Diffusion<br>37°C<br>48 h<br>pH NM<br>Inoculum size NM<br>Amphotericin B 10 µg/ml	*A. niger*<br>*Fusarium oxysporum*	21<br>19 mm	[757]
Syzygium aromatum	Flowrs	Zinc acetate/ plant extract 10 % (1:5 w/v)<br>80°C<br>5–10 min<br>pH NM	Hexagonal<br>35nm	Dilution<br>28°C<br>14 days<br>pH NM<br>1×10^6 CFU/ml<br>No control	*Fusarium graminearum*	140 µg/ml	[758]
Ricinus communis	Seed	Zinc nitrate/ plant extract 25 % (1:35 w/v)<br>40±10 °C<br>10–15 min<br>pH NM	Hexagonal<br>12–14 nm	Diffusion<br>37°C<br>24 h<br>pH NM<br>Inoculum size NM<br>No control	*Penicillium expansum*<br>*Aspergillus spp*<br>*Rhizopus spp*	3<br>4<br>0 mm	[759]
Azima tetracantha	Leaves	Zinc sulfate 500 mM/ plant extract 10 % (1:1 v/v)<br>90°C	Spherical<br>18–25 nm	Diffusion<br>28°C<br>60 h<br>pH NM	*A. niger*<br>*C. albicans*	16<br>48 mm	[760]

		4 h<br>pH NM		1×10^{5} CFU/ml<br>No control			
Scadoxus multiflorus	Leaves	Zinc nitrate 1 mM/ plant extract 30 % (4:1 v/v)<br>60° C<br>3 h<br>pH NM	Irregular and spherical<br>31 nm	Standard plate count<br>28°C<br>24 h<br>pH NM<br>Inoculum size NM<br>Carbendazim**	*A. flavus* MTCC873<br>*A.niger*MTCC282	76<br>63 %	[335]
Trianthema portulacastrum	Plant	Zinc sulfate 30 mM/ plant extract 40% (5:2 v/v)<br>25°C<br>10 min<br>pH NM	Spherical<br>25–90 nm	Diffusion<br>37°C<br>24 h<br>pH NM<br>1×10^{8} CFU/ml<br>Chloramphenicol **	*E. coli*<br>*S. aureus*	16<br>16 mm	[19]
				Diffusion<br>25°C<br>7 days<br>pH NM<br>Inoculum size NM<br>Nystatin**	*A.niger*<br>*A. flavus*<br>*A. fumigatus*	45<br>41<br>51 %	
Pithecellobium dulce	Peel	Zinc nitrate 5 mM/ plant extract 10 % (4: 1 v/v)<br>80°C<br>45 min<br>pH NM	Spherical<br>50–70 nm	Dilution<br>37°C<br>72 h<br>pH NM<br>Inoculum size NM<br>Fluconazol	*A. flavus*<br>*A.niger*	63.6<br>43 %	[761]
Costus igneus	Leaves	Zinc acetate 20 mM / plant extract 10 % (10:1 w/v)<br>80°C<br>30 min<br>pH NM	Spherical<br>65–95 nm	Diffusion<br>37°C<br>24 h<br>pH NM<br>Inoculum size NM<br>No control	*S. aureus*<br>*S. epidermis*<br>*E. coli*<br>*K. pneumoniae*	60<br>70<br>48<br>52 %	[762]

Jacaranda mimosifolia	Flowers	Zinc gluconate 0.1 mM/ plant extract 1 % (1:1 v/v)<br>60°C<br>3 h<br>pH 12	Hexagonal<br>4 nm	Standard plate count<br>35±1 °C<br>24 h<br>pH NM<br>1×10^6 CFU /ml<br>No control	*Entrococcus faecium* ATCC 35667<br>*E. coli* ATCC 25922	98<br>92 %	[763]

*MIC=minimal inhibition concentration; ZOI=zone of inhibition; PI=percentage of inhibition

**The quantity or concentration is not mentioned.

NM=not mentioned, MRSA=methicillin-resistant *S. aureus*

Table S4. Green platinum nanoparticles exhibiting antimicrobial activity

**Plant type**	**Part used**	**Operative conditions for synthesis**	**NP characteristics**	**Microbiological analyses (operative conditions)**			**Refs.**
				**Methods,**	**Tested bacteria or fungi**	**MIC, DOI or PI***	

			(shape and size)	incubation temperature,<br>incubation time,<br>pH,<br>inoculum density,<br>positive control			
Spinacia oleracea	Leaves	Hexachloroplatinic acid 20 mM/ plant extract 75 % (2:1 v/v)<br>100°C<br>24 h<br>pH NM	Rod<br>154 nm	Diffusion<br>37°C<br>24 h<br>pH NM<br>1×10^5 CFU/ml<br>No control	*S. typhi* MTCC 098	13 mm	[227]
Prunus yedoensis	Gum	Hexachloroplatinic acid 100 mM/ plant extract 25 % (5:1 v/v)<br>80°C<br>5 h<br>pH NM	Spherical and oval<br>10–50 nm	Diffusion<br>37°C<br>48 h<br>pH NM<br>Inoculum size NM<br>Nystatin	*Phytophthora capsici*<br>*Phytophthora drechsleri*<br>*Didymella bryoniae*<br>*Colletotrichum acutatum*<br>*Cladosporium fulvum*	0<br>0<br><br>0<br>15<br><br>18 mm	[764]
Xanthium strumarium	Leaves	Hexachloroplatinic acid 1 mM/ plant extract 1 % (19:1 v/v)<br>100°C<br>1 h<br>pH NM	Cubic<br>20 nm	Diffusion<br>37°C<br>24 h<br>pH NM<br>Inoculum size NM<br>Azithromycin 30 µg	*E. coli* ATCC 25922<br>*K. pneumoniae* MTCC 930<br>*P. aeruginosa* ATCC 27853<br>*S. aureus* ATCC 25923<br>*B. subtilis* MTCC 441	20<br>19<br>18<br>22<br>19	[765]
				Diffusion<br>37°C<br>72 h<br>pH NM<br>Inoculum size NM<br>Clotrimazol 30 µg	*C. albicans* MTCC 227<br>*Candia tropicalis*<br>*Candida parapsilosis*<br>*A. flavus* MTCC 277<br>*A. niger* MTCC 281	16<br>17<br>15<br>16<br>15 mm	

Jatropa gossypifolia	Leaves	Hexachloroplatinic acid 1 mM/ plant extract 30 % (9:1 v/v)<br>30°C<br>1 h<br>pH NM	Spherical<br>20 nm	Diffusion<br>37°C<br>24 h<br>pH NM<br>Inoculum size NM<br>Tetracyclin 10 μg	*Bacillus licheniformis*<br>*S. epidermidis*<br>*S. aureus*<br>*E. coli*<br>*K. pneumoniae*<br>*P. aeruginosa*	0<br>0<br>10<br>0<br>8<br>7 mm	[766]
Jatropha glandulifera			Spherical<br>100 nm		*Bacillus licheniformis*<br>*S. epidermidis*<br>*S. aureus*<br>*E. coli*<br>*K. pneumoniae*<br>*P. aeruginosa*	0<br>0<br>10<br>0<br>9<br>9 mm	
Phoenix dactylifera	date	Hexachloroplatinic acid 1 mM/ date extract 20 % (5:6 v/v)<br>90 °C<br>20 min<br>pH 8.5	Spherical<br>45 nm	Diffusion<br>37°C<br>24 h<br>pH NM<br>NM<br>No control	*P. aeuriginosa*<br>*S. pyogenes*	32.5<br>35.5 mm	[767]
Combretum erythrophyllum	Leaves	Hexachloroplatinic acid 1 mM/ plant extract 5 % (5:1 v/v)<br>90 °C<br>1 h<br>pH NM	Spherical<br>1.04±0.26 nm	Dilution<br>37°C<br>24 h<br>pH NM<br>Inoculum size NM<br>Streptomycin**	*S. epidermidis* ATCC 14990<br>*Klebsiella oxytoca* ATCC 8724<br>*Klebsiella aerogenes* ATCC 27853	3.1<br>1.6<br>1.6 μg/ml	[768]
Tragia involucrata	Leaves	Hexachloroplatinic acid 1 mM/ plant extract 30 % (9:1v/v)<br>50°C<br>1 h	Spherical<br>10 nm	Diffusion<br>37°C<br>24 h<br>pH NM<br>10^5 CFU/ml<br>Gentamicin**	*E. coli*<br>*S. aureus*	23<br>19 mm	[769]
Atriplex halimus	Leaves	Hexachloroplatinic acid 1.9 mM/ plant extract 5 % (1:10 v/v)	Spherical<br>3 nm	Diffusion<br>37°C<br>24 h	*E. coli*<br>*K. pneumoniae*<br>*B. subtilis*	0<br>17<br>0	[770]

		95°C<br>1 h<br>pH 9.6		pH NM<br>$2×10^8$ CFU/ml<br>No control	*S. aureus*	0 mm	

*MIC=minimal inhibition concentration; ZOI=zone of inhibition; PI=percentage of inhibition

**The quantity or concentration is not mentioned.

NM=not mentioned, MRSA=methicillin-resistant *S. aureus*

Table S5. Green palladium nanoparticles with antimicrobial activity

**Plant type**	**Part used**	**Operative conditions for synthesis**	**NP characteristics (shape and size)**	**Microbiological analyses (operative conditions)**			**Refs.**
				**Methods, incubation temperature, incubation time, pH, inoculum density, positive control**	**Tested bacteria or fungi**	**MIC, DOI or PI***	
Salvia hispanica	Leaves	Palladium chloride<br>100 mM/ plant extract<br>5 % (4:1 v/v)<br>80°C<br>10 min<br>pH NM	Spherical<br>25 nm	Diffusion<br>37°C<br>24 h<br>pH NM<br>Inoculum size NM<br>Gentamycin**<br>Penicillin**	*S. aureus*<br>*E. coli*	25.5<br>21.2 mm	[771]

Diospyros kaki	Leaves	Palladium chloride 1 mM/ plant extract 20 % (1:1 v/v)<br>60-70°C<br>25 min<br>pH NM	Spherical<br>98 nm	Diffusion<br>37°C<br>24 h<br>pH NM<br>10^8 CFU/ml<br>No control	*E. coli* ATCC 25922<br>*S.aureus* ATCC 25923	18<br>10.5 mm	[772]
Santalum album	Leaves	Palladium chloride 1 mM/ plant extract 10 % (9:1 v/v)<br>60°C<br>10 min<br>pH NM	Spherical<br>19 nm	Diffusion<br>37°C<br>24 h<br>pH NM<br>Inoculum size NM<br>Levofloxacin**	*E. coli*<br>*P. aeruginosa*<br>*S. aureus*<br>*B. subtilis*	31<br>30<br>12<br>18 mm	[773]
Solanum nigurum	Leaves	Palladium chloride 0.3 mM/ plant extract 10 % (1:8 v/v)<br>100°C<br>10 min<br>pH NM	Spherical<br>21.55 nm	Diffusion<br>37°C<br>24 h<br>pH NM<br>Inoculum size NM<br>No control	*E. coli*	18 mm	[774]
Sapium sebiferum	Leaves	Palladium chloride 0.003 mM/ plant extract 5 % (5:1 v/v)<br>70°C<br>2 h<br>pH NM	Spherical<br>5 nm	Diffusion<br>37°C<br>24 h<br>pH NM<br>Inoculum size NM<br>Streptomycin**	*S. aureus*<br>*B. subtilis*<br>*P. aeruginosa*	29<br>19<br>11 mm	[775]
Orthosiphon stamineus	Gum	Palladium chloride 1 mM/ plant extract 1 % (1:5 v/v)<br>85°C<br>2 h<br>pH NM	Spherical<br>20 nm	Well diffusion<br>37°C<br>24 h<br>pH NM<br>Inoculum size NM<br>Erythromycin**	*E. coli*<br>*S. aureus*	18<br>19 nm	[776]
Anogeissus latifolia	Gum	Palladium chloride 1 mM/ plant extract 10 % (1:1 v/v)<br>121°C	Spherical<br>5 nm	Diffusion<br>37°C<br>24 h<br>pH NM	*P. aeruginosa* ATCC 27853<br>*S. aureus* ATCC 25923	14.3<br>12 mm	[777]

		30 min<br>pH NM		$1{\times}10^8$ CFU/ml<br>Streptomycin 10 µg			
Couroupita guianensis	Fruit	Palladium chloride 1 mM/ plant extract 5 % (19:1 v/v)<br>30°C<br>9 h<br>pH NM	Spherical<br>5–15 nm	Diffusion<br>37°C<br>24 h<br>pH NM<br>Inoculum size NM<br>Streptomycin 1mg/ml	*S. aureus* MTCC96<br>*E. coli* MTCC1687<br>*Proteus mirabilis* MTCC 425<br>*P. aeruginosa* MTCC 1688<br>*Vibrio cholerae* MTCC 3906<br>*B. cereus* MTCC 1272<br>*S. typhi* MTCC 3917<br>*Micrococcus luteus* MTCC 1809<br>*K. pneumoniae* MTCC 530<br>*Rhodococcus rhodochorous* MTCC 265	12<br>17<br>13<br>12<br>14<br>13<br>15<br>9<br>20<br>13 mm	[778]
Melia azedarach	Leaves	Palladium chloride 1 mM/ plant extract 10 % (10:1 v/v)<br>100°C<br>20 min<br>pH NM	Spherical<br>10–20 nm	Diffusion<br>37°C<br>24 h<br>pH NM<br>$1.5{\times}10^8$ CFU/ml<br>Ofloxacin**<br>Gentamicin**	*B. subtilis* ATCC 6633<br>*S. aureus* MTCC 96<br>*S. pneumoniae* MTCC 1936<br>*E. coli* MTCC 40<br>*Proteus vulgaris* MTCC 7299<br>*P. aeruginosa* MTCC 2642	8.3<br>7.3<br>5.3<br>7.3<br>5.3<br>7.3 mm	[779]
				Diffusion<br>37°C<br>48 h<br>pH NM<br>$1.5{\times}10^8$ CFU/ml<br>Ofloxacin**<br>Gentamicin**	*A. niger*<br>*Fusarium solani*<br>*Nigrospora oryzae*<br>*Trichoderma viride*	0<br>0<br>0<br>0 mm	
Padina boryana	Powder	Tetrachloropalladate 10 mM/ plant extract 5 % (20:1 v/v)<br>60°C<br>2 h<br>pH NM	Spherical<br>8.7 nm	Diffusion<br>37°C<br>24 h<br>pH NM<br>$1{\times}10^8$ CFU/ml<br>Gentamicin**	*S. aureus*<br>*Escherichia fergusonii*<br>*Acinetobacter pittii*<br>*P. aeruginosa*<br>*Aeromonas enteropelogenes*<br>*Proteus mirabilis*	18<br>20<br>23<br>21<br>19<br>23 mm	[780]

Catharanthus roseus	Leaves	Palladium chloride 1 mM/ plant extract 5 % (5:1 v/v)<br>60°C<br>1 h<br>pH NM	Spherical<br>38 nm	Diffusion<br>37°C<br>24 h<br>pH NM<br>Inoculum size NM<br>Streptomycin**	*Proteus mirabilis*<br>*S. aureus*	12<br>11 mm	[781]
Citrullus lanatus	Rind	Palladium chloride 1 mM/ plant extract 2 % (2:1 v/v)<br>30°C<br>24 h<br>pH NM	Spherical<br>96 nm	Diffusion<br>37°C<br>24 h<br>pH NM<br>Inoculum size NM<br>No control	*E. coli*<br>*S. typhi*	29<br>23 mm	[782]
Stachys lavandulifolia	Herbal	Palladium chloride 1 mM/ plant extract 10 % (1:1 v/v)<br>100°C<br>2 h<br>pH NM	Spherical<br>5-7 nm	Diffusion<br>37°C<br>24 h<br>pH NM<br>1×10^6 CFU/ml<br>Chloramphenicol**	*S. aureus*<br>*P. aeruginosa*<br>*B. cereus*	15<br>17<br>19 mm	[783]
Hypericum hookerianum	Leaves	Palladium chloride 1 mM/ plant extract 10 % (1:1 v/v)<br>70°C<br>15 min<br>pH NM	Spherical<br>2-10 nm	Diffusion<br>37°C<br>24 h<br>pH NM<br>Inoculum size NM<br>No control	*B. subtillis*<br>*P. aeruginosa*	17 mm	[784]

*MIC=minimal inhibition concentration; ZOI=zone of inhibition; PI=percentage of inhibition

**The quantity or concentration is not mentioned.

NM=not mentioned, MRSA=methicillin-resistant *S. aureus*

Table S6. Green copper nanoparticles exhibiting antimicrobial activity

Plant type	Part used	Operative conditions for synthesis	NP Characteristics (shape and size)	Microbiological analyses (operative conditions)			Refs.
				Methods,<br>incubation temperature,<br>incubation time,<br>pH,<br>inoculum density,<br>positive control	Tested bacteria or fungi	MIC, DOI or PI*	
Echinops sp.	Roots	Copper (II) nitrate 500 mM/ plant extract 10 % (4:1 v/v)<br>Room<br>24 h<br>pH NM	Spherical<br>20 nm	Diffusion<br>35±2°C<br>18–24 h<br>pH NM<br>Inoculum size NM<br>Chloramphenicol**	*S. aureus*<br>*E. coli*<br>*P. aeruginosa*<br>*E. aerogenes*	13<br>12<br>10<br>12 mm	[785]
Aerva javanica	Leaves	Copper (II) chloride 4 mM/ plant extract 10 % (25:1 v/v)<br>80°C<br>2 h<br>pH NM	Spherical, hexagonal and oval<br>12–14 nm	Diffusion<br>37°C<br>24 h<br>pH NM<br>1×10^8 CFU/ml<br>Norfloxacin**	*E. coli*<br>*P. aeruginosa*<br>*S. aureus*<br>*Acinetobacter baumanni*	5<br>5<br>9<br>5 mm	[786]
Hagenia abyssinica	Leaves	Copper (II) sulfate 5 mM/ plant extract 10 % (10:1 v/v)<br>50°C	Spherical<br>34.8 nm	Diffusion<br>37°C<br>24 h<br>pH NM	*E. coli*<br>*S. aureus*<br>*B. subtilis*<br>*P. aeruginosa*	12<br>14<br>14<br>12 mm	[787]

		24 h<br>pH 7		1.3×10^8 CFU/ml<br>Ampicillin**			
Syzygium aromaticum	Bud	Copper (II) acetate 1 mM/ plant extract 10 % (5:1 v/v)<br>30°C<br>15 min<br>pH NM	Spherical<br>20 nm	Diffusion<br>37°C<br>24 h<br>pH NM<br>Inoculum size NM<br>No control	*Staphylococcus spp*<br>*E. coli*<br>*Pseudomonas spp*<br>*Bacillus spp*	5<br>6<br>7<br>8 mm	[788]
				Diffusion<br>37°C<br>72 h<br>pH NM<br>Inoculum size NM<br>No control	*A. niger*<br>*A. flavus*<br>*Penicillium spp*	5<br>5<br>6 mm	
Ocimum sanctum	Leaves	Copper (II) sulfate 50 mM/ plant extract 10 % (1:1 v/v)<br>60°C<br>10-15 min<br>pH NM	Spherical<br>29 nm	Dilution<br>37°C<br>18–24 h<br>pH NM<br>Inoculum size NM<br>Doxycycline**	*E. coli*<br>*B. subtilis*<br>*C. albicans*	31.2<br>-<br>125 ug	[789]
Parthenium hysterophorus	Leaves	Copper (II) sulfate 1 mM/ plant extract 10 % (5:2 v/v)<br>Room<br>24 h<br>pH NM	Spherical<br>20 nm	Diffusion<br>37°C<br>24h<br>pH NM<br>Inoculum size NM<br>Streptomycin**	*B. subtilis*<br>*S. aureus*	46<br>35	[790]
				Diffusion<br>28°C<br>48–72 h<br>pH NM<br>Inoculum size NM<br>No control	*C. albicans*<br>*A. flavus*	35<br>42 mm	
Gloriosa superba	Leaves	Copper (II) sulfate1 mM/ plant extract 5 % (4:1 v/v)	Spherical<br>5–10 nm	Diffusion<br>37°C<br>24–36 h	*Klebsiella aerogenes* NCIM 2098<br>*E. coli* NCIM 5051<br>*S. aureus* NCIM 5022	15<br>13<br>6	[262]

		60°C<br>3–4 min<br>pH NM		pH NM<br>Inoculum size NM<br>Ciprofloxacin 0.5 µg/µl	*Pseudomonas desmolyticum*<br>NCIM 2028	5 mm	
Cassia auriculata	Leaves	Copper (II) sulfate1 mM/ plant extract 5 % (4:1 v/v)<br>Room temperature<br>5 h<br>pH NM	Clusters<br>38 nm	Diffusion<br>37°C<br>24 h<br>pH NM<br>1×10^8 CFU/ml<br>Amoxicillin**	*E. coli*<br>*P. aeruginosa*<br>*S. aureus*<br>*Proteus mirabilis*<br>*B. cereus*<br>*K. pneumoniae*	16<br>10<br>14<br>16<br>18<br>14 mm	[263]
Bifurcaria bifurcate	Alga	Copper (II) sulfate 1 mM / plant extract 20 % (10:1 v/v)<br>100–120°C<br>24 h<br>pH NM	Spherical<br>5-45 nm	Diffusion<br>37°C<br>24 h<br>pH NM<br>Inoculum size NM<br>Gentamicin**	*Enterobacter aerogenes*<br>*S. aureus*	14<br>16 mm	[791]
Zingiber officinale	Rhizome	Copper (II) sulfate 5 mM/ plant extract 30 % (5:3 v/v)<br>60°C<br>4 h<br>pH NM	Spherical<br>31 nm	Diffusion<br>37°C<br>24 h<br>pH NM<br>Inoculum size NM<br>Ciprofloxacin**	*E. coli*	22 mm	[792]
Nerium oleander	Leaves	Copper (II) sulfate 1 mM/ plant extract 5 % (4:1 v/v)<br>50°C<br>20 min<br>pH NM	Spherical<br>20 nm	Diffusion<br>37°C<br>24 h<br>pH NM<br>Inoculum size NM<br>Gentamicin**	*E. coli*<br>*S. aureus*<br>*K. pneumoniae*<br>*S. typhi*<br>*B. subtilis*	10<br>13<br>10<br>18<br>14 mm	[793]
Tilia europaea	Leaves	Copper (II) sulfate 1 mM/ plant extract 20 % (1:4 v/v)<br>80°C<br>25 min<br>pH NM	Spherical<br>4-18 nm	Diffusion<br>37°C<br>24 h<br>pH 7.3<br>Inoculum size NM<br>Cefipime 150 µg/ml	*P. aeruginosa* ATCC 9027<br>*E. coli* ATCC 8739<br>*B. subtilis* ATCC 6633<br>*S. aureus* ATCC 6538P	16<br>13<br>15<br>13 mm	[794]

				Diffusion<br>37°C<br>24 h<br>pH 5.6<br>Inoculum size NM<br>Fluconazole 150 µg/ml	*C. albicans* ATCC 10231	11 mm	
Allium sativum	Leaves	Copper (II) sulfate 10 mM/ plant extract 10 % (5:1 v/v)<br>Room temperature<br>48 h<br>pH NM	Spherical<br>100 nm	Diffusion<br>37°C<br>24 h<br>pH NM<br>Inoculum size NM<br>Ampicillin**	*E. coli*<br>*B. subtilis*	13<br>18 mm	[795]
Gum karaya	Powder	Copper (II) chloride 10 mM/ plant extract 10 % (1:100 v/v)<br>75°C<br>1 h<br>pH NM	Spherical<br>2–10 nm	Diffusion<br>37°C<br>24 h<br>pH NM<br>Inoculum size NM<br>5×10^6 CFU/ml<br>Tetracycline**	*E. coli MTCC 443*<br>*S. aureus MTCC 737*	16<br>14 mm	[796]
Eucalyptus globules	Leaves	Copper (II) sulfate 30 mM/ plant extract 20 % (4:1 v/v)<br>60°C<br>3 h<br>pH 12	Rod<br>17 nm	Diffusion<br>37°C<br>24 h<br>pH NM<br>1×10^7 CFU/ml<br>Amoxicillin**	*E. coli-336*<br>*P. aeruginos- 621*<br>MRSA	25<br>13<br>7 mm	[797]
Tea/ Coffee	*Powder*	Copper (I) nitrate 1 mM/ plant extract 10 % (1:3 v/v)<br>100°C<br>7–8 min<br>pH NM	Spherical<br>5–16 nm	Diffusion<br>37°C<br>24 h<br>pH NM<br>1×10^4 CFU/ml<br>No control	*Shigella dysenteriae*<br>*Vibrio cholera* L 4<br>*Vibrio cholerae* CSK 6669<br>*Streptococcus pneumoniae*<br>*S. aureus*<br>*E. coli*	12<br>12<br>11<br>9<br>12<br>11 mm	[798]

Acalypha indica	Leaves	Copper (II) sulfate 10 mM/ plant extract 20 % (1:1 v/v)<br>100°C<br>7–8 h<br>pH NM	Spherical<br>26–30 nm	Diffusion<br>37°C<br>24 h<br>pH NM<br>Inoculum size NM<br>Tetracycline 10 µg/ml	*E. coli*<br>*Proteus vulgaris*	15<br>9 mm	[799]
				Diffusion<br>37°C<br>48 h<br>pH NM<br>Inoculum size NM<br>No control	*C. albicans*	0 mm	
Moringa oleifera	Leaves	Copper (II) sulfate 3 mM/ plant extract 10 % (1:4 v/v)<br>60°C<br>3 h<br>pH NM	Spherical<br>36-49 nm	Dilution<br>37°C<br>24 h<br>pH NM<br>Inoculum size NM<br>Streptomycin 20 µg/ml	*E. coli*<br>*K. pneumoniae*<br>*S. aureus*<br>*Enterococcus faecalis*	500<br>500<br>500<br>250<br>125 µg/ml	[800]
				Dilution<br>37°C<br>18–24 h<br>pH NM<br>Inoculum size NM<br>Ketoconazole 20 µg/ml	*A.niger*<br>*A. flavus*<br>*C. albicans*<br>*Candida glabrata*	125<br>62.5<br>31.25<br>µg/ml	
Vitis vinifera	Leaves	Copper (II) sulfate 1 mM/ plant extract 10 % (4:1 v/v)<br>Room temperature<br>1 h<br>pH NM	Spherical<br>6 nm	Diffusion<br>37°C<br>24 h<br>pH 7<br>Inoculum size NM<br>Chloramphenicol**	*E. coli*<br>*S. aureus*<br>*B. subtilis*<br>*S. typhi*<br>*K. pneumoniae*	14<br>18<br>12<br>9<br>8 mm	[801]

Botryococcus braunii	Alga	Copper (I) acetate1 mM/ plant extract 2 % (10:1 v/v)<br>100°C<br>24 h<br>pH NM	Cubical, spherical<br>10-70 nm	Diffusion<br>37°C<br>24 h<br>pH NM<br>Inoculum size NM<br>Chloramphenicol**	*P. aeruginosa* MTCC 441<br>*E. coli* MTCC 442<br>*K. pneumoniae* MTCC 109<br>*S. aureus* MTCC 96	17<br>18<br>19<br>22 mm	[802]
				Diffusion<br>25°C<br>24 h<br>pH NM<br>Inoculum size NM<br>Nystatin**	*Fusarium oxysporum* MTCC 2087	12 mm	
Aloe vera	Leaves	Copper (I) nitrate 10 mM / plant extract 25 % (10 :1 v/v)<br>100–120°C<br>24 h<br>pH NM	Spherical<br>20-30 nm	Diffusion<br>37°C<br>24 h<br>pH NM<br>1.5×10^{8} CFU/ml<br>Sulphafurazole 100 µg/ml	*Aeromonas hydrophila* MTCC 646<br>*Pseudomonas fluorescens* MTCC 671<br>*Flavobacterium branchiophilum* ATCC 35036	13<br>15<br>11 mm	[803]
Pterocarpus marsupium	Broth	Copper (II) sulfate 1 mM/ plant extract 5 % (1:1 v/v)<br>50°C<br>15 min<br>pH NM	Spherical<br>40 nm	Diffusion<br>37°C<br>24 h<br>pH NM<br>1×10^{8} CFU/ml<br>Gentamicin**	*K. pneumoniae* MTCC 9751<br>*S. epidermidis* MTCC 2639<br>*B. cereus* MTCC 90<br>*S. aureus* MTCC 9442<br>*P. vulgaris* MTCC 7299<br>*E. coli* MTCC 9721	25<br>20<br>23<br>20<br>22<br>24 mm	[804]
Madhuca longifolia	Seed	Copper (I) nitrate 5 mM/ plant extract 10 % (10:1 v/v)<br>55 °C<br>4 h<br>pH 10	Spherical<br>30 nm	Diffusion<br>37°C<br>24 h<br>pH NM<br>Inoculum size NM<br>Ampicillin 10,000 µg/ml<br>Tetracyclin 10,000 µg/ml	*E. coli* BL21(DE3)<br>*S. aureus*<br>*B. subtilis*	16<br>0<br>15 mm	[805]

Citrus limon/ Turmeric curcumin	Fruit	Copper (II) chloride 1 mM/ plant extract 5 % (1:1 v/v)<br>50-60°C<br>1 h<br>pH NM	Shape NM<br>Size NM	Diffusion<br>37°C<br>24h<br>pH NM<br>2×10^6 CFU/ml<br>Chloramphenicol**	*S. aureus*<br>*B. subtilis*<br>*E. coli*	17<br>21<br>19 mm	[806]
				Diffusion<br>25°C<br>72 h<br>pH NM<br>1×10^5 CFU/ml<br>Fluconazole 1000 µg/ml	*C. albicans*<br>*Curvularia lunata*<br>*A. niger*<br>*Trichophyton simii*	21<br>16<br>19<br>15 mm	
Ficus carica	Leaves	Copper (II) chloride 10 mM/ plant extract 20 % (1:50 v/v)<br>70°C<br>24 h<br>pH NM	Spherical<br>50-120 nm	Diffusion<br>37°C<br>24 h<br>pH NM<br>Inoculum size NM<br>Amoxicillin**	*Pediococcus acidilactici*	17 mm	[807]
Capparis zeylanica	Leaves	Copper (II) sulfate 1 mM / plant extract 5 % (4:1 v/v)<br>60°C<br>24 h<br>pH NM	Spherical<br>60-100 nm	Diffusion<br>37°C<br>24 h<br>pH NM<br>Inoculum size NM<br>No control	*E. coli*<br>*S. aureus*<br>*P. aeruginosa*	11<br>10<br>10 mm	[808]
Hibiscus rosasinensis	Leaves	Copper (I) nitrate 50 mM/ plant extract 10 % (1:1 v/v)<br>Room temperature<br>48 h<br>pH NM	Spherical<br>500 nm	Diffusion<br>37°C<br>16–18 h<br>pH NM<br>Inoculum size NM<br>Ampicillin**	*E. coli*<br>*B. subtilis*	0<br>10 mm	[809]
Dodonaea viscosa	Leaves	Copper (II) chloride 1 mM/ plant extract 20 % (2:1 v/v)	Spherical<br>30-40 nm	Diffusion<br>37°C<br>24 h	*E. coli* MTCC 443<br>*K. pneumonia* NCIM 2079	9<br>14<br>8	[274]

		50°C<br>1 h<br>pH 10		pH NM<br>Inoculum size NM<br>No control	*Pseudomonas fluorescens* MTCC 121<br>*S. aureus* MTCC 4032<br>*B. subtilis* MTCC 441	10<br>9 mm	
Azadirachta indica	Leaves	Copper (II) sulfate 1 mM/ plant extract 10 % (1:1 v/v)<br>Room temperature<br>10 min<br>pH NM	Shape NM<br>Size NM	Diffusion<br>37°C<br>24-48 h<br>pH NM<br>Inoculum size NM<br>No control	*E. coli*<br>*A. niger*	5<br>0 mm	[810]
Lantana camera					*E. coli*<br>*A. niger*	10<br>12 mm	
Tridax procumbes					*E. coli*<br>*A. niger*	10<br>0 mm	
Calotropis procera					*E. coli*<br>*A. niger*	6<br>0 mm	
Phyllanthus amarus	Leaves	Copper (II) sulfate 1 mM/ plant extract 25 % (4:1 v/v)<br>130°C<br>7 h<br>pH NM	Spherical<br>20 nm	Diffusion<br>35± 2°C<br>24 h<br>pH NM<br>1×10^{6} CFU/ml<br>Rifampicin**	*E. coli*<br>*P. aeruginosa*<br>*B. subtilis*<br>*S. aureus*	24<br>25<br>31<br>38 mm	[811]
Cassia occidentalis	Leaves	Copper (I) nitrate 1 mM/ plant extract 10 % (5:1 v/v)<br>Room temperature<br>4 h<br>pH NM	Oval<br>30 nm	Diffusion<br>37°C<br>24 h<br>pH NM<br>Inoculum size NM<br>Ciprofloxacin**<br>Gentamicin**	*S. aureus*<br>*E. coli*<br>*S.typhi*<br>*K. pneumonia*	5<br>8<br>11<br>9 mm	[812]
Oxalis corniculata	Leaves	Copper (II) sulfate 20 mM/ plant extract 10 % (1:1 v/v)<br>37°C<br>6 h<br>pH NM	Spherical<br>78–80 nm	Diffusion<br>37°C<br>24 h<br>pH NM<br>Inoculum size NM<br>Cefixime**	*B. subtilis*<br>*S. aureus*<br>*E. coli*<br>*S. typhi*<br>*A. flavus*<br>*A. niger*	24<br>0<br>21<br>11<br>15<br>17 mm	[813]
Abutilon indicum	Leaves		Spheroid<br>17 nm	Diffusion<br>37°C	*E. coli* ATCC 25922<br>*S. aureus* ATCC 25923	13<br>14	[814]

Clerodendrum infortunatum		Copper (I) nitrate 10 mM/ plant extract 10 % (1:1 v/v)<br>Room temperature<br>2-5 min<br>pH NM	Rod<br>18 nm	24 h<br>pH NM<br>Inoculum size NM<br>Cephradine**	*K. pneumoniae* ATCC 10031<br>*B. subtilis* ATCC 9637	18<br>20 mm	
Clerodendrum inerme			Spheroid<br>21 nm				
				Diffusion<br>37°C<br>24 h<br>pH NM<br>Inoculum size NM<br>Terbinafine**	*A. niger* ATCC 16404<br>*A. flavus* ATCC 9643<br>*Trichoderma harzianum* ATCC 20846	17<br>15<br>24 mm	
Olea europaea	Leaves	Copper (II) acetate 1 mM/ plant extract 10 % (1:2 v/v)<br>60°C<br>2 h<br>pH NM	Spherical<br>42 nm	Diffusion<br>37°C<br>24 h<br>pH NM<br>Inoculum size NM<br>Roxithromycin 2000 μg/ml	*S. aureus* ATCC 6633<br>*Micrococcus luteus* ATCC 10240<br>*S. typhimurium* ATCC 14028<br>*E. coli* ATCC 15224	11<br>10<br>7<br>9 mm	[815]
				Diffusion<br>25°C<br>24 h<br>pH NM<br>Inoculum size NM<br>Clotrimazole**	*Aspergillus fumagatus* FCBP 66<br>*Mucor species* FCBP 0300<br>*A. niger* FCBP 0198<br>*A. flavus* FCBP 0064	8<br>6.5<br>9.7<br>7.6 mm	
Tabernaemontana divaricate	Leaves	Copper (II) sulfate 10 mM / plant extract 20 % (1:1 v/v)<br>100°C<br>7–8 h<br>pH NM	Spherical<br>48±4 nm	Diffusion<br>37°C<br>24 h<br>pH NM<br>Inoculum size NM<br>Tetracycline 50 μg	*E. coli*	17 mm	[816]

Persea americana	Seed	Copper (II) sulfate 1 mM/ plant extract 1 % (2:5 v/v)<br>45-50°C<br>6–7 h<br>pH NM	Spherical<br>42-90 nm	Diffusion<br>Temperature NM<br>Incubation time NM<br>pH NM<br>Inoculum size NM<br>No control	*E. coli*<br>*Streptococcus spp*<br>*Klebsiella spp*<br>*Rhizobacterium spp*	15<br>20<br>12<br>22 mm	[817]
				Diffusion<br>Temperature NM<br>Incubation time NM<br>pH NM<br>Inoculum size NM<br>No control	*A. flavus*<br>*Aspergillus fumigates*<br>*Fusarium oxysporium*	9<br>10<br>8 mm	
Pterospermum acerifolium	Leaves	Copper (I) nitrate 1 mM/ plant extract 10 % (1:1 v/v)<br>45–50°C<br>24 h<br>pH NM	Spherical<br>50 nm	Diffusion<br>37°C<br>24 h<br>pH NM<br>Inoculum size NM<br>Ciprofloxacin**	*B. subtilis*<br>*S. aureus*<br>*E. coli*<br>*S. typhi*	13<br>12<br>11.<br>13 mm	[818]
Polygonum minus	Plant	Copper (I) nitrate 1 mM/ plant extract 3 % (20:1 v/v)<br>60°C<br>5 min<br>pH NM	Spherical<br>20-30 nm	Diffusion<br>37°C<br>24 h<br>pH NM<br>Inoculum size NM<br>No control	*E. coli*<br>*S. aureus*<br>*Aeromonas hydrophilia*	18<br>15<br>14 mm	[819]
Falcaria vulgaris	Leaves	Copper (II) sulfate 40 mM/ plant extract 4 % (1:2 v/v)<br>60°C<br>1h<br>pH 12	Spherical<br>20 nm	Diffusion<br>37°C<br>24 h<br>pH NM<br>1×10^6 CFU/ml<br>Difloxacin 30 µg/ml<br>Chloramphenicol 30 µg/ml<br>Streptomycin 10 µg/ml<br>Gentamycin 10 µg/ml<br>Oxytetracyclin 30 µg/ml<br>Ampicillin 10 µg/ml	*S. typhimurium*<br>*E. coli*<br>*P. aeruginosa*<br>*S. aureus*<br>*S. pneumoniae*<br>*B. subtilis*	28<br>29<br>30<br>30<br>32<br>32 mm	[820]

				Amikacin 25 µg/ml			
				Diffusion<br>37°C<br>24 h<br>pH NM<br>1×10^{6} CFU/ml<br>Fluconazol 60 µg/ml<br>Itraconazol 60 µg/ml<br>Miconazol 60 µg/ml<br>Amphotericin B 60 µg/ml	*C. albicans*<br>*Candida glabrata*<br>*Candida guilliermondii*<br>*Candida krusei*	21<br>22<br>27<br>26 mm	
Tinospora cardifolia	Leaves	Copper (II) chloride 0.25 mM/ plant extract 25 % (5:1 v/v)<br>87°C<br>24 h<br>pH NM	Spherical<br>63 nm	Diffusion<br>37°C<br>24 h<br>pH NM<br>Inoculum size NM<br>No control	*S. aureus* MTCC 3381<br>*E. coli* MTCC 443	23<br>17 mm	[821]
Malva sylvestris	Leaves	Copper (II) chloride 4 mM/ plant extract 1 % (10:1 v/v)<br>80°C<br>2 min<br>pH NM	Spherical<br>5–30 nm	Diffusion<br>37°C<br>24 h<br>pH NM<br>Inoculum size NM<br>No control	*Shigella flexneri*<br>*Listeria monocytogenes*	15<br>18 mm	[822]
Antigonon leptopus	Leaves	Copper (II) sulfate 1 mM/ plant extract 20 % (1:1 v/v)<br>80°C<br>15 min<br>pH NM	Cuboid<br>110–280 nm	Diffusion<br>37°C<br>24 h<br>pH NM<br>5×10^{5} CFU/ml<br>Ampicillin 400 µg/µl	*B. subtilis* MTCC 121<br>*B. licheniformis* MTCC 429<br>*S. aureus* MTCC 96<br>*S. pneumoniae* MTCC 2672<br>*E. coli* MTCC 118<br>*K. pneumoniae* MTCC 2405<br>*P. aeruginosa* MTCC 424<br>*Sphingomonas sanguinis* MTCC 5495	12<br>13<br>11<br>12<br>12<br>10<br>8<br>6 mm	[823]

Citrus reticulata	Peels	Copper (I) nitrate 0.1 mM / plant extract 3 % (9:1 v/v)<br>100°C<br>1 h<br>pH 5	Spherical<br>8–9 nm	Diffusion<br>37°C<br>24 h<br>pH NM<br>1×10^{8} CFU/ml<br>Ampicillin 10 µg	*K. pnemoniae*<br>*P. aeroginosa*<br>*Shigella flexneri*<br>*S. aureus*<br>*Streptococcus pneumoniae*<br>*Campylobacter jejuni*<br>*S. typhimurium*	18<br>12<br>20<br>3<br>18<br>16<br>28 mm	[337]
Citrus sinensis			Spherical<br>16-18 nm		*K. pnemoniae*<br>*P. aeroginosa*<br>*Shigella flexneri*<br>*S. aureus*<br>*Streptococcus pneumoniae*<br>*Campylobacter jejuni*<br>*S. typhimurium*	21<br>23<br>22<br>8<br>27<br>32<br>34 mm	
Citrus limon			Spherical<br>18-30 nm		*K. pnemoniae*<br>*P. aeroginosa*<br>*Shigella flexneri*<br>*S. aureus*<br>*Streptococcus pneumoniae*<br>*Campylobacter jejuni*<br>*S. typhimurium*	3<br>13<br>3<br>2<br>17<br>32<br>19 mm	
Quercus infectoria	Galls	Copper (I) sulfate 5 mM/ plant extract 20 % (17:3 v/v)<br>85°C<br>15 min<br>pH NM	Spherical<br>20 nm	Diffusion<br>37°C<br>24 h<br>pH NM<br>Inoculum size NM<br>Chloramphenicol**<br>Penicillin**	*B. cereus* ATCC 14579<br>*S. aureus* ATCC12600<br>*P. aeruginosa* ATCC 10145<br>*E. coli* ATCC 11175<br>*Acintebacter baumannii* ATCC19606<br>*K. pneumonia* ATCC13883	24<br>31<br>20<br>25<br>23<br>28 mm	[824]
Citrus medica	Fruit	Copper (I) acetate 100 mM/ plant extract 10 % (1:1 v/v)<br>60-100°C<br>24 h	Spherical<br>20 nm	Diffusion<br>37°C<br>24 h<br>pH NM<br>2.18×10^{8} CFU/ml	*E. coli* ATCC 14948<br>*S. typhi* ATCC 51812<br>*Propionibacterium acnes* MTCC 1951<br>*P. aeruginosa* MTCC 4676	26<br>19<br>19<br>21	[825]

		pH NM		No control	*K. pneumoniae* MTCC 4030<br>*Fusarium oxysporum* MTCC 1755<br>*Fusarium culmorum* MTCC 349	25<br>28<br><br>37 mm	
Stachys lavandulifolia	Flowers	Copper (II) chloride 100 mM /plant extract 10 % (2:1 v/v)<br>50°C<br>20 min<br>pH 10	Spherical<br>80 nm	Diffusion<br>37°C<br>24 h<br>pH NM<br>1×10^8 CFU/ml<br>Streptomycin**	*P. aeruginosa*	12 mm	[826]
Magnolia kobus	Leaves	Copper (II) sulfate 1 mM /plant extract 5 % (17:3 v/v)<br>25-95°C<br>24 h<br>pH NM	Spherical<br>50–250 nm	Dilution<br>37°C<br>24 h<br>pH NM<br>22,400 CFU/ml<br>No control	*E. coli* ATCC 25922	99 %	[827]
Millettia pinnata	Flowers	Copper (II) acetate<br>1 mM / plant extract 10 % (4:3 v/v)<br>60°C<br>20 min<br>pH NM	Spherical<br>13–35 nm	Diffusion<br>37°C<br>24 h<br>pH NM<br>Inoculum size NM<br>No control	*P. aeruginosa*<br>*E. coli*<br>*S. aureus*<br>*B. subtilis*	80<br>77<br>78<br>85 %	[828]
Celastrus paniculatus	Leaves	Copper (II) sulfate<br>5 mM / plant extract 10 % (10:1 v/v)<br>60°C<br>20 min<br>pH NM	Spherical<br>2-10 nm	Diffusion<br>37°C<br>24 h<br>pH NM<br>Inoculum size NM<br>No control	*Fusarium oxyporun*	76 %	[829]
Bambusa arundinacea	Leaves	Copper (II) acetate<br>10 mM / plant extract 10% (2:1 v/v)<br>65°C<br>30 min<br>pH NM	Spherical<br>24.4 nm	Diffusion<br>37°C<br>24 h<br>pH NM<br>1×10^8 CFU/ml<br>No control	*B. subtilis*<br>*S. aureus*<br>*E. coli*<br>*P. vulgaris*	19<br>16<br>22<br>19 mm	[830]

*MIC=minimal inhibition concentration; ZOI=zone of inhibition; PI=percentage of inhibition

**The quantity or concentration is not mentioned.

NM=not mentioned, MRSA=methicillin-resistant *S. aureus*

Table S7. Antimicrobial green synthesized iron nanoparticles

**Plant type**	**Part used**	**Operative conditions for synthesis**	**NP characteristics (shape and size)**	**Microbiological analyses (operative conditions)**			**Refs.**
				**Methods,<br>incubation temperature,<br>incubation time,<br>pH,<br>inoculum density,<br>positive control**	**Tested bacteria or fungi**	**MIC, DOI or PI***	
Tridax procumbens	Leaves	Iron (III) chloride 2 mM/ plant extract 15 % (1:1 v/v)<br>Room temperature<br>10 min<br>pH NM	Spherical<br>80–100 nm	Diffusion<br>37°C<br>24 h<br>pH NM<br>Inoculum size NM<br>No control	*P. aeruginosa*	2 mm	[831]
Euphorbia helioscopia	Leaves	Iron nitrate 5 mM/ plant extract 10 % (1:5 v/v)<br>150°C<br>15 min<br>pH NM	Spherical<br>7–10 nm	Diffusion<br>25°C<br>48 h<br>pH NM<br>Inoculum size NM<br>No control	*Cladosporium herbarum*	35 mm	[832]
Glycosmis mauritiana	Leaves	Iron (III) chloride 2 mM/ plant extract 50 % (5:1 v/v)<br>80°C<br>20 min<br>pH 12	Spherical<br>100 nm	Diffusion<br>37°C<br>24 h<br>pH NM<br>Inoculum size NM<br>Norfloxacin 20 µg	*B. cereus*<br>*B. subtilis*<br>*Enterococcus faecalis*<br>*E. coli*<br>*K. pneumoniae*<br>*Micrococcus luteus*<br>*Proteus mirabilis*<br>*P. vulgaris*	11<br>19<br>18<br>19<br>12<br>16<br>11<br>19	[833]

					Pseudomonas fluorescence<br>*Vibrio fluvialis*	18<br>10 mm	
Lawsonia inermis	Leaves	Iron (II) sulfate 10 mM/ plant extract 20 % (5:1 v/v)<br>70°C<br>5 min<br>pH NM	Hexagonal<br>21 nm	Diffusion<br>37°C<br>24 h<br>pH NM<br>Inoculum size NM<br>No control	*E. coli*<br>*Salmonella enterica*<br>*Proteus mirabilis*<br>*S. aureus*	14<br>9<br>11<br>15 mm	[834]
Gardenia jasminoides			Hexagonal<br>32 nm		*E. coli*<br>*Salmonella enterica*<br>*Proteus mirabilis*<br>*S. aureus*	15<br>12<br>13<br>16 mm	
Phoenix dactylifera	Fruit	Iron (III) chloride 100 mM/ plant extract 10 % (1:1 v/v)<br>80°C<br>2 h<br>pH NM	Spherical<br>20 nm	Disc diffusion<br>37°C<br>24 h<br>pH NM<br>1×10^{8} CFU/ml<br>Gentamicin**	*S. epidermidis*<br>*K. pneumoniae*<br>*P. aeruginosa*	25<br>24<br>25 mm	[835]
Euphorbia herita	Leaves	Iron (III) chroride 100 mM/plant extract 10 % (2:3 v/v)<br>Room<br>3 h<br>pH 9	Irregular<br>25-80 nm	Diffusion<br>37°C<br>24 h<br>pH NM<br>Inoculum size NM<br>No control	*Bacillus spp*<br>*E. coli*<br>*Pseudomonas spp*	27.7<br>24.3<br>23.7 mm	[836]
				Diffusion<br>28°C<br>48 h<br>pH NM<br>Inoculum size NM<br>No control	*Aspergillus fumigatus*<br>*A. niger*<br>*Arthogrophis cuboida*	21.7<br>18.7<br>25.3 mm	
Carica papaya	Leaves	Iron (III) chroride 100 mM/ plant extract 2 % (1:1 v/v)<br>80°C<br>3h	Spherical<br>31.59 nm	Diffusion<br>37°C<br>24 h<br>pH NM<br>Inoculum size NM	*Klebsiella spp*<br>*E. coli*<br>*Pseudomonas spp*<br>*S. aureus*	9<br>9<br>10<br>12.5 mm	[837]

		pH NM		No control			
Laurus nobilis	Leaves	Iron (III) chroride 100 mM/plant extract 10 % (1:1 v/v)<br>Room<br>2.5 h<br>pH NM	Hexagonal shape<br>8.09 ± 8.99 nm	Diffusion<br>37°C<br>24 h<br>pH NM<br>Inoculum size NM<br>Tetracycline 30 μg<br>Kanamycine 5 μg<br>Sulfamethoxazole 5 μg<br>Amoxicillin 25 μg<br>Cefixime 5 μg<br>Oxacillin 1 μg<br>Penicillin 10 unit<br>Novobiocin 30 μg	*S. aureus* PTCC 1189<br>*E. coli* ATCC 23922<br>*Listeria monocytogenes* PTCC 1294	0<br>0<br>12 mm	[838]
				Diffusion<br>37°C<br>72 h<br>pH NM<br>Inoculum size NM<br>Nystatin 100 μg	*A. flavus* PTCC 5004<br>*Penicillium spinulosum* PTCC 5251	13<br>14 mm	
Lawsonia inermis	Leaves	Iron (II) sulfate 20 mM/ plant extract 10 % (1:1 v/v)<br>60°C<br>30 min<br>pH 11	Spherical<br>150-200 nm	Diffusion<br>37°C<br>24 h<br>pH NM<br>Inoculum size NM<br>No control	*S. aureus*<br>*Staphylococcus typhimurium*	16<br>15 mm	[839]
Spinacia oleracea	Leaves	Iron (III) sulfate 100 mM/ plant extract 20 % (2:1 v/v)<br>60°C<br>30 min<br>pH NM	Spherical<br>10-70 nm	Diffusion<br>37°C<br>24 h<br>pH NM<br>Inoculum size NM<br>Tetracycline**<br>Gentamicin**	*B. subtilis*<br>*E. coli*	23.6<br>20.3 mm	[840]
Musa acuminata	Peel		Spherical<br>20-50 nm		*B. subtilis*<br>*E. coli*	22.7<br>20.5 mm	

Eucalyptus robusta	Leaves	Iron (II) sulfate 5 mM/ plant extract 1 % (2:1 v/v)<br>40°C<br>30 min<br>pH NM	Spherical<br>70 nm	Diffusion<br>35± 2ºC<br>24 h<br>pH NM<br>1×10^8 CFU/ml<br>Gentamicin**	*E. coli*<br>*S. aureus*<br>*P. aeruginosa*<br>*B. subtilis*	1.8<br>1.8<br>1<br>3.9 mm	[841]
Chlorophytum comosum	Leaves	Iron (III) chloride 10 mM/ plant extract 5 % (1:9 v/v)<br>50°C<br>12 h<br>pH NM	Spherical<br>100 nm	Dilution<br>37ºC<br>18–24 h<br>pH NM<br>Inoculum size NM<br>No control	*S. aureus*<br>*E. coli*<br>*P. aeruginosa*<br>*E. faecalis*	6<br>17<br>8<br>9 μg/ml	[842]
Acorus calamus	Rhizome	Iron (III) sulfate 1 mM/ plant extract 1 % (1:1 v/v)<br>32± 2°C<br>3 min<br>pH NM	Spherical<br>20–30 nm	Diffusion<br>37±2°C<br>24 h<br>pH NM<br>5×10^8 CFU/ml<br>No control	*P. aeruginosa*<br>*Enterobacillus spp*<br>*B.subtilis*<br>*E. coli*<br>*S. aureus*	20<br>15<br>20<br>15<br>0 mm	[843]
				Diffusion<br>28±2°C<br>48 h<br>pH NM<br>4×10^7 CFU/ml<br>No control	*Penicillium notatum*<br>*A. niger*<br>*Rhizopus spp*	23<br>20<br>20 mm	
Cynometra ramiflora	Leaves	Iron (II) sulfate 10 mM/ plant extract 10 % (2:1 v/v)<br>40°C<br>20 min<br>pH NM	Spherical<br>20–40 nm	Diffusion<br>30°C<br>24 h<br>pH NM<br>Inoculum size NM<br>No control	*E. coli*<br>*S. epidermidis*	28<br>26 mm	[844]
Aloe vera	Leaves	Ferric chloride 1 mM/ plant extract 20 % (1:1 v/v)<br>50– 60°C	Spherical<br>10–17 mm	Diffusion<br>37°C<br>18–24 h<br>pH NM	*S .aureus* ATCC 2593<br>*E. coli* ATCC 25922<br>*P. mirabilis* ATCC 43071<br>*P. aeruginosa* ATCC 27853	15<br>15<br>16<br>16	[845]

		15 min<br>pH NM		$5×10^6$ CFU/ml<br>Streptomycin 10 µg	*S. typhi* ATCC 13311<br>*K. pneumoniae* ATCC 700603<br>*Shigella flexneri* ATCC 12022<br>*Serratia marcescens* ATCC 27137<br>*Enterococcus faecalis* ATCC 29212	16<br>17<br>14<br>15<br>15 mm	
Agrewia optiva	Leaves	Iron (II) chloride 100 mM/ plant extract 20 % (1:1 v/v)<br>70°C<br>40 min<br>pH NM	Spherical<br>15–60	Diffusion<br>37°C<br>24 h<br>pH NM<br>$1.5×10^8$ CFU/ml<br>Ciprofloxacin 5 µg<br>Gentamicin 10µg	*S. aureus*<br>*Streptococcus mutans*<br>*S. pyrogenes*<br>*Clostridium diphtheriae*<br>*Corynebacterium xerosis*<br>*E. coli*<br>*K. pneumoniae*<br>*P. aeruginosa*	12<br>9<br>9<br>12<br>7<br>6<br>7<br>7 mm	[846]
Prunus persica			Spherical<br>3–70 nm		*S. aureus*<br>*Streptococcus mutans*<br>*S. pyrogenes*<br>*Clostridium diphtheriae*<br>*Corynebacterium xerosis*<br>*E. coli*<br>*K. pneumoniae*<br>*P. aeruginosa*	13<br>14<br>13<br>12<br>10<br>12<br>20<br>14 mm	
Papaver somniferum	Pods	Iron (II) sulfate 5mM/ plant extract 5 % (5:1 v/v)<br>85°C<br>2 h<br>pH 5.7	Irregular<br>38 ± 13 nm	Diffusion<br>37°C<br>24 h<br>pH NM<br>$1×10^8$ CFU/ml<br>Ampicillin**<br>Gentamicin**	*B. subtilis* ATCC 6633<br>*S. epidermidis* ATCC 14990<br>*K. pneumoniae* ATCC 4617<br>*P. aeruginosa* ATCC 9721	7<br>8<br>8<br>7 mm	[847]
				Diffusion<br>37°C<br>48 h<br>pH NM<br>$1×10^8$ CFU/ml<br>Amphotericin B**	*Fusarium solani* FCBP 0041<br>*A. flavus* FCBP 0064<br>*Aspergillus fumigates* FCBP 1264<br>*A. niger* FCBP 0198<br>*Mucor mycosis*	19<br>18<br>17<br>17<br>17 mm	

Allium cepa	Root	Iron (II) nitrate 20 mM/ plant extract 15 % (1:1 v/v)<br>90°C<br>60 min<br>pH NM	Cubic<br>12–52 nm	Diffusion<br>37°C<br>24 h<br>pH NM<br>Inoculum size NM<br>No control	*S. aureus*<br>*P. aeruginosa*	17<br>18 mm	[848]
Ricinus communis	Seeds		Hexagonal<br>20–99 nm		*S. aureus*<br>*P. aeruginosa*	21<br>25 mm	
Uvaria chamae	Leaves	Ferric chloride 10 mM/ plant extract 10 % (1:1 v/v)<br>Room temperature<br>10 min<br>pH NM	Spherical<br>40 nm	Diffusion<br>37°C<br>24 h<br>pH NM<br>1.5×10^8 CFU/ml<br>Ampicillin+cloxacillin**	*E. coli*<br>*P. aeruginosa*<br>*B. subtilis*<br>*S. aureus*	11<br>11<br>14<br>15 mm	[849]
				Diffusion<br>37°C<br>24 h<br>pH NM<br>1.5×10^8 CFU/ml<br>Nystatin**	*C. albicans*<br>*A. niger*	17<br>16 mm	
Falcaria vulgaris	Leaves	Iron (III) chloride 100 mM/ plant extract 10 % (1:9 v/v)<br>Room temperature<br>24 h<br>pH NM	Spherical<br>25 nm	Diffusion<br>37°C<br>24 h<br>pH NM<br>1×10^8 CFU/ml<br>Difloxacin 30 µg<br>Chloramphenicol 30 µg<br>Streptomycin 10 µg<br>Gentamycin 10 µg<br>Oxytetracycline 30 µg<br>Ampicillin 10 µg<br>Amikacin 25 µg	*S. typhi* ATCC 14028<br>*E. coli* ATCC 25922<br>*P. aeruginosa* ATCC 27853<br>*S. aureus* ATCC 25923<br>*S. pneumoniae* ATCC 49619<br>*B. subtilis* ATCC 6633	25<br>26<br>27<br>29<br>30<br>31 mm	[850]

				Diffusion<br>37°C<br>24 h<br>pH NM<br>1×10^6 CFU/ml<br>Fluconazole 60 µg<br>Miconazole 60 µg<br>Itraconazole 60 µg<br>Amphotericin B 60 µg<br>Nystatin 60 µg	*C. albicans* PFCC 89<br>*Candida glabrata* ATCC 14028<br>*Candida krusei* PFCC 52951<br>*Candida guilliermondii* PFCC 88	33<br>34<br>37<br>36 mm	
Citrullus colocynth	Seed	Iron (III) chloride 500 mM/ extract 5 % (1:1v/v)<br>70°C<br>25 min<br>pH NM	Spherical<br>6–15 nm	Diffusion<br>30°C<br>24 h<br>pH NM<br>Inoculum size NM<br>No control	*S. aureus*<br>*B. subtillis*<br>*E. coli*<br>*P. aeruginosa*<br>*C. albicans*	95<br>99<br>88<br>90<br>87 %	[851]
Azadirachta indica	Leaves	Iron (II) chloride 100 mM/ plant extract 10 % (1:2 v/v)<br>60°C<br>72 h<br>pH NM	Clusters<br>20–80 nm	Dilution<br>28± 2°C<br>5 days<br>pH NM<br>Inoculum size NM<br>No control	*Alternaria mali*<br>*Botryosphaeria dothidea*<br>*Diplodia seriata*	82.5<br>80.3<br>79.2 %	[852]
Sageretia thea	Leaves	Iron sulfate 1 mM/ plant extract 15 % (1:1 v/v)<br>85°C<br>2 h<br>pH 5.7	Tetragonal<br>30 nm	Diffusion<br>37°C<br>24 h<br>pH NM<br>1×10^8 CFU/ml<br>Gentamicin 10 µg	*E. coli*<br>*B. subtilis*<br>*S. epidermidis*<br>*K. pneumoniae*<br>*P. aeruginosa*	15<br>17<br>19<br>17<br>20 mm	[853]
Passiflora foetida	Leaves	Ferrous sulfate 1 mM/ plant extract 5 % (1:1 v/v)<br>80°C<br>1 h	Spherical<br>10–16 nm	Diffusion<br>37°C<br>24 h<br>pH NM<br>Inoculum size NM	*Vibrio cholera*<br>*Streptococcus pyogenes*<br>*Shigella flexneri*	8<br>10<br>10 mm	[854]

		pH NM		No control				

*MIC=minimal inhibition concentration; ZOI=zone of inhibition; PI=percentage of inhibition

**The quantity or concentration is not mentioned.

NM=not mentioned, MRSA=methicillin-resistant *S. aureus*
